# Redox-neutral, metal-free tryptophan labeling of polypeptides in hexafluoroisopropanol (HFIP)†

**DOI:** 10.1039/d4cb00142g

**Published:** 2024-08-26

**Authors:** Mohammad Nuruzzaman, Brandon M. Colella, Zeinab M. Nizam, Isaac JiHoon Cho, Julia Zagorski, Jun Ohata

**Affiliations:** a Department of Chemistry, North Carolina State University Raleigh North Carolina 27695 USA johata@ncsu.edu

## Abstract

Despite the unmet needs for chemical tools to study biological roles of tryptophan in living systems, there has been a lack of chemical modification methods for tryptophan residues that can be used in cellular environments. Driven by a preliminary computational study of our previous research, this work experimentally examined our hypotheses to translate the metal-catalyzed tryptophan modification method in hexafluoroisopropanol (HFIP) into a metal-free process. While one of the hypotheses merely confirmed the superiority of the thiophene–ethanol reagent developed in the previous report, the second hypothesis resulted in the identification of a trifluoroborate salt and an acidic ionic liquid as alternatives for the catalysis. Labeling of lysates of a human cell line was achieved with the acidic ionic liquid catalyst, where negative impacts of the tryptophan labeling and HFIP medium on the cellular samples were apparently insignificant. Because the labeling process does not require any redox mediators and is a formal redox-neutral reaction, the metal-free approach would be of use for tryptophan biology research potentially related to their various redox roles.

## Abstract 2

This work translated the preceding metal-catalyzed tryptophan modification method into a metal-free process, enabling efficient labeling of peptides, proteins, and even cell lysates in hexafluoroisopropanol (HFIP).

## Graphical abstract

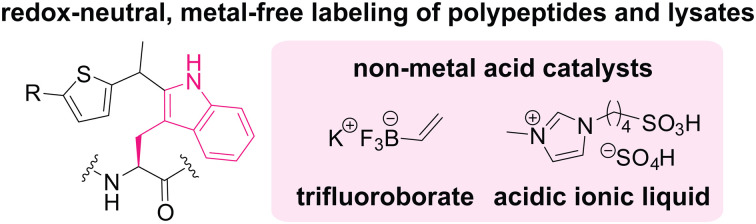

## Introduction

Diverse biological roles of tryptophan residues in proteins have been revealed at a rapid pace, and there is an unmet need for development of chemical tools to study tryptophan biology. As one of the most electron-rich aromatic systems among the major cellular molecules, tryptophan is known to interact with cationic species through cation–π interactions^1^ that can be crucial for certain health conditions such as nicotine addictions.^2^ The aromatic and hydrophobic nature of the amino acid is also critical for unique conformations in polypeptides including Trp cage,^3^ tryptophan zipper,^4^ and tryptophan clasp^5^ through a series of noncovalent interactions. In addition to the supramolecular interaction capabilities, chemical reactivities of tryptophan drives many physiological and pathological processes (*e.g.*, *C*-mannosylation^6,7^ and photoredox^8^ processes). To further reveal the molecular behaviors and roles of the amino acid in proteins, chemical tool development for tryptophan biology at protein and cellular levels is of great importance.

**Fig. 1:**
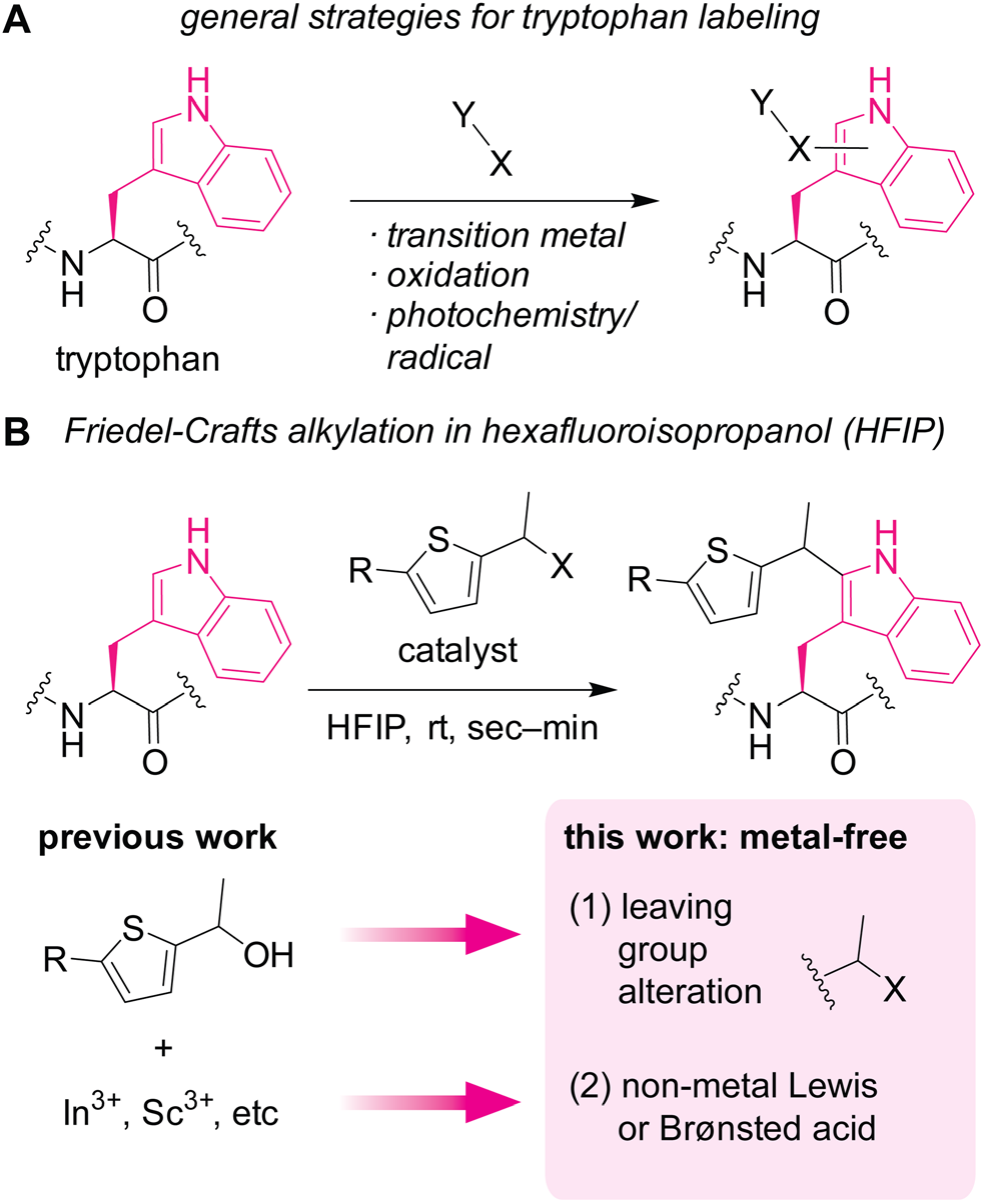
Chemical modification of tryptophan residues in proteins. (A) A scheme showing general strategies for labeling tryptophan residues. (B) Friedel–Crafts alkylation of tryptophan mediated by metal-based (previous work) or non-metal-based (this work) Lewis acid or Brønsted acid in hexafluoroisopropanol (HFIP) as a potentially protein-compatible medium.

Currently available chemical labeling tools for studying tryptophan residues often rely on transition-metal and redox processes (Fig. 1A), which may not always be ideal for cellular samples. Mirroring the nucleophilic behaviors of tryptophan in nature as mentioned above, many of the bioconjugation strategies employ electrophilic reagents for the covalent bond formation.^9^ For example, a seminal example of tryptophan labeling through rhodium carbenoid chemistry leveraged electrophilic reactivity of the carbene intermediate.^10^ Redox chemistry is increasingly studied for tryptophan labeling as well.^11,12^ Despite the significant growth of tryptophan bioconjugation strategies, there remains to be only a handful of methods that are usable for cellular samples.^13,14^ The lack of methods for cellular applications may stem from necessity of bio-incompatible reagents/catalysts/solvents in the majority of the available approaches.^15^ In particular, use of transition metals is common for numerous tryptophan labeling techniques, and metal-free strategies are often critical for chemical tagging in living systems (*e.g.*, the emergence of metal-free bioorthogonal chemistry).^16^ Redox-based labeling technologies have been used for cellular applications,^13,14^ but may not be universally usable for studying tryptophan biology, especially its redox biology,^17^ due to common oxidative side reactions of such redox-labeling methods.

Herein, we demonstrate a redox-neutral, metal-free labeling strategy for tryptophan residues usable for peptide, protein, and cell lysate samples (Fig. 1B). As part of our nonaqueous bioconjugation campaign,^18–21^ we recently reported hexafluoroisopropanol (HFIP)-promoted Friedel–Crafts alkylation of tryptophan residues.^22^ The alkylation reaction with thiophene–ethanol reagents displayed rapid kinetics and enabled radiolabeling and antibody modification applications. Although HFIP can be a denaturing solvent for certain proteins, protein stability enhancement using ionic liquid as an additive was demonstrated, showing that an antibody may have retained its binding capability toward a cellular receptor even after treatment in a mixture of HFIP and ionic liquid. However, the previous method required metal catalysts for efficient labeling, and we discovered that the metal-mediated tryptophan labeling tends to be ineffective for cellular samples (as discussed below in Fig. 5). Taking into account the preliminary computational studies of the preceding work that showed the importance of protonation of the OH group of the thiophene–ethanol reagent, we hypothesized that the rapid Friedel–Crafts alkylation may proceed without metal catalysts as long as the protonation of a leaving group is possible. Thus, two hypotheses were tested in this report: (1) alteration of the OH leaving group to a group with different electronic properties may enable the desired metal-free labeling reaction. (2) Non-metal Lewis acid or Brønsted acid may be able to protonate the OH group of the thiophene reagent. Indeed, one of the hypotheses (hypothesis 2) led to more efficient tryptophan labeling for peptides, proteins, and even cell lysates than the previous metal-mediated version.

## Results and discussion

**Fig. 2:**
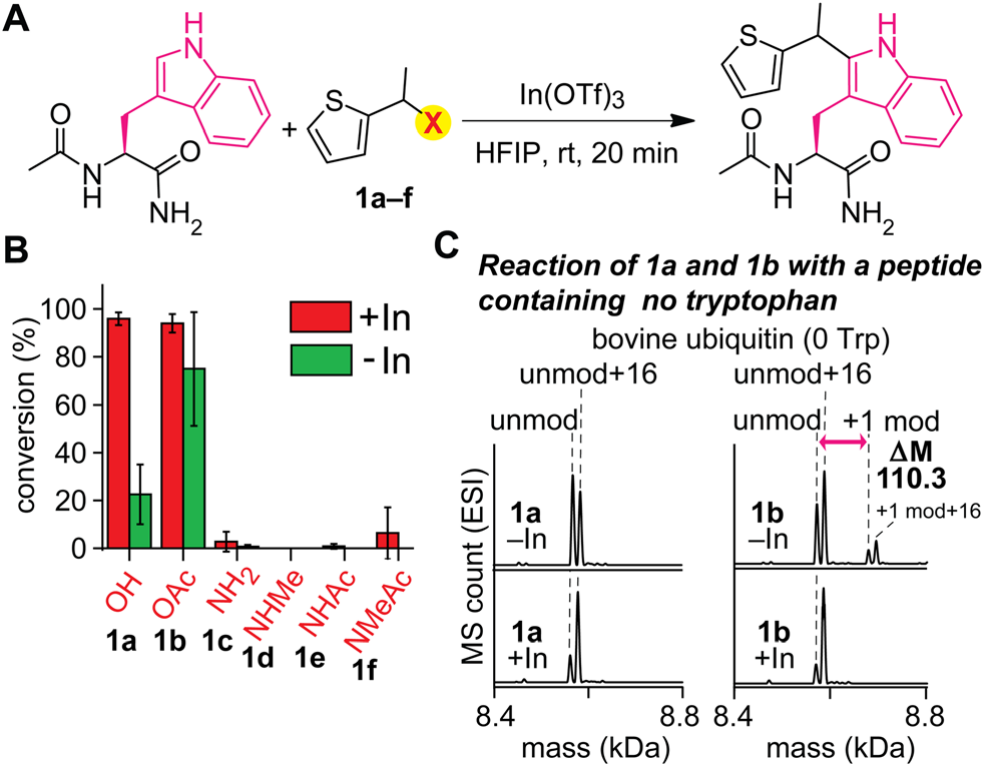
Effect of leaving groups at the benzylic positions of the thiophene reagents for the tryptophan modification. Typical modification conditions: acetyl-tryptophan-amide or ubiquitin (0.1 mM), indium triflate (0.5 mM), and thiophene derivates (3 mM) in hexafluoroisopropanol (HFIP) at room temperature for 20 minutes. (A) General reaction scheme. OTf: triflate ion. (B) Liquid chromatography (LC)-based analysis of modification of acetyl-tryptophan-amide with thiophene-derivatives bearing different leaving groups at the benzylic position. Error bars represent the standard deviation (*n* = 3). (C) Electrospray ionization mass spectrometry (ESI-MS) analysis of modification of bovine ubiquitin that does not have any tryptophan residues with thiophene reagents 1a and 1b in the presence and absence of indium triflate. The peaks right next to both unmod (8566.6 Da) and +1 mod (8676.9 Da) are oxidation products (16 Da additional mass) of unmod and +1 mod respectively. unmod: unmodified ubiquitin. +1 mod: singly modified ubiquitin.

An acylated variant of thiophene–ethanol reagent is capable of metal-free labeling of tryptophan but with compromised chemoselectivity (Fig. 2A and B and Fig. S1, ESI†). Because the reaction mechanism would be dependent on the leaving capability of the thiophene labeling reagent interacting with hexafluoroisopropanol (HFIP) solvent, our initial investigation was focused on the replacement of the OH group with a better leaving group. Using a model amino acid substrate, we compared reactivities of parent thiophene ethanol (1a) and acetylated thiophene ethanol (1b) in the presence and absence of the indium catalyst. Based on liquid-chromatography mass-spectrometry (LC-MS) analysis, acetate variant 1b exhibited fundamentally identical tryptophan modification efficiency as 1a in the presence of the Lewis acid catalyst with retained chemoselectivity to tryptophan (Fig. S2, ESI†). On the other hand, we discovered that substantial modification was observed with 1b even in the absence of the Lewis acid catalyst, whereas 1a showed low conversion. Having confirmed the improved reactivity of the acylated variant on the simple model substrate (acetyl-tryptophan-amide), we then tested chemoselectivity of the reagent using a large peptide bovine ubiquitin, which contains all the 20 canonical amino acids except tryptophan (and cysteine) and should not display any reactivity for the tryptophan labeling process. The mass spectrometry analysis of a reaction mixture showed a mass shift corresponding to the alkylation product when 1b is used in the absence of the metal catalyst, indicating that chemoselectivity of the labeling with 1b is not exclusive to tryptophan residue (Fig. 2C and Fig. S3, ESI†). Presumably the compromised selectivity resulted from the inherent high reactivity of the benzylic position with the acetoxy group that may undergo a simple substitution reaction such as S_N_2 reaction. Interestingly, the acetoxy variant (1b) as well as a pivaloyl variant were not capable of causing the labeling on a small peptide substrate with a single tryptophan residue in the absence of the metal catalyst (Fig. S4 and S5, ESI†), and together, we decided to pursue a different strategy to achieve the metal-free labeling of tryptophan residues because of the limited substrate scope of the acylated variants.

As an alternative approach to modulate the leaving group activity, Lewis basicity of the benzylic group of the labeling reagent was then examined, showing the superior nature of parent thiophene–ethanol reagent (1a) for the tryptophan modification (Fig. 2B and Fig. S1, ESI†). Because the importance of the protonation of the hydroxyl group of the thiophene–ethanol reagent (or the hydrogen bonding acceptor nature of the hydroxyl group) was hinted at by the preliminary density functional theory (DFT) calculations of the preceding work,^22^ we then hypothesized that increasing the basicity of the group may accelerate the protonation process, which may induce the elimination of the leaving group without metal catalysts. To this end, nitrogen variants of thiophene reagents (1c–1f) were synthesized, and their labeling efficiency was evaluated using the model substrate (acetyl-tryptophan-amide) and LC-MS (Fig. 2B). Contrary to our expectation, the nitrogen derivatives displayed diminished efficiency for the trytophan labeling (<10%). Perhaps, the C–N bonds may not be as easily cleavable as the C–O bond of thiophene ethanol (1a) even if the nitrogen protonation by HFIP could be facile.

**Fig. 3:**
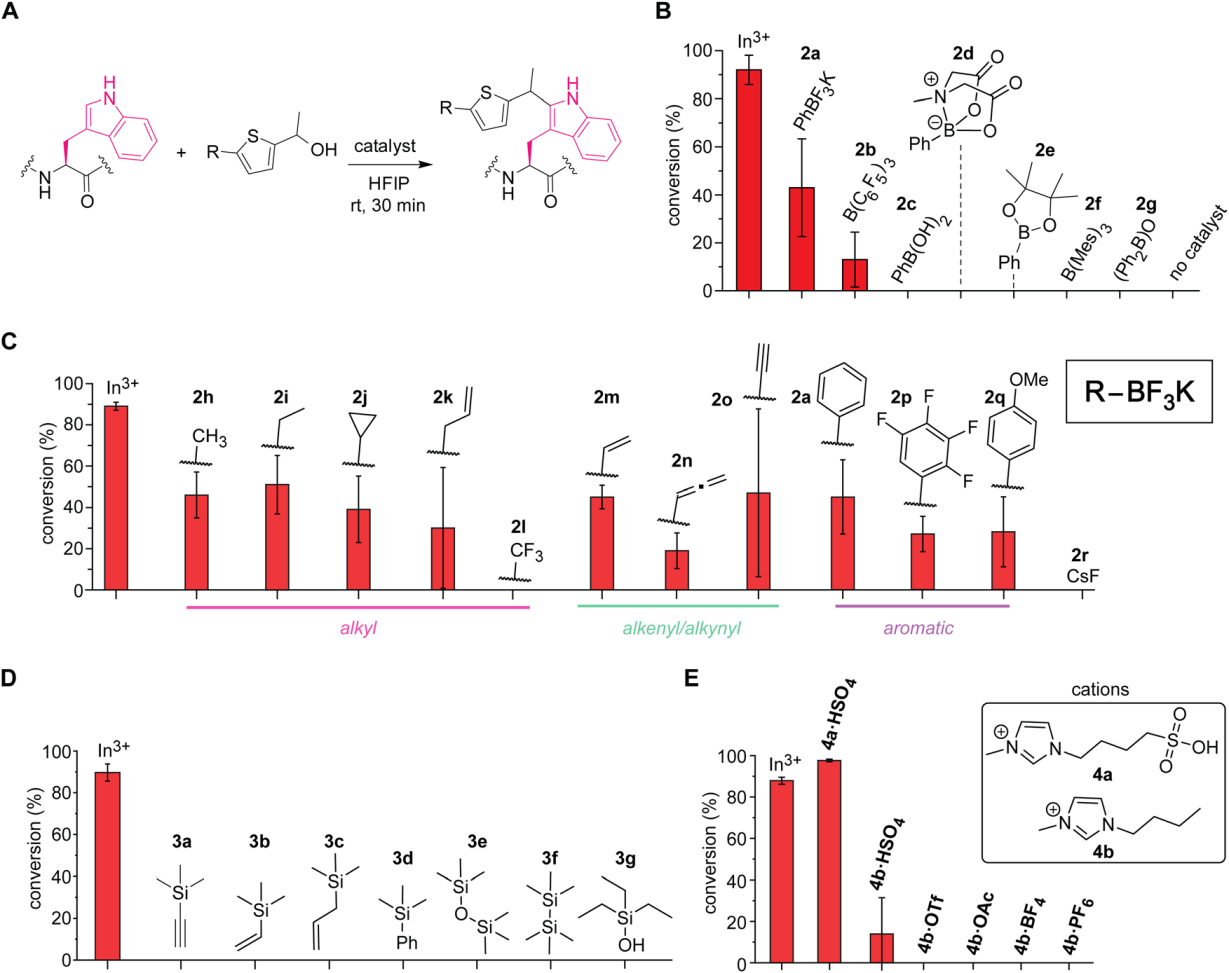
Screening of acid catalysts for the tryptophan labeling thiophene ethanol in hexafluoroisopropanol (HFIP). Typical reaction conditions: somatostatin that contains one tryptophan residue (0.1 mM), catalyst (0.5 mM) and thiophene–ethanol (3 mM), in HFIP at rt for 30 min. Error bars represent the standard deviation (*n* = 3). In^3+^: In(OTf)_3_. Cyclic somatostatin-14 sequence: H-AGCKNFFWKTFTSC–OH with a disulfide bond between the two cysteine residues. (A) Reaction scheme for the catalyst screening process. (B) Liquid chromatography (LC)-based analysis of modification of tryptophan with thiophene-ethanol using a variety of boron-based catalysts. Mes: mesityl or 2,4,6-trimethylphenyl group. (C) LC-based analysis of modification of tryptophan with thiophene ethanol using a variety of organic trifluoroborate catalysts. (D) LC-based analysis of modification of tryptophan with thiophene ethanol using a variety of silicon-based catalysts. (E) LC-based analysis of modification of tryptophan with thiophene ethanol using ionic liquid Brønsted acid catalysts.

Boron-based Lewis acids found to be promising for the tryptophan labeling with the thiophene–ethanol reagents (Fig. 3A and B). As our initial studies based on the first hypothesis regarding the leaving group alteration of the labeling reagent demonstrated the effectiveness of the originally developed thiophene–ethanol structure (Fig. 1B), we then turned our attention to Lewis acid types. Indeed, various boron-catalyzed reactions in HFIP has been reported for small molecule substrates to date,^23^ and we first examined a series of commercially available boron compounds (*e.g.*, borane, boronate, and borinate derivatives) using a model peptide substrate somatostatin (Fig. 3A and B and Fig. S6, ESI†). Although tris(pentafluorophenyl)borane or B(C_6_F_5_)_3_ is a quite common Lewis acid catalyst that has been used for redox-neutral/metal-free Friedel–Crafts reactions,^24^ the borane catalyst (2b) and trimesitylborane (2f) were able to cause only moderate and no meaningful modification, respectively. Borinic acid derivative (2g) with a known, unique catalytic ability^25^ was not an efficient additive for this tryptophan labeling system either. In contrast, we discovered that trifluoroborate salt (2a) showed better activity than any of the tested boron catalysts including boronic acid (2c), boronic acid *N*-methyliminodiacetic acid (MIDA) ester (2d), and boronic acid pinacol ester (2e). Because trifluoroborate salts would not have any energetically accessible empty orbital that acts as a Lewis acid, the higher activity of the trifluoroborate salt is presumably due to dissociation of the fluoride ion generating difluoroboronate species.

Further screening of non-metal Lewis acids and Brønsted acids identified vinyltrifluoroborate salt (2m) and ionic liquid-based Brønsted acid (4a) as potential catalysts for metal-free labeling applications (Fig. 3C–E). Encouraged by the observation of metal-free labeling of a model peptide with phenyltrifluoroborate salt (2a), other trifluoroborates including alkyl, alkenyl, alkynyl, and aromatic derivatives were examined (Fig. 3C and Fig. S7, ESI†). Though there are varying degrees of error ranges across the different substitutions, all the tested trifluoroborate salts showed similar labeling efficiency to phenyl variant (2a), except trifluoromethyl salt (2l) that did not show any observable labeling. Lewis acidity of the boron catalysts would be increased by electron-withdrawing groups such as fluorine atoms, but perhaps the dissociation of the fluoride ion from the trifluoroborate salt to generate the putative active catalyst (difluoroboronate) may be attenuated by the electron-withdrawing groups through stabilization of the negative charge at the boron center. Consistent with this observation, tetrafluorophenyl derivative (2p)—which could exhibit high Lewis acidity^26^—was not a very effective catalyst for the peptide labeling process either. Overall, vinyltrifluoroborate salt (2m) was chosen as the most effective trifluoroborate salt based on the average of the conversion and the relatively small degree of errors in the screening, and the salt was tested for the cell lysate labeling (*vide infra*). Cesium fluoride or CsF (2r) was tested as a negative control to ascertain that fluoride ions derived from trifluoroborate salts are not responsible for the catalysis. In addition to the boron catalysts, silicon-based catalysts were also examined because cation-interacting ability of silyl groups in thiophene–ethanol scaffolds is known,^27^ but none of the tested silicon compounds proved to be useful as catalysts for the tryptophan labeling (Fig. 3D and Fig. S8, ESI†). Moreover, we tested ionic liquid-based Brønsted acids as our previous investigations showed protein stabilization by ionic liquids for bioconjugation purposes.^22,28^ Sulfonic acid-containing imidazolium salt (4a·HSO_4_) indeed displayed higher conversion than that of indium salt (Fig. 3E and Fig. S9, ESI†), which may be another corroborating evidence that the labeling is promoted by protonation of the hydroxyl group of the thiophene–ethanol reagent. The importance of the sulfonic acid group for the labeling was also confirmed by a reaction using bisulfate salt with an imidazolium cation lacking the sulfonic acid moiety (4b·HSO_4_) showing diminished conversion. Other ionic liquids without any acidic groups (*e.g.*, 4b·OTf and 4b·OAc) did not promote the tryptophan labeling, and therefore, the catalytic activity was likely to be enabled by the acidic groups rather than the cations or anions of ionic liquids. Overall, the screening processes allowed for identification of effective catalysts vinyltrifluoroborate (2m) and sulfonic acid-based ionic liquid (4a·HSO_4_) to cause the metal-free tryptophan labeling reaction. Labeling experiments of a model peptide and protein with those catalysts showed reactivity and chemoselectivity toward tryptophan residues as well (Fig. S10, ESI†). The peptide modification proved possible with a stronger inorganic Brønsted acid such as hydrochloric acid, although we found that sulfuric acid was not an effective catalyst as 4a·HSO_4_ (Fig. S11, ESI†), and thus, the ionic liquid acid was chosen in the following study.

**Fig. 4:**
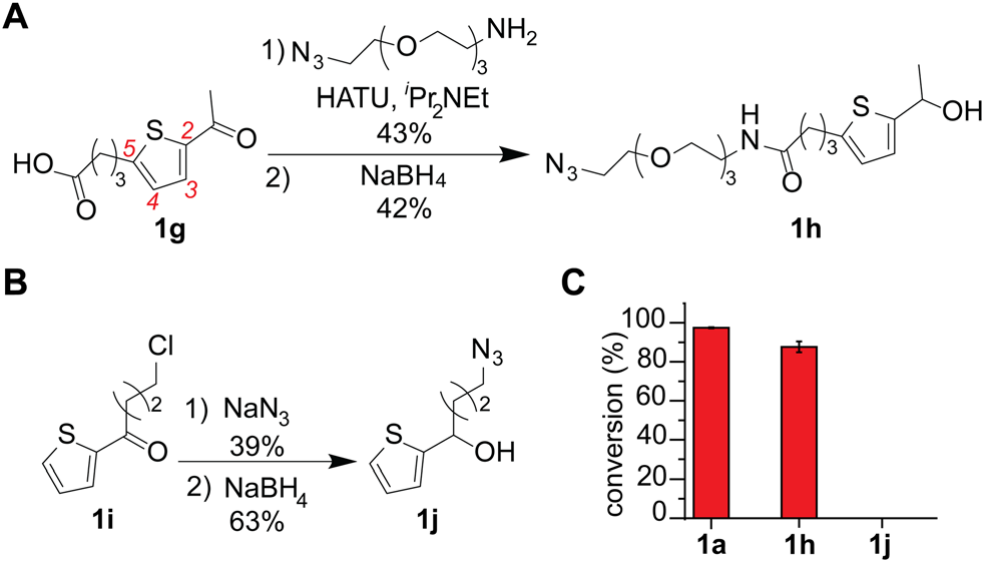
Thiophene–ethanol labeling reagents with azide handles for metal-free tryptophan modification. Peptide modification conditions: somatostatin (0.1 mM), 4a·HSO_4_ (0.5 mM), and azide-containing thiophene–ethanol reagents (3 mM) in hexafluoroisopropanol (HFIP) at rt for 30 min. (A) Synthetic scheme for thiophene–ethanol with an azide handle at the 5 position of thiophene ring. HATU: Hexafluorophosphate azabenzotriazole tetramethyluronium. (B) Synthetic scheme for a thiophene ethanol with an alkylazide moiety originating from the ethanol carbon. (C) Liquid chromatography (LC) analysis of modification of tryptophan-containing substrate (somatostatin) with three different thiophene–ethanol derivatives (1a, 1h and 1j). Cyclic somatostatin-14 sequence: H-AGCKNFFWKTFTSC–OH with a disulfide bond between the two cysteine residues. Error bars represent standard deviation (*n* = 3).

An azide-containing thiophene–ethanol reagent was designed and synthesized for metal-free modification of a complex sample and turned out to be effective for labeling of tryptophan residues on model substrates (Fig. 4). As two non-metal salts were found to be useful for the metal-free tryptophan labeling, we then sought to develop a reagent that can facilitate installation of desired functionality through bioorthogonal chemistry without metal catalysts. Our previous report developed a thiophene–ethanol reagent with an alkyne handle that requires a copper catalyst during the secondary labeling through the azide–alkyne cycloaddition process.^22^ To enable copper-free azide–alkyne cycloaddition, we synthesized azide-containing thiophene–ethanol reagents 1h and 1j. Labeling reagents 1h and 1j were designed based on our previous report identifying the 5-position of the thiophene ring and the ethanol methyl moiety as functionalization compatible sites, respectively.^22^ Synthesis of 1h was achieved through an amide coupling reaction of an azide-containing amine reagent with thiophene–carboxylic acid, followed by hydride reduction of the acetyl moiety to benzyl alcohol (Fig. 4A). The other thiophene–azide reagent (1j) was synthesized by substitution of the alkyl chloride with sodium azide and reduction of the ketone to alcohol (Fig. 4B). Peptide labeling using the synthesized azide-containing thiophene–ethanol reagents under metal-free conditions (4a·HSO_4_ as a catalyst) revealed that a model peptide somatostatin was effectively labeled with 1h, in a similar way to the parent thiophene reagent (1a) (Fig. 3C and Fig. S12, ESI†). Additionally, peptide modification without the catalyst showed no detectable levels of the modified peptide, confirming the reaction's dependence on the acid catalyst (Fig. S12, ESI†). On the other hand, thiophene reagent 1j did not produce any meaningful level of the expected modification product, which is inconsistent with the molecular design based on the previous report (indicating insignificant decrease of the modification efficiency by instillation of a group on the alkyl moiety of the thiophene–ethanol scaffold).

**Fig. 5:**
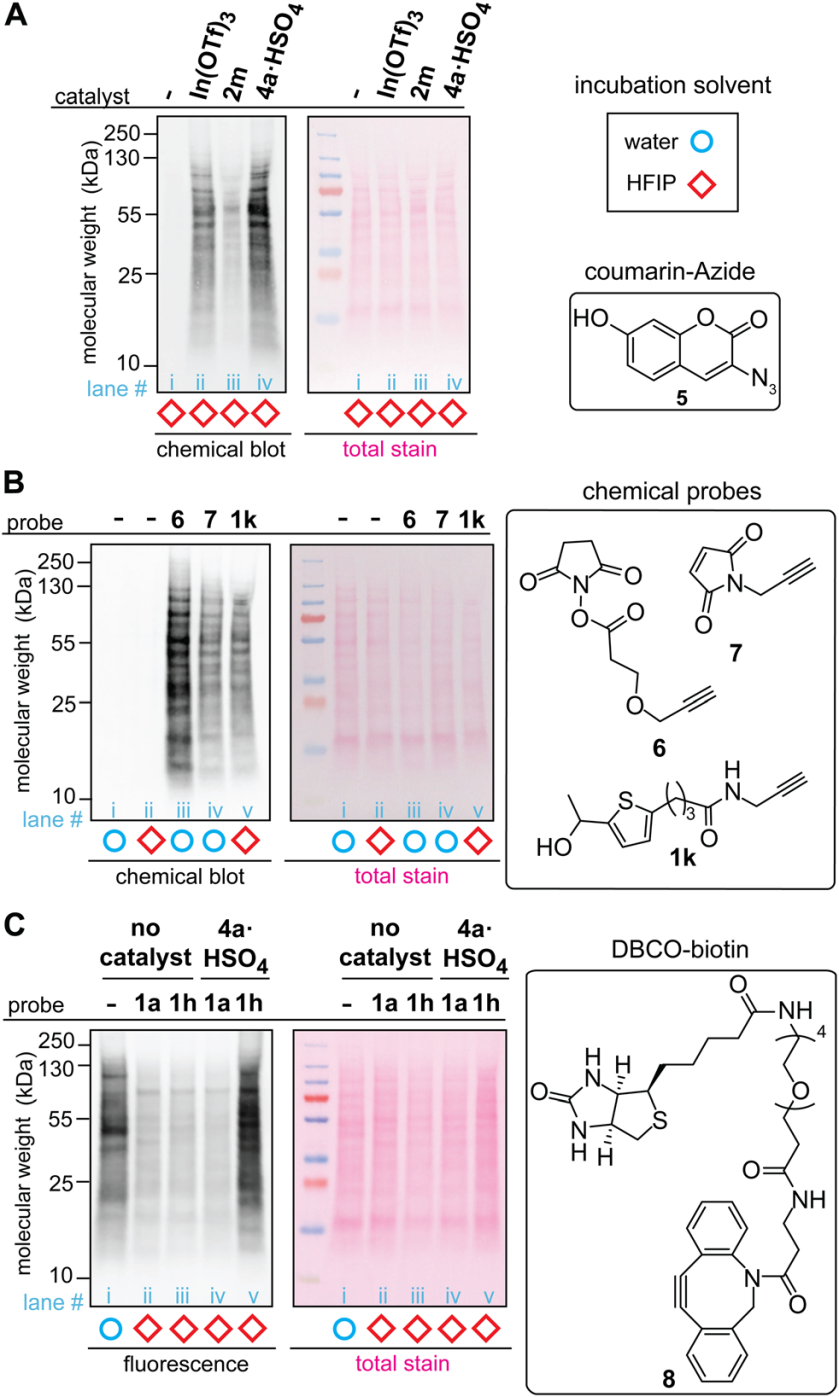
Labeling of HEK293T cell lysate using thiophene ethanol derivatives with acid catalysts. Typical reaction conditions: cell lysate (0.1375 mg mL^−1^), acid catalyst (0.5 mM), thiophene reagent (3 mM) in hexafluoroisopropanol (HFIP) at rt for 5 min. Chemical-blotting conditions for detection of the alkyne handle on a blot membrane: sodium ascorbate (1.5 mM), THPTA (0.1 mM), fluorogenic probe 5, (0.015 mM), CuSO_4_ (0.075 mM), in 1 : 1 DMSO/H_2_O for 30 min.^29^ (A) Chemical blot analysis of cell lysate labeling with 1k in HFIP in the presence of: no catalyst (lane i), In(OTf)_3_ (lane ii), trifluoroborate 2m (lane iii), and ionic liquid 4a·HSO_4_ (lane iv). (B) Chemical blot analysis using fluorogenic probe 5 and comparison between common labeling techniques. Lysine-targeted labeling (lane iii, 0.5 mM of 6), cysteine-targeted labeling (lane iv, 3 mM of 7), and tryptophan-targeted labeling 1k (lane v, 3 mM of 1k). As control experiments, cell lysates that were treated in water and HFIP without any labeling reagents are shown in lane i and ii, respectively. (C) Anti-biotin western blot analysis of the cell lysate labeled with thiophene–azide reagent (1h) and cyclooctyne–biotin reagent (8, DBCO–biotin). All the lysates were treated with 8 (3 mM) for 1 h at rt in 1X LDS loading buffer after the tryptophan labeling. Lane i: cell lysate in aqueous solution labeled only with 8 (without any HFIP and thiophene treatments), lane ii: thiophene ethanol (1a) without acid catalyst, lane iii: thiophene–azide (1h) without acid catalyst, lane iv: 1a with 4a·HSO_4_, and lane v: 1h with 4a·HSO_4_.

The acidic ionic liquid can be an efficient catalyst for tryptophan labeling of cell lysate in HFIP without substantial negative impacts on the cellular sample (Fig. 5A and B). In order to compare the labeling efficiency by different catalysts for a cellular sample, a cell lysate of human embryo kidney (HEK293T) was chosen as a model system. Visualization of the modification efficiency was done using alkyne-tagged thiophene reagent (1k as the structure shown in Fig. 5B) through the attachment of the fluorogenic coumarin–azide (5) on a blot membrane to the alkyne handle introduced by the tryptophan labeling (*i.e.*, visualization of an alkyne handle of proteins on a blot membrane through “chemical blotting”).^29^ In other words, through copper-catalyzed azide–alykne cycloaddition reactions of cell lysate on a blot membrane, the degree of the alkyne installation to cell lysates would be proportional to fluorescence signals of the coumarin fluorophore. A stock solution of cell lysate in a lysis buffer (phosphate-buffered saline containing surfactants and protease inhibitors) was diluted to the HFIP solution for tryptophan labeling with the final volume of water less than 5% during the labeling. After the tryptophan labeling, lithium dodecyl sulfate (LDS) buffer was added to quench the reaction, and volatiles including HFIP were removed by a gentle flow of nitrogen, as high concentration of HFIP is detrimental to the protein gel. Then, the cell lysates in the LDS buffer were denatured in the presence of a reducing agent (dithiothreitol) at 95 °C before the samples were loaded to the gel. The chemical blot analysis of the modified cell lysates using three different catalysts showed consistent results with the peptide labeling experiments (Fig. 3) where the strongest fluorescence response was observed from the acidic ionic liquid conditions (Fig. 5A, lane iii). When the tryptophan labeling in HFIP was compared with other labeling reagents targeting different amino acid side chains (*N*-hydroxylsuccinimide or NHS ester 6 for lysine and maleimide 7 for cysteine) in aqueous solutions, the fluorescence (chemical blot) and colorimetric (total stain by Ponceau S) images did not show any substantial difference of the band patterns between the conditions, indicating that the tryptophan labeling in HFIP was likely to have modified abundant proteins of lysates in a similar fashion as the common, established labeling in aqueous solutions did. The data is overall suggesting the potential utility of bioconjugation methods in HFIP for cell lysate samples but it should be noted that we occasionally observed band shape changes of lysate samples treated in HFIP between technical replicates (Fig. S13, ESI†). Analogous to the results of our preceding work using a simple model substrate with a metal Lewis acid catalyst,^22^ the efficiency of the lysate labeling with the ionic liquid acid catalyst is also sensitive to the concentration of water in the reaction system (Fig. S14, ESI†).

The whole processes of the cell lysate labeling of tryptophan and attachment of functionalities through click chemistry can be transition-metal-free using the azide-tagged thiophene reagent. By using the azide reagent (1h) as well as a biotin-tagged cyclooctyne reagent (DBCO–biotin 8), we hypothesized that the biotinylation of cell lysates should be possible, and the extent of the modification can be visualized through anti-biotin western blot analysis with streptavidin–cyanin dye conjugate (streptavidin–Cy5 conjugate). As anticipated, the condition with azide-tagged thiophene reagent 1h in the presence of acid catalyst 4a·HSO_4_ provided strong fluorescence bands (Fig. 5C, lane v) while weak fluorescence was observed for the conditions with untagged thiophene reagent 1a and those without the acid catalyst (lane ii–iv). It is noteworthy that cell lysate treated with cyclooctyne reagent 8 in aqueous solution (without the tryptophan labeling process in HFIP) showed significant fluorescence signals, although this condition was performed as a negative control (Fig. 5C, lane i). This observation may be related to general nonspecific reactivity of cyclooctyne compounds to cellular samples,^30^ and perhaps the weaker intensities of fluorescence from the negative controls of the tryptophan labeling (lane ii–iv) than the aqueous condition (lane i) may be due to suppression of such nonspecific binding of the cyclooctyne reagent in HFIP-containing samples.

## Conclusion

Our hypotheses for development of the metal-free tryptophan labeling strategy in hexafluoroisopropanol (HFIP) led to trifluoroborate- and acidic ionic liquid-mediated methods that are applicable for modification of tryptophan in cell lysates. The success of the non-metal acid-based tryptophan modification may be an indication of importance of the protonation process in the reaction mechanism, hinted at by our preliminary computational study. Our cell labeling data is also highlighting the inherent challenges of metal-mediated methods for cellular samples and importance of metal-free or organocatalytic methods. Fluoroalcohols have been increasingly used as reaction solvents for small molecule synthesis as well as bioconjugation,^31^ but their use for labeling of biomacromolecules and cellular samples is scarce, and therefore, the present method also shows the possibility to expand the boundary of the protein modification strategies. In particular, the redox-neutral, metal-free conditions of the developed method would be potentially useful for studying redox biology of tryptophan residues in proteins.

## Conflicts of interest

The authors declare no competing interests.

## Supplementary Material

CB-005-D4CB00142G-s001
