# Supplementary material for: Redox-neutral, metal-free tryptophan labeling of polypeptides in hexafluoroisopropanol (HFIP)
Source: RSC Chem Biol. 2024 Aug 26;5(10):963–9. doi: 10.1039/d4cb00142g (PMC11368038; doi:10.1039/d4cb00142g)
Supplement: CB-005-D4CB00142G-s001 [file CB-005-D4CB00142G-s001.pdf]

Supplementary Information (SI) for RSC Chemical Biology.
This journal is © The Royal Society of Chemistry 2024

Electronic Supplementary Information
Redox-neutral, metal-free tryptophan labeling of polypeptides in hexafluoroisopropanol (HFIP)

Mohammad Nuruzzaman,† Brandon M. Colella,† Zeinab M. Nizam, Isaac JiHoon Cho, Julia Zagorski, Jun Ohata.*

Department of Chemistry, North Carolina State University, Raleigh, North Carolina 27695, United States.

†These authors contributed equally to this work.

Email: johata@ncsu.edu

Contents

Supporting figures.....2
General information.....24
Materials and reagents.....24
Cell culture.....24
Instrumentation.....24
Experimental procedures.....26
Preparative synthesis of small molecules.....28
Organic synthesis procedures.....28
High-resolution mass spectrometry (HRMS) spectra of synthesized compounds.....32
Infrared (IR) spectra of the synthesized compounds.....34
NMR spectra of synthesized compounds.....38
References for Electronic Supplementary Information.....54

Supporting figures

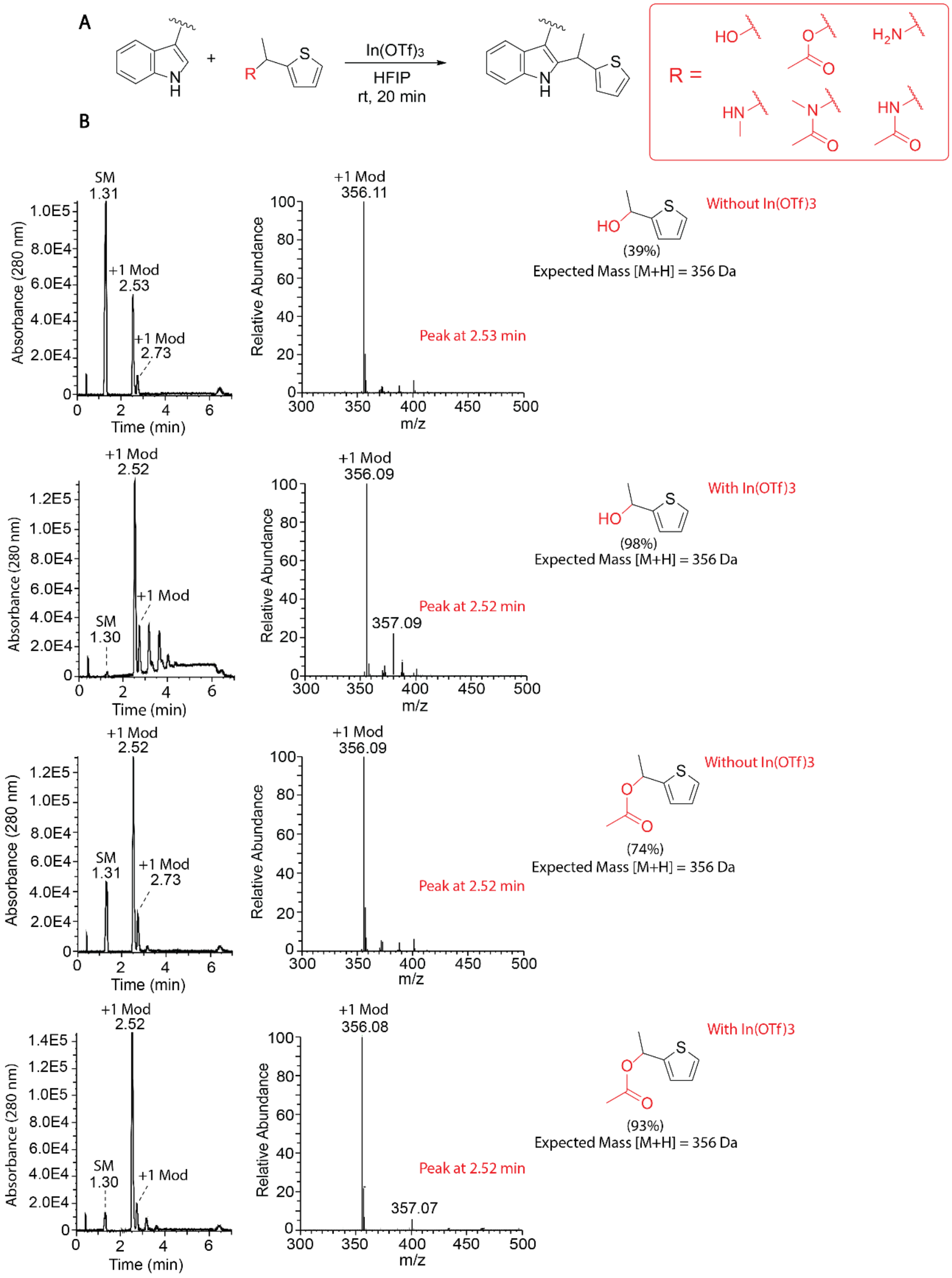

B. (Cont.)

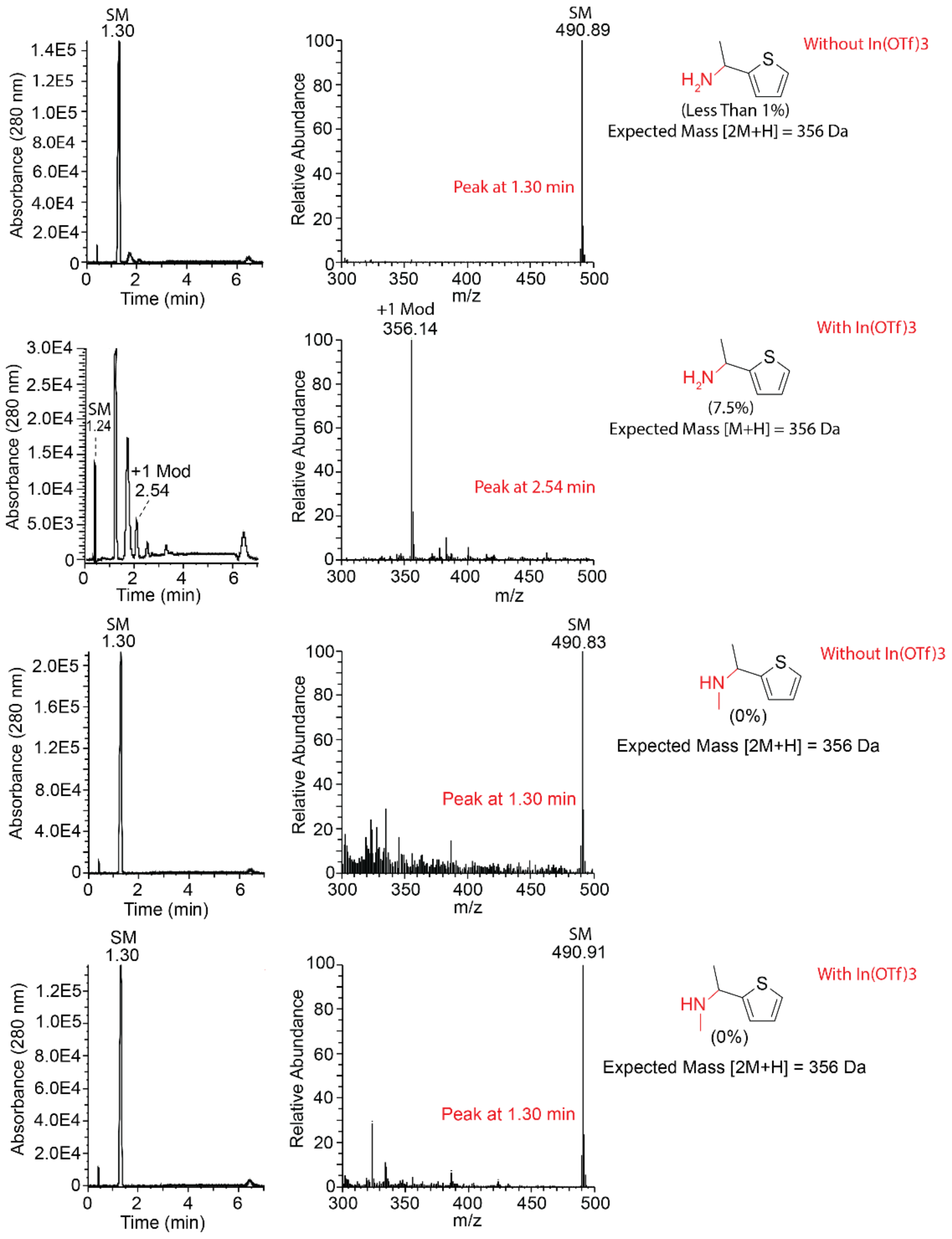
SM
1.30
Absorbance (280 nm)
Time (min)
SM
490.89
Relative Abundance
m/z
Peak at 1.30 min
Without In(OTf)3
H2N
S
(Less Than 1%)
Expected Mass [2M+H] = 356 Da
SM
1.24
+1 Mod
2.54
+1 Mod
356.14
Peak at 2.54 min
With In(OTf)3
(7.5%)
Expected Mass [M+H] = 356 Da
SM
490.83
Without In(OTf)3
HN
(0%)
Expected Mass [2M+H] = 356 Da
SM
490.91
With In(OTf)3
(0%)
Expected Mass [2M+H] = 356 Da

B. (Cont.)

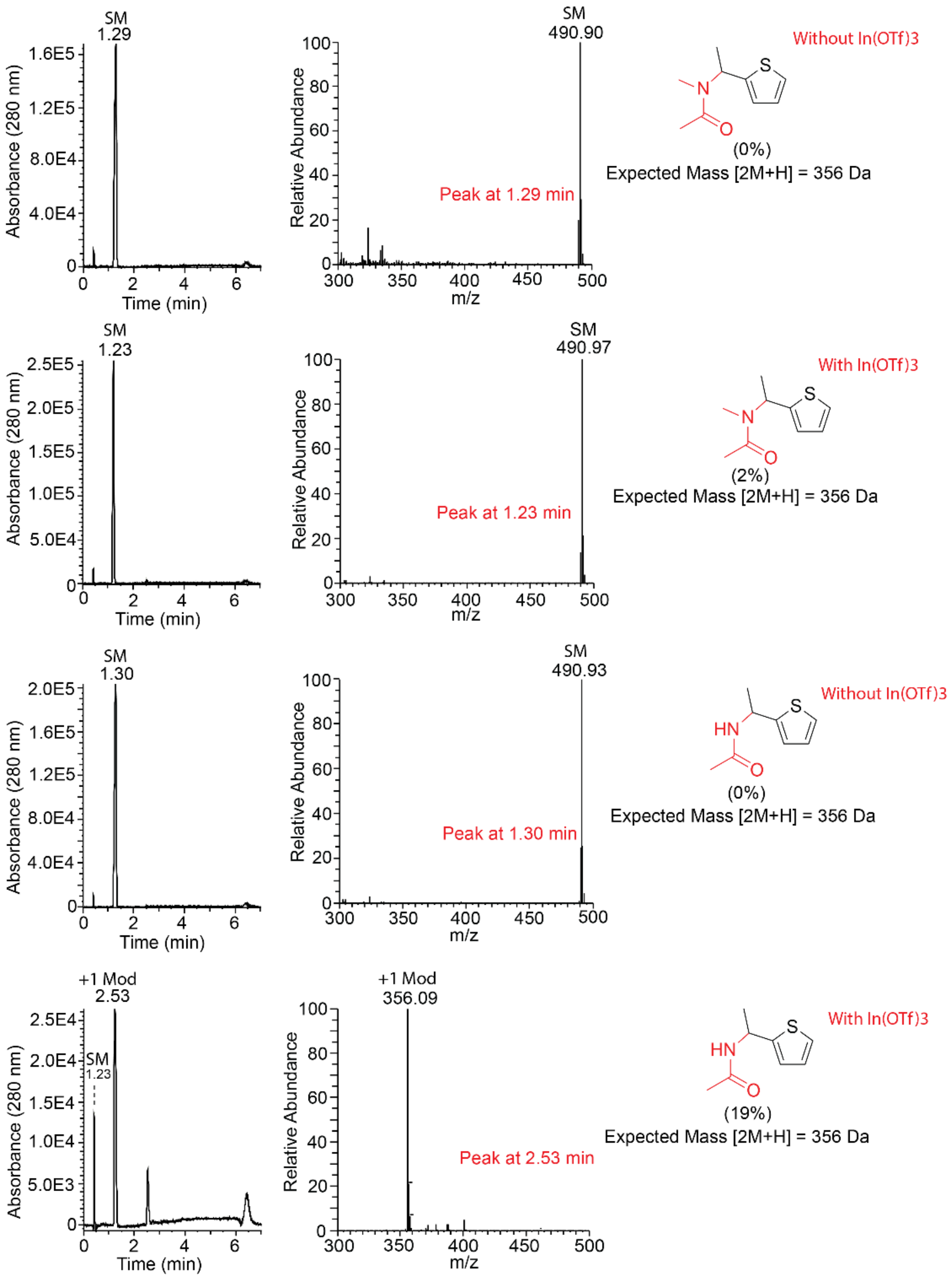

Figure S1. Liquid chromatography-mass spectrometry (LC-MS) analysis of modification of a model amino acid acetyl-tryptophan-amide with thiophene derivatives using indium triflate. Typical reaction conditions: acetyl-tryptophan-amide (0.3 mM), indium triflate catalyst (0.3 mM) and thiophene-derivatives(1.2mM) in HFIP at rt for 20 min. (A) General reaction scheme. (B) LC-MS analysis: UV chromatograms are shown on the left, MS spectra of the starting material (SM) or the modification (+1 mod) are shown in the middle, and % conversion with the expected mass of a modified molecule is shown on the right.

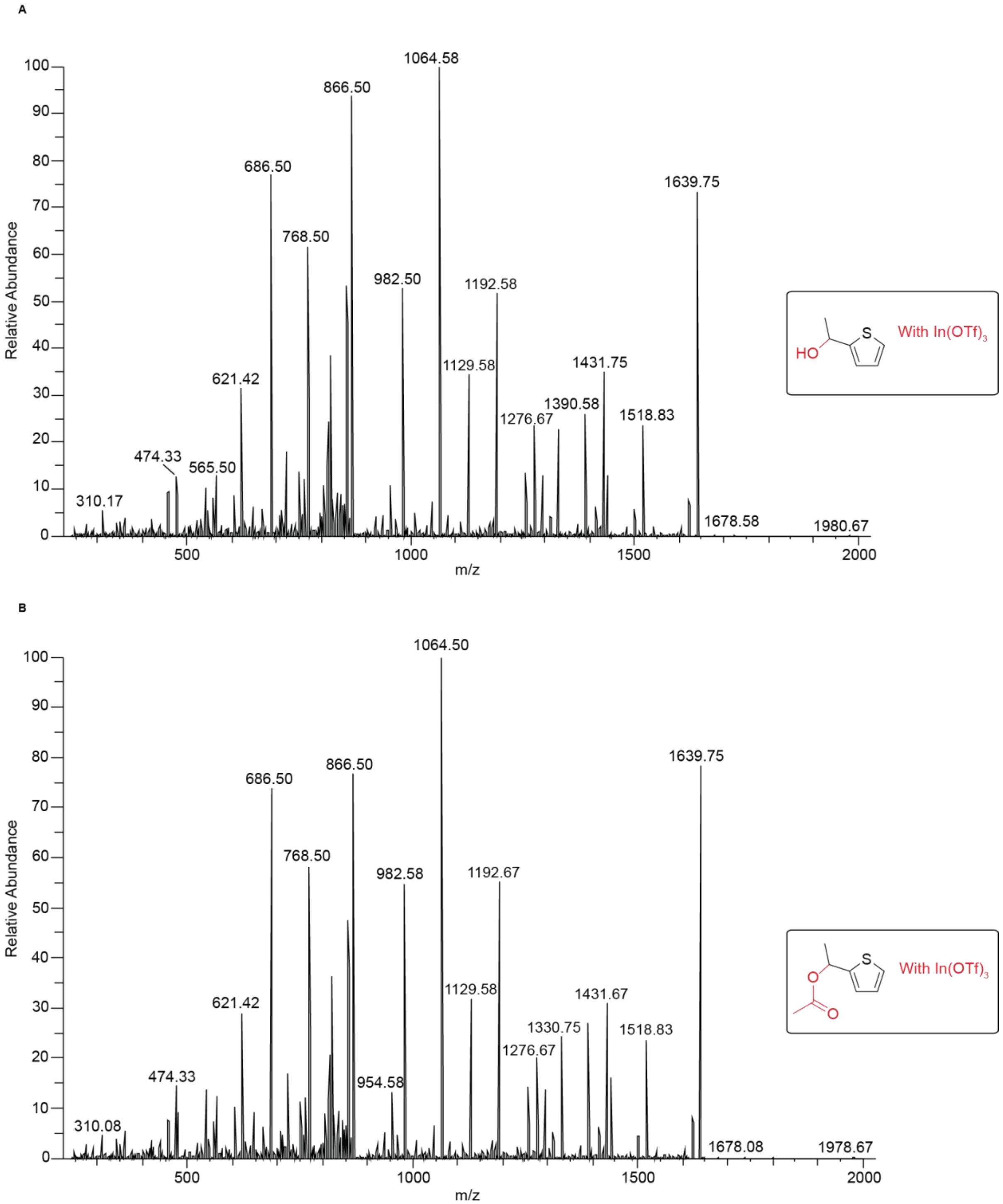

Figure S2. MS/MS spectra of somatostatin modified with thiophene-ethanol (A) and acetylated thiophene-ethanol (B) in the presence of indium catalyst. The spectra are akin to the MS/MS spectrum of a modified somatostatin in a published report (the tryptophan labeling and disulfide reduction of somatostatin prior to MS/MS analysis were performed according to the procedure of the published report).[1]

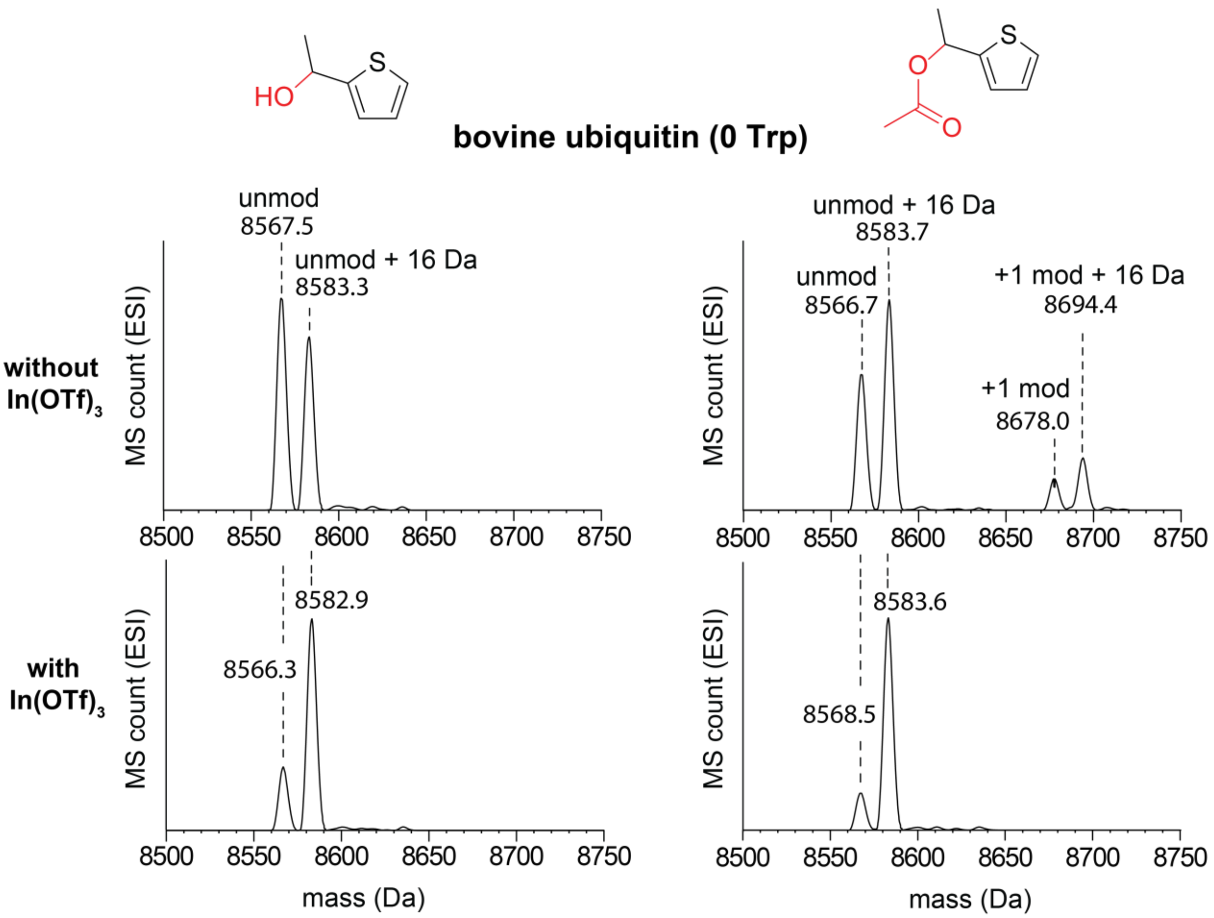

Figure S3. Enlarged mass spectra for protein modification experiments shown in Fig 2C of the main manuscript. Modification conditions: bovine ubiquitin (0.1 mM), indium triflate (0.5 mM), and thiophene derivates (3 mM) in hexafluoroisopropanol (HFIP) at room temperature for 20 minutes.

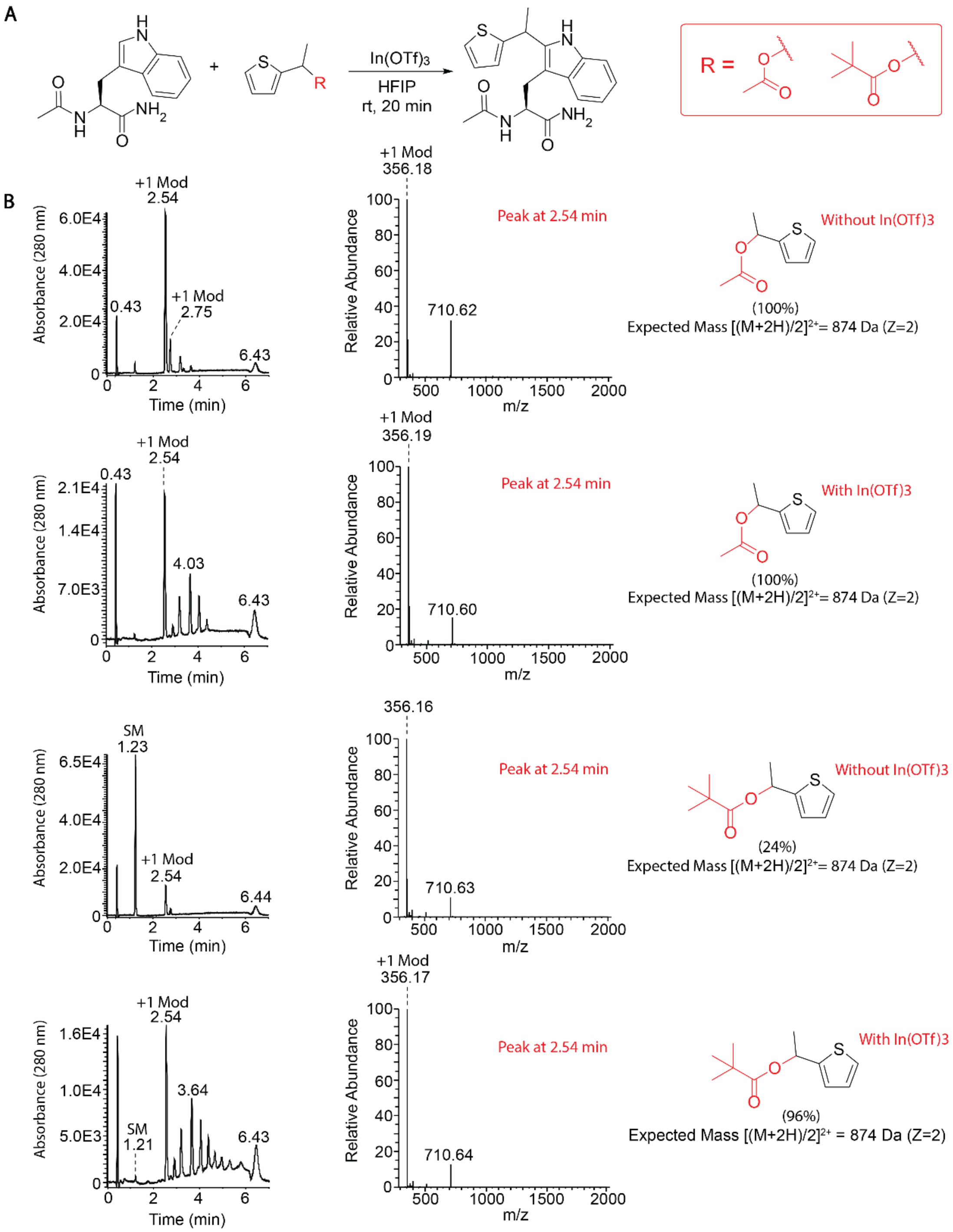

Figure S4. Liquid chromatography-mass spectrometry (LC-MS) analysis of modification of acetyl-tryptophan-amide with *O*-acetyl-thiophene-ethanol and *O*-pivaloyl-thiophene-ethanol in the presence and absence of indium triflate. Reaction conditions: acetyl-tryptophan-amide (0.1 mM), $In(OTf)_3$ (0 or 0.5 mM) and thiophene reagents (3 mM) in HFIP at rt for 30 min. (A) General reaction scheme. (B) LC-MS analysis: UV chromatograms are shown on the left, MS spectra of the starting material (SM) or the modification (+1 mod) are shown in the middle, and % conversion (in the parentheses) with the expected mass of a modified molecule is shown on the right.

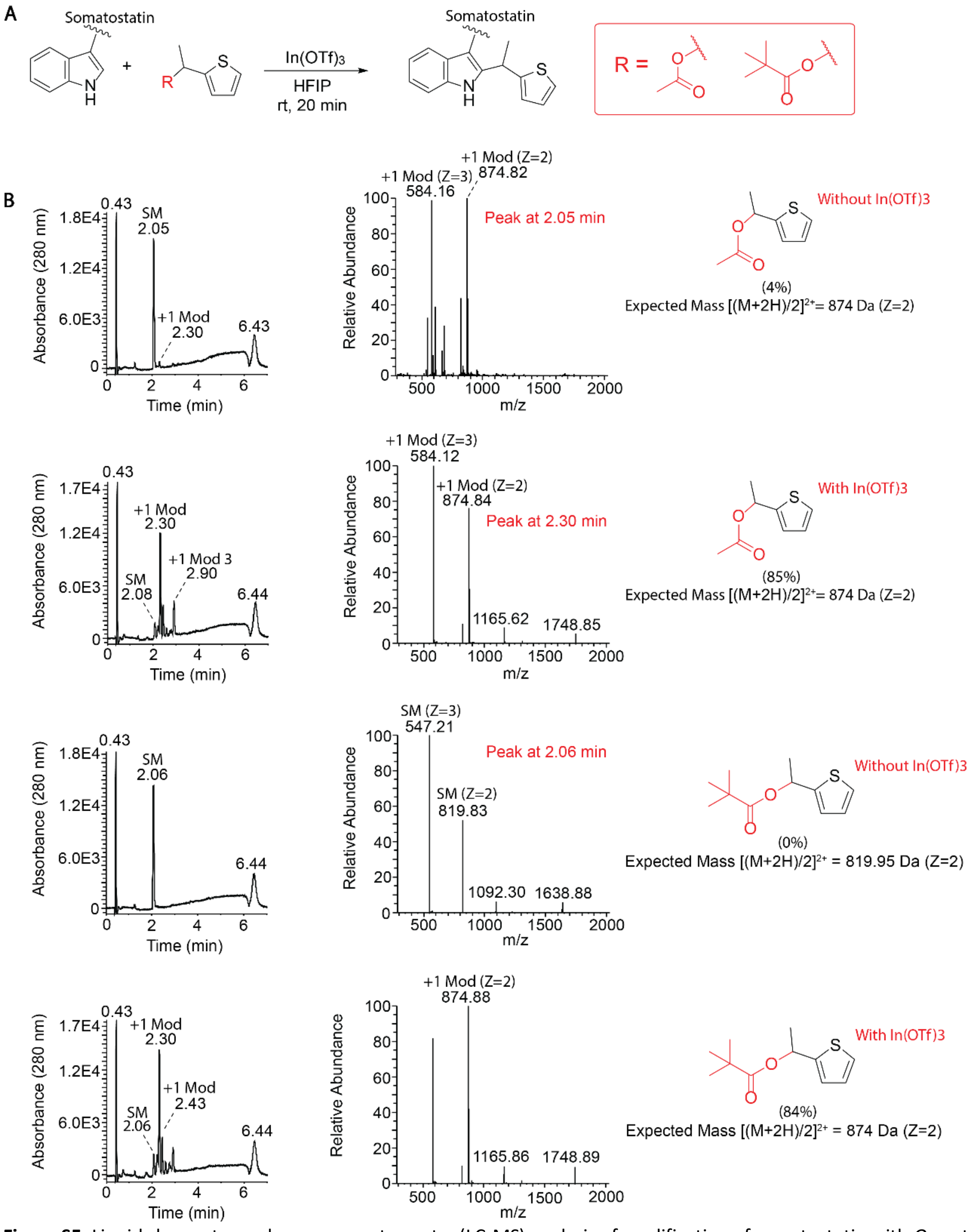

Figure S5. Liquid chromatography-mass spectrometry (LC-MS) analysis of modification of somatostatin with *O*-acetyl-thiophene-ethanol and *O*-pivaloyl-thiophene-ethanol in the presence and absence of indium triflate. Reaction conditions: Somatostatin (0.1 mM), $In(OTf)_3$ (0 or 0.5 mM), and thiophene reagents (3 mM) in HFIP at rt for 30 min. (A) General reaction scheme. (B) LC-MS analysis: UV chromatograms are shown on the left, MS spectra of the starting material (SM) or the modification (+1 mod) are shown in the middle, and % conversion (in the parentheses) with the expected mass of a modified molecule is shown on the right.

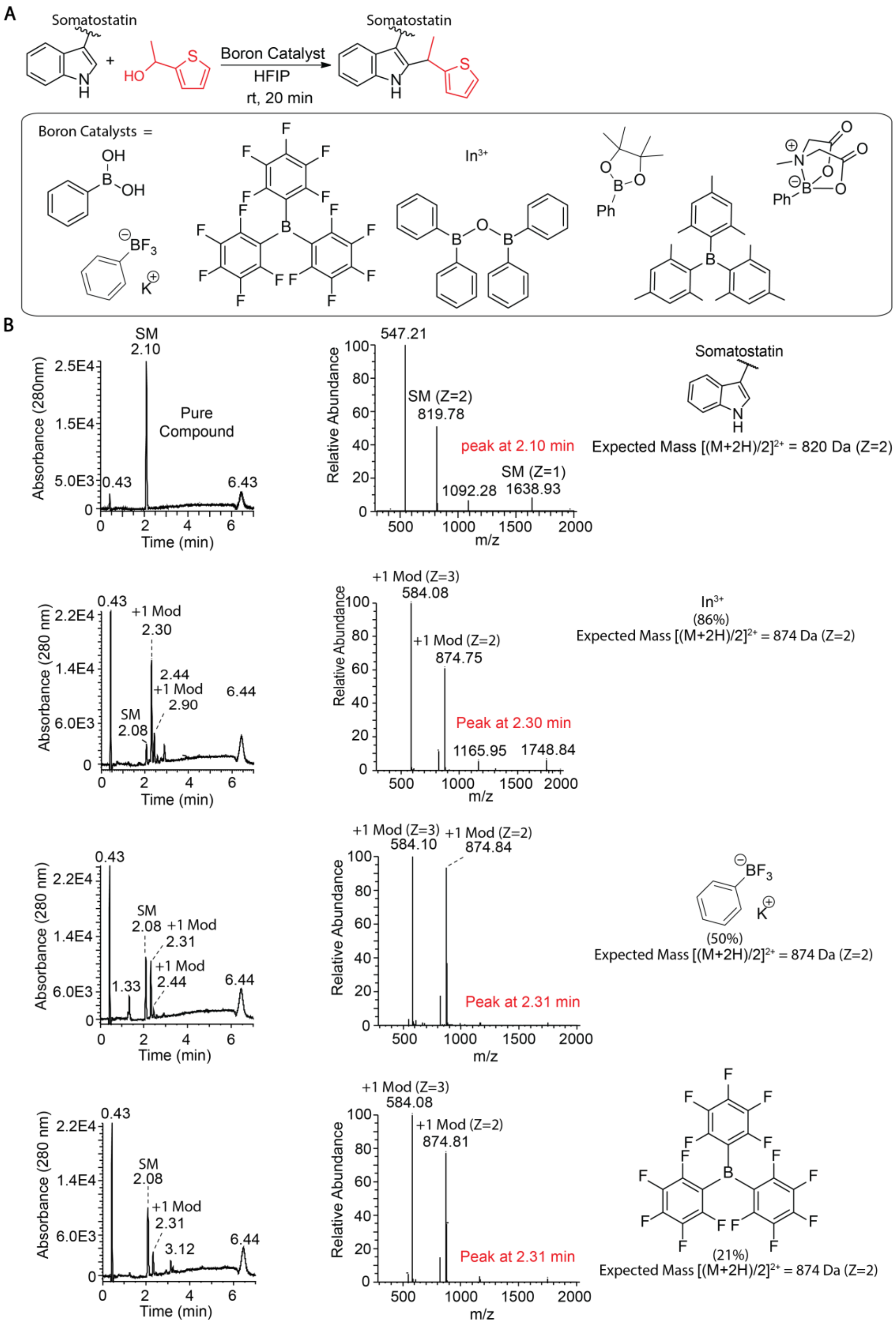
A
Somatostatin
HO
Boron Catalyst
HFIP
rt, 20 min
Somatostatin
Boron Catalysts =
In3+
B
SM
2.10
Pure
Compound
Absorbance (280nm)
Time (min)
Relative Abundance
547.21
SM (Z=2)
819.78
peak at 2.10 min
SM (Z=1)
1092.28
1638.93
m/z
Expected Mass [(M+2H)/2]2+ = 820 Da (Z=2)
+1 Mod
2.30
+1 Mod
2.90
SM
2.08
+1 Mod (Z=3)
584.08
+1 Mod (Z=2)
874.75
Peak at 2.30 min
1165.95
1748.84
In3+
(86%)
Expected Mass [(M+2H)/2]2+ = 874 Da (Z=2)
+1 Mod
2.31
+1 Mod
2.44
1.33
+1 Mod (Z=3)
584.10
+1 Mod (Z=2)
874.84
Peak at 2.31 min
(50%)
Expected Mass [(M+2H)/2]2+ = 874 Da (Z=2)
3.12
6.44
+1 Mod (Z=3)
584.08
+1 Mod (Z=2)
874.81
Peak at 2.31 min
(21%)
Expected Mass [(M+2H)/2]2+ = 874 Da (Z=2)

B. (Cont.)

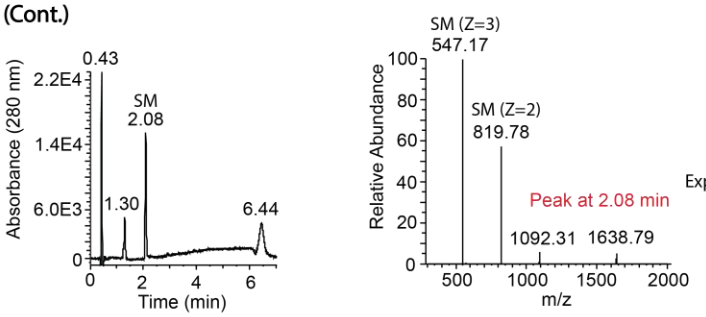

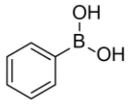

(0%)

Expected Mass [(M+2H)/2]$^{2+}$ = 820 Da (Z=2)

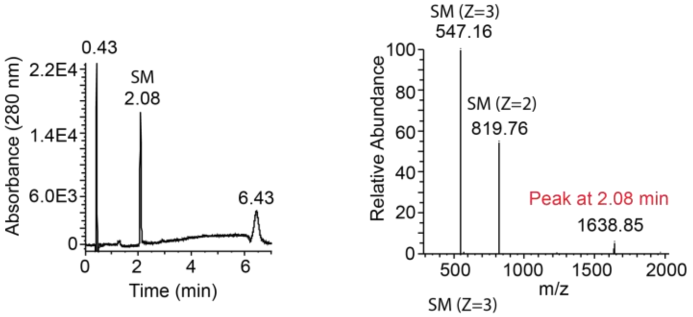

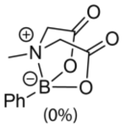

(0%)

Expected Mass [(M+2H)/2]$^{2+}$ = 820 Da (Z=2)

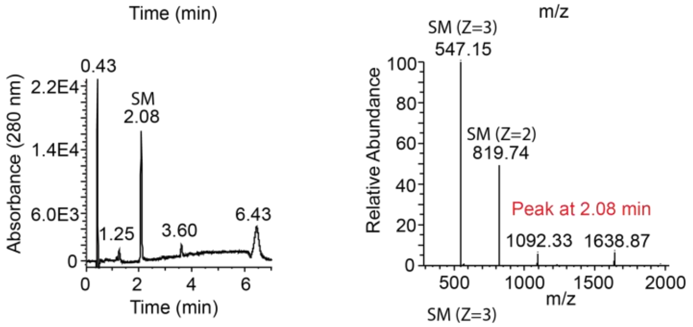

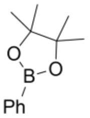

(0%)

Expected Mass [(M+2H)/2]$^{2+}$= 820 Da (Z=2)

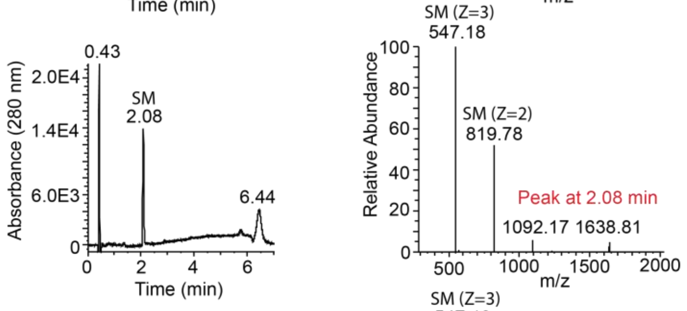

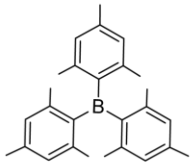

(0%)

Expected Mass [(M+2H)/2]$^{2+}$ = 820 Da (Z=2)

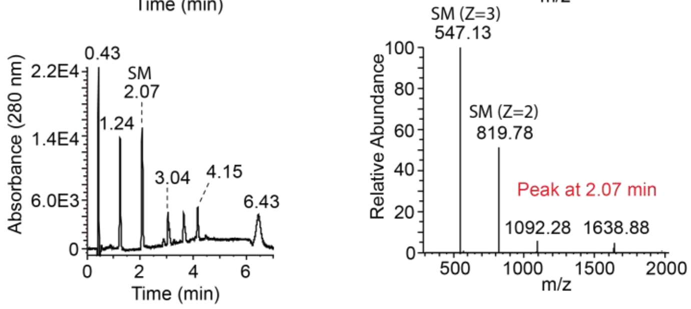

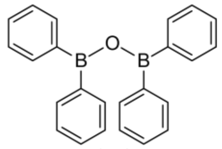

(0%)

Expected Mass [(M+2H)/2]$^{2+}$ = 820 Da (Z=2)

B. (Cont.)

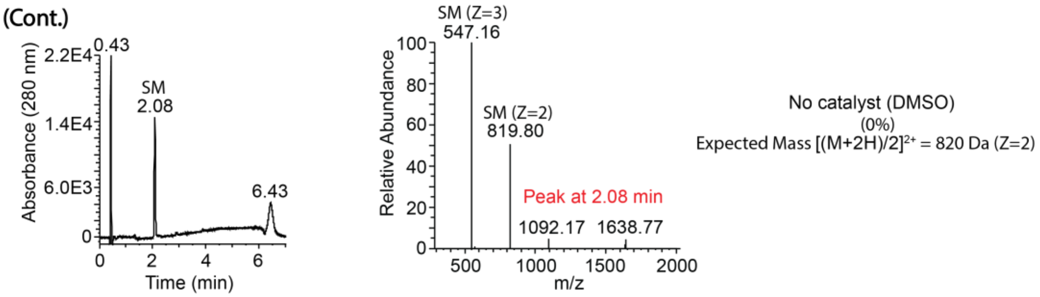

Figure S6. Liquid chromatography-mass spectrometry (LC-MS) analysis of modification of somatostatin with thiophene ethanol using different boron catalysts. Typical reaction conditions: somatostatin (0.1 mM), catalyst (0.5 mM) and thiophene-ethanol (3mM) in HFIP at rt for 30 min. (A) General reaction scheme. (B) LC-MS analysis: UV chromatograms are shown on the left, MS spectra of the starting material (SM) or the modification (+1 mod) are shown in the middle, and % conversion with the expected mass of a modified molecule is shown on the right.

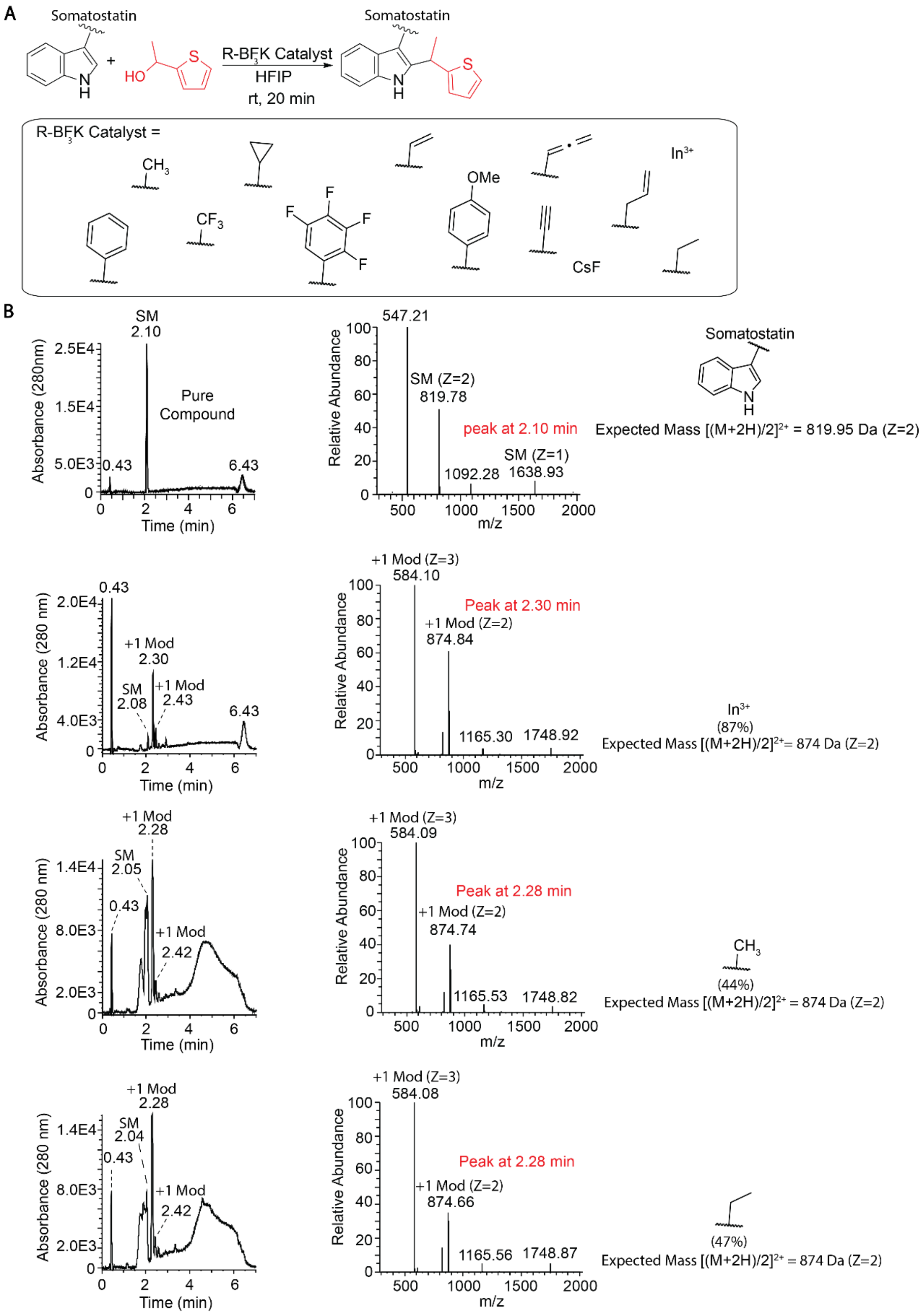
A
Somatostatin
HO
S
N
H
R-BF3K Catalyst
HFIP
rt, 20 min
R-BF3K Catalyst =
CH3
CF3
F
OMe
In3+
CsF
B
SM
2.10
Pure
Compound
0.43
6.43
Absorbance (280nm)
Time (min)
547.21
SM (Z=2)
819.78
peak at 2.10 min
SM (Z=1)
1092.28
1638.93
Relative Abundance
m/z
Expected Mass [(M+2H)/2]2+ = 819.95 Da (Z=2)
+1 Mod (Z=3)
584.10
Peak at 2.30 min
+1 Mod (Z=2)
874.84
1165.30
1748.92
+1 Mod
2.30
SM
2.08
+1 Mod
2.43
Absorbance (280 nm)
(87%)
Expected Mass [(M+2H)/2]2+= 874 Da (Z=2)
+1 Mod
2.28
SM
2.05
+1 Mod
2.42
+1 Mod (Z=3)
584.09
Peak at 2.28 min
+1 Mod (Z=2)
874.74
1165.53
1748.82
(44%)
Expected Mass [(M+2H)/2]2+ = 874 Da (Z=2)
SM
2.04
+1 Mod (Z=3)
584.08
+1 Mod (Z=2)
874.66
1165.56
1748.87
(47%)

B. (Cont.)

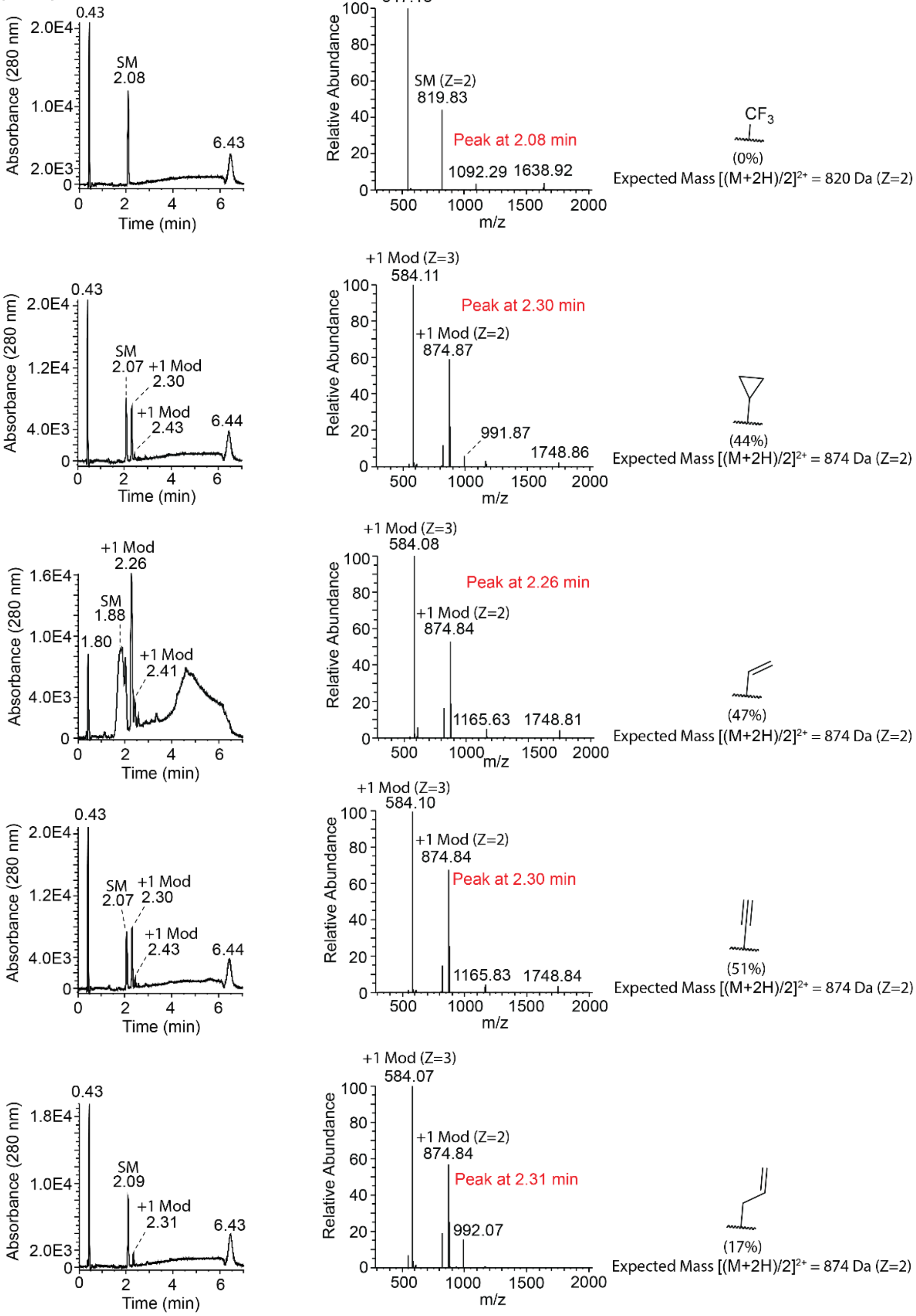

Absorbance (280 nm)
Time (min)
Relative Abundance
m/z
0.43
SM 2.08
6.43
SM (Z=3) 547.18
SM (Z=2) 819.83
Peak at 2.08 min
1092.29
1638.92
CF3
(0%)
Expected Mass [(M+2H)/2]2+ = 820 Da (Z=2)
SM 2.07
+1 Mod 2.30
+1 Mod 2.43
6.44
+1 Mod (Z=3) 584.11
Peak at 2.30 min
+1 Mod (Z=2) 874.87
991.87
1748.86
(44%)
Expected Mass [(M+2H)/2]2+ = 874 Da (Z=2)
+1 Mod 2.26
SM 1.88
1.80
+1 Mod 2.41
+1 Mod (Z=3) 584.08
Peak at 2.26 min
+1 Mod (Z=2) 874.84
1165.63
1748.81
(47%)
Expected Mass [(M+2H)/2]2+ = 874 Da (Z=2)
SM 2.07
+1 Mod 2.30
+1 Mod 2.43
6.44
+1 Mod (Z=3) 584.10
+1 Mod (Z=2) 874.84
Peak at 2.30 min
1165.83
1748.84
(51%)
Expected Mass [(M+2H)/2]2+ = 874 Da (Z=2)
SM 2.09
+1 Mod 2.31
6.43
+1 Mod (Z=3) 584.07
+1 Mod (Z=2) 874.84
Peak at 2.31 min
992.07
(17%)
Expected Mass [(M+2H)/2]2+ = 874 Da (Z=2)

B. (Cont.)

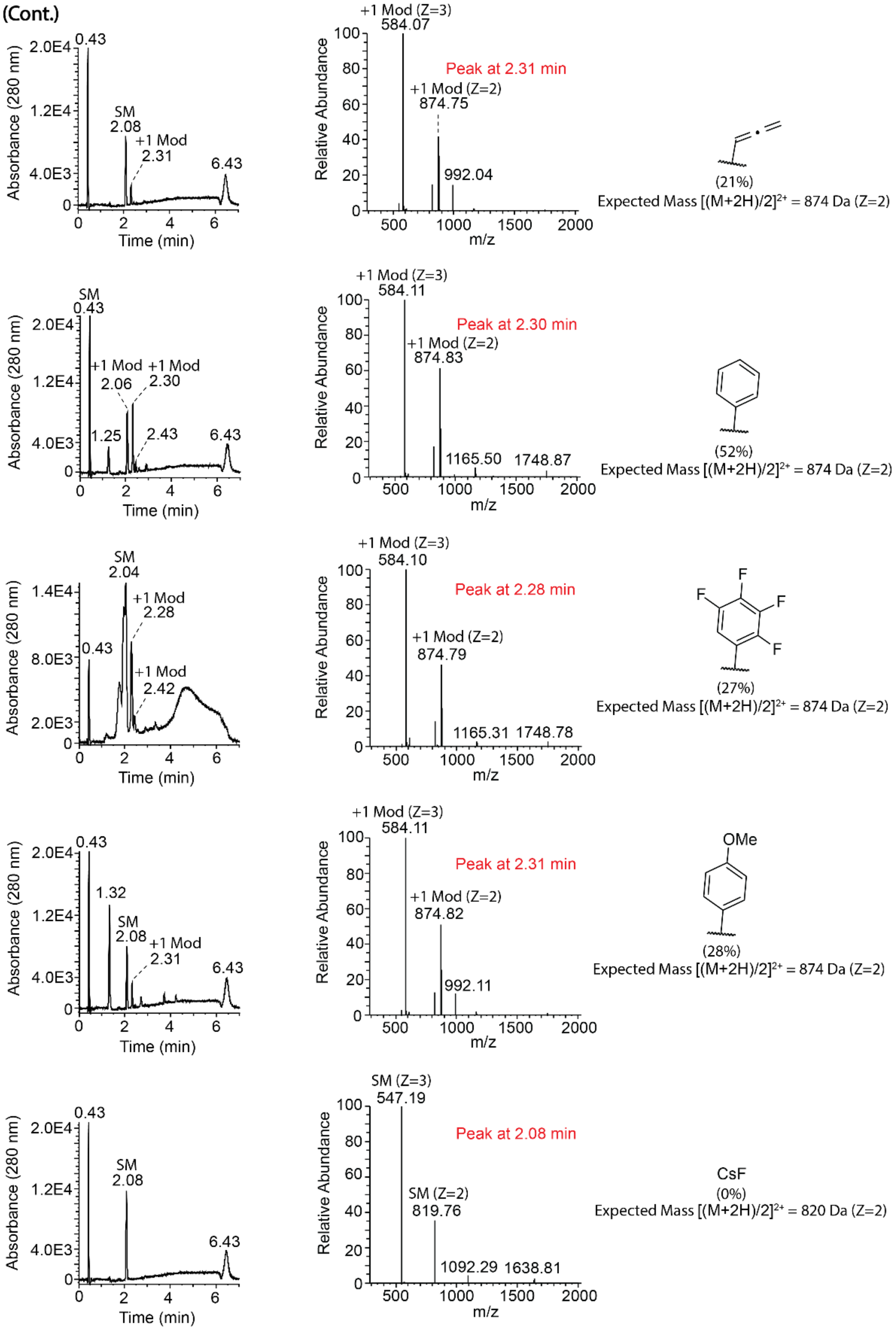

Figure S7. Liquid chromatography-mass spectrometry (LC-MS) analysis of modification of somatostatin with thiophene-ethanol using potassium alkyl-trifluoroborate (RBF_3K) catalysts. Typical reaction conditions: somatostatin (0.1 mM), catalyst (0.5 mM) and thiophene-ethanol (3mM) in HFIP at rt for 30 min. (A) General reaction scheme. (B) LC-MS analysis: UV chromatograms are shown on the left, MS spectra of the starting material (SM) or the modification (+1 mod) are shown in the middle, and % conversion with the expected mass of a modified molecule is shown on the right.

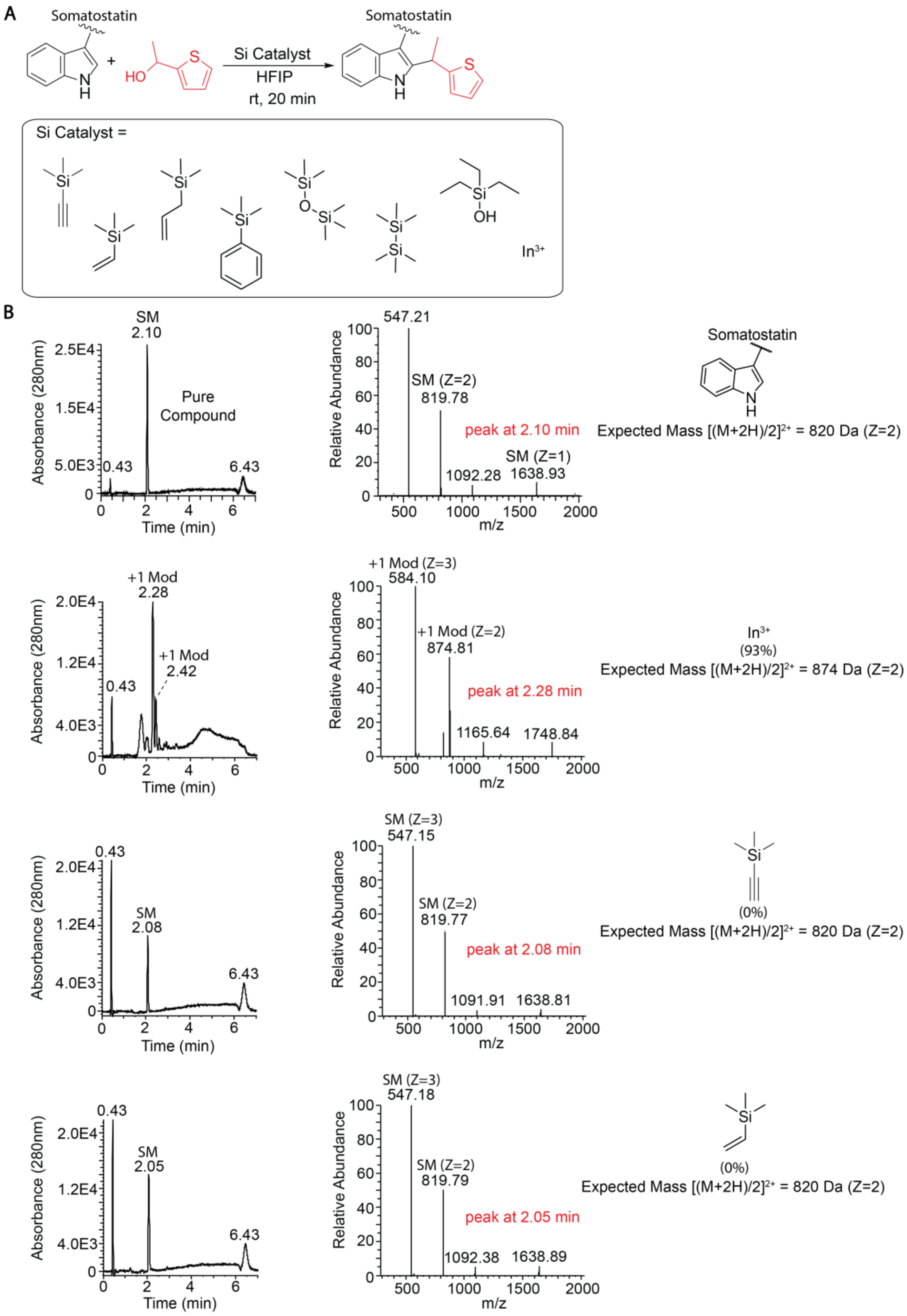
A
Somatostatin
HO
S
N
H
Si Catalyst
HFIP
rt, 20 min
Somatostatin
Si Catalyst =
Si
O
OH
In3+
B
SM
2.10
Pure
Compound
0.43
6.43
Absorbance (280nm)
2.5E4
1.5E4
5.0E3
Time (min)
547.21
SM (Z=2)
819.78
peak at 2.10 min
SM (Z=1)
1638.93
1092.28
Relative Abundance
m/z
Expected Mass [(M+2H)/2]2+ = 820 Da (Z=2)
+1 Mod
2.28
+1 Mod
2.42
2.0E4
1.2E4
4.0E3
+1 Mod (Z=3)
584.10
+1 Mod (Z=2)
874.81
peak at 2.28 min
1165.64
1748.84
In3+
(93%)
Expected Mass [(M+2H)/2]2+ = 874 Da (Z=2)
SM
2.08
SM (Z=3)
547.15
SM (Z=2)
819.77
peak at 2.08 min
1091.91
1638.81
(0%)
Expected Mass [(M+2H)/2]2+ = 820 Da (Z=2)
SM
2.05
SM (Z=3)
547.18
SM (Z=2)
819.79
peak at 2.05 min
1092.38
1638.89
(0%)
Expected Mass [(M+2H)/2]2+ = 820 Da (Z=2)

B. (Cont.)

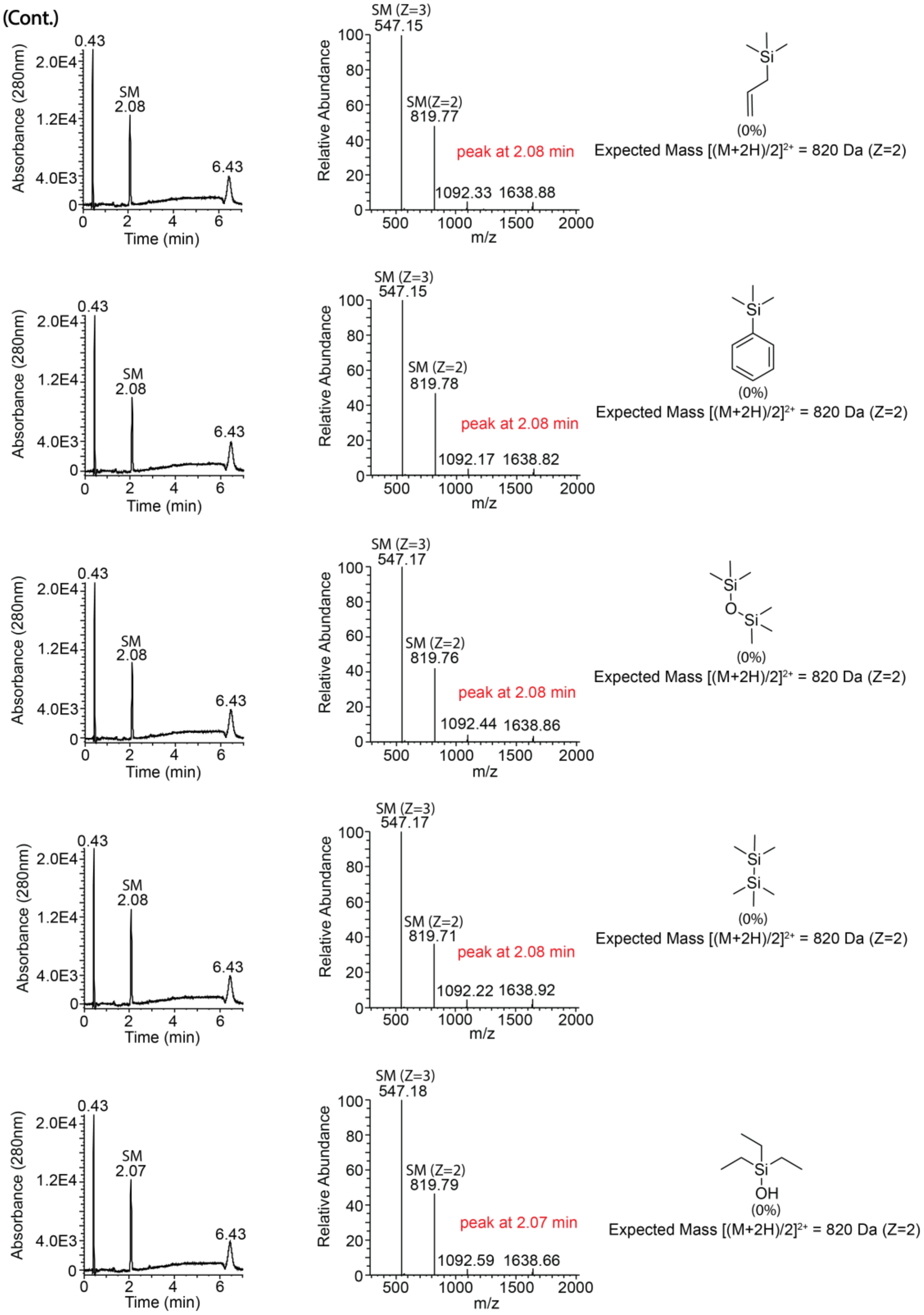

Figure S8. Liquid chromatography-mass spectrometry (LC-MS) analysis of modification of somatostatin with thiophene ethanol using different silicon catalysts. Typical reaction conditions: Somatostatin (0.1 mM), silicon catalyst (0.5 mM) and thiophene-ethanol (3mM) in HFIP at rt for 30 min. (A) General reaction scheme. (B) LC-MS analysis: UV chromatograms are shown on the left, MS spectra of the starting material (SM) or the modification (+1 mod) are shown in the middle, and % conversion with the expected mass of a modified molecule is shown on the right.

A

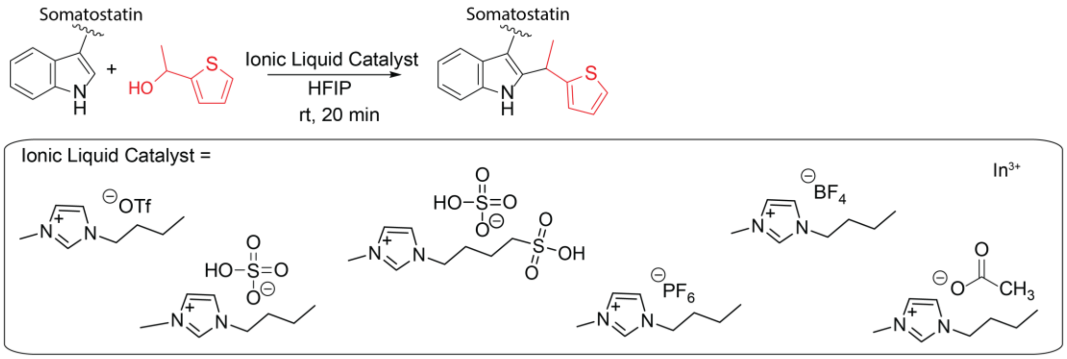

Somatostatin
HO
S
Ionic Liquid Catalyst
HFIP
rt, 20 min
Somatostatin
Ionic Liquid Catalyst =
OTf
HO-S=O
BF4
In3+
PF6
CH3

B

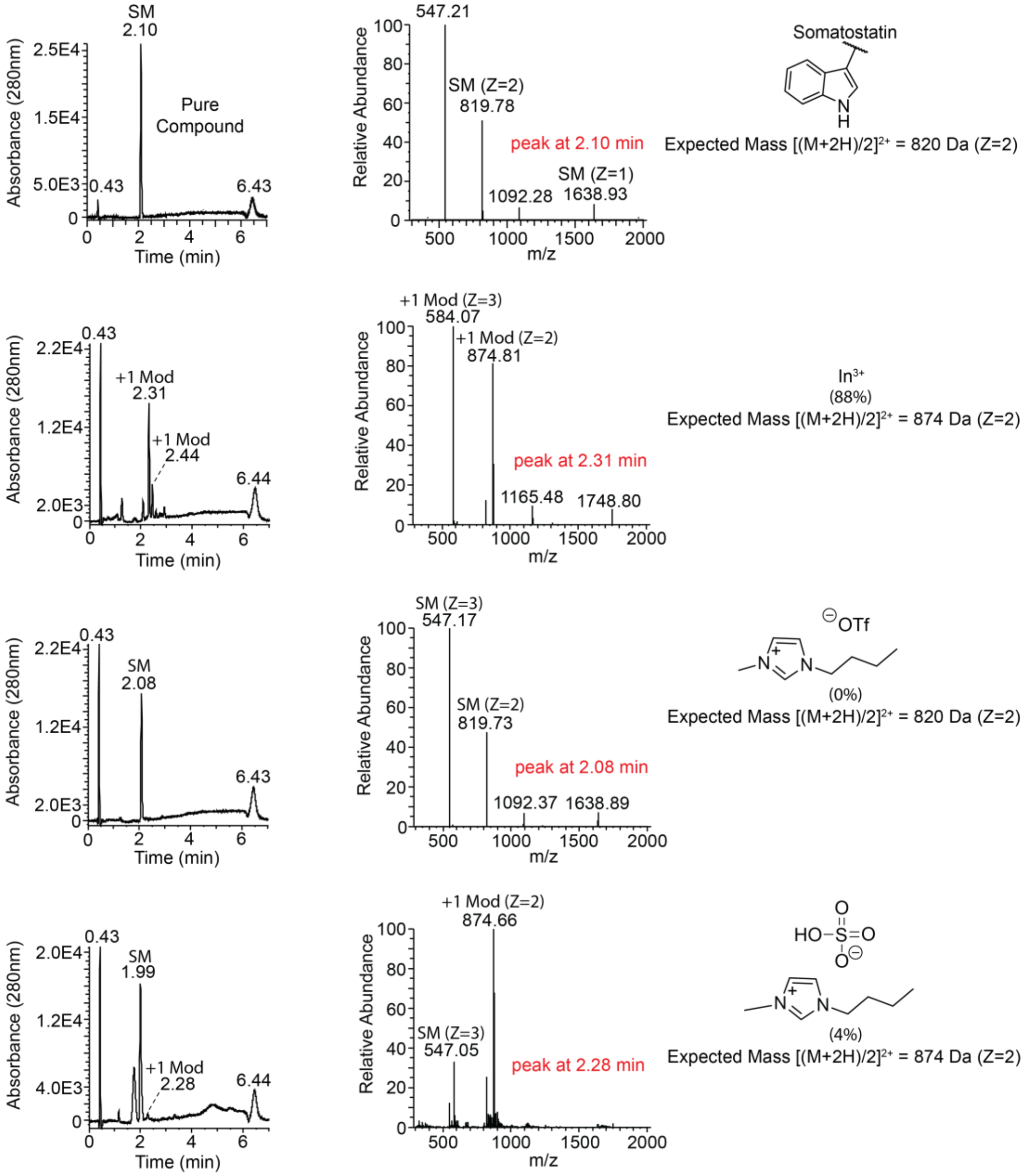

SM 2.10
Pure Compound
Absorbance (280nm)
Time (min)
0.43
6.43
547.21
SM (Z=2) 819.78
peak at 2.10 min
SM (Z=1) 1638.93
1092.28
Relative Abundance
m/z
Somatostatin
Expected Mass [(M+2H)/2]2+ = 820 Da (Z=2)
+1 Mod 2.31
+1 Mod 2.44
6.44
+1 Mod (Z=3) 584.07
+1 Mod (Z=2) 874.81
peak at 2.31 min
1165.48
1748.80
In3+
(88%)
Expected Mass [(M+2H)/2]2+ = 874 Da (Z=2)
SM 2.08
SM (Z=3) 547.17
SM (Z=2) 819.73
peak at 2.08 min
1092.37
1638.89
OTf
(0%)
Expected Mass [(M+2H)/2]2+ = 820 Da (Z=2)
SM 1.99
+1 Mod 2.28
+1 Mod (Z=2) 874.66
SM (Z=3) 547.05
peak at 2.28 min
HO-S=O
(4%)
Expected Mass [(M+2H)/2]2+ = 874 Da (Z=2)

B. (Cont.)

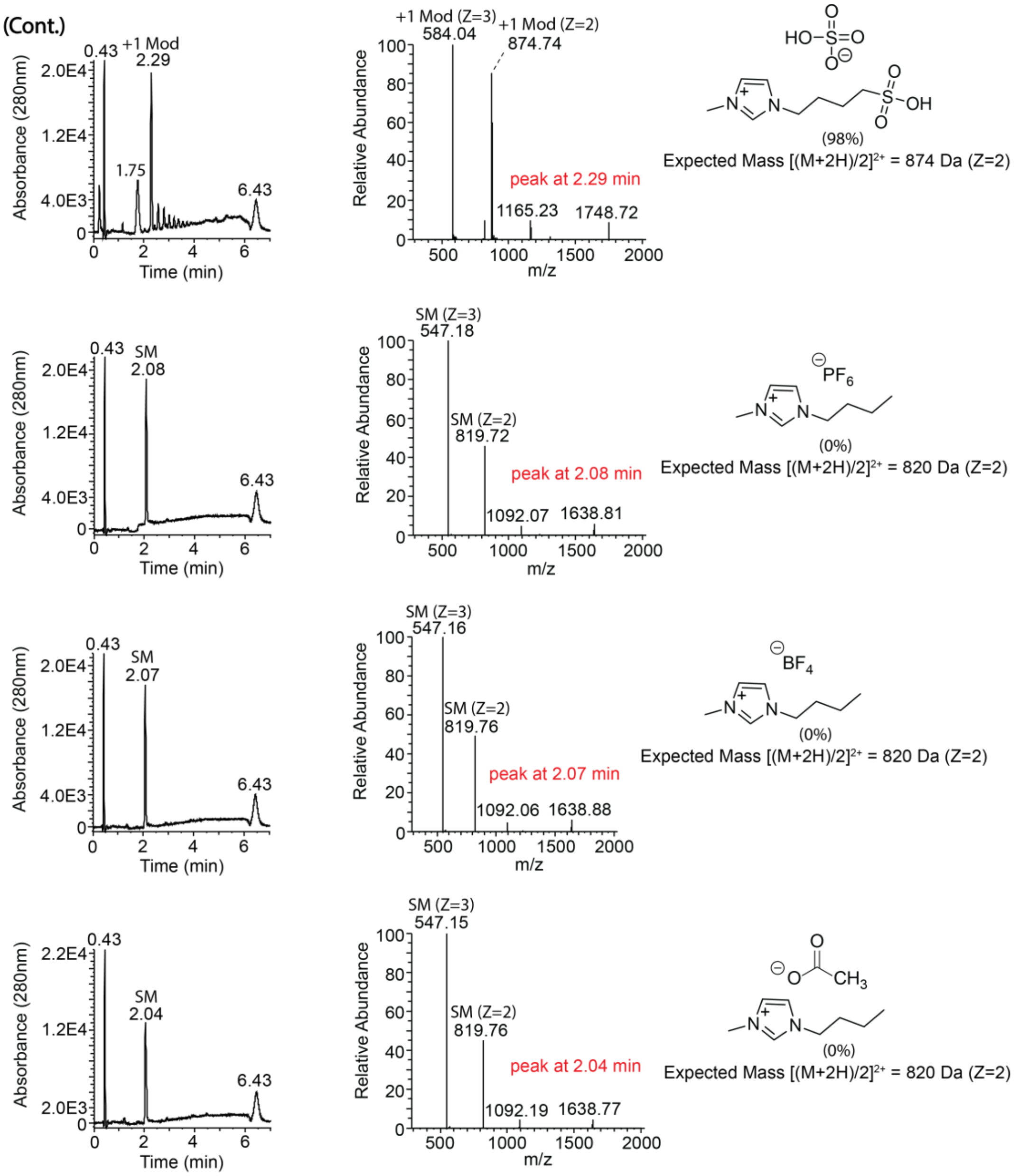

Figure S9. Liquid chromatography-mass spectrometry (LC-MS) analysis of modification of somatostatin with thiophene ethanol using different ionic acid catalysts. Typical reaction conditions: Somatostatin (0.1 mM), catalyst (0.5 mM) and thiophene-ethanol (3mM) in HFIP at rt for 30 min. (A) General reaction scheme. (B) LC-MS analysis: UV chromatograms are shown on the left, MS spectra of the starting material (SM) or the modification (+1 mod) are shown in the middle, and % conversion with the expected mass of a modified molecule is shown on the right.

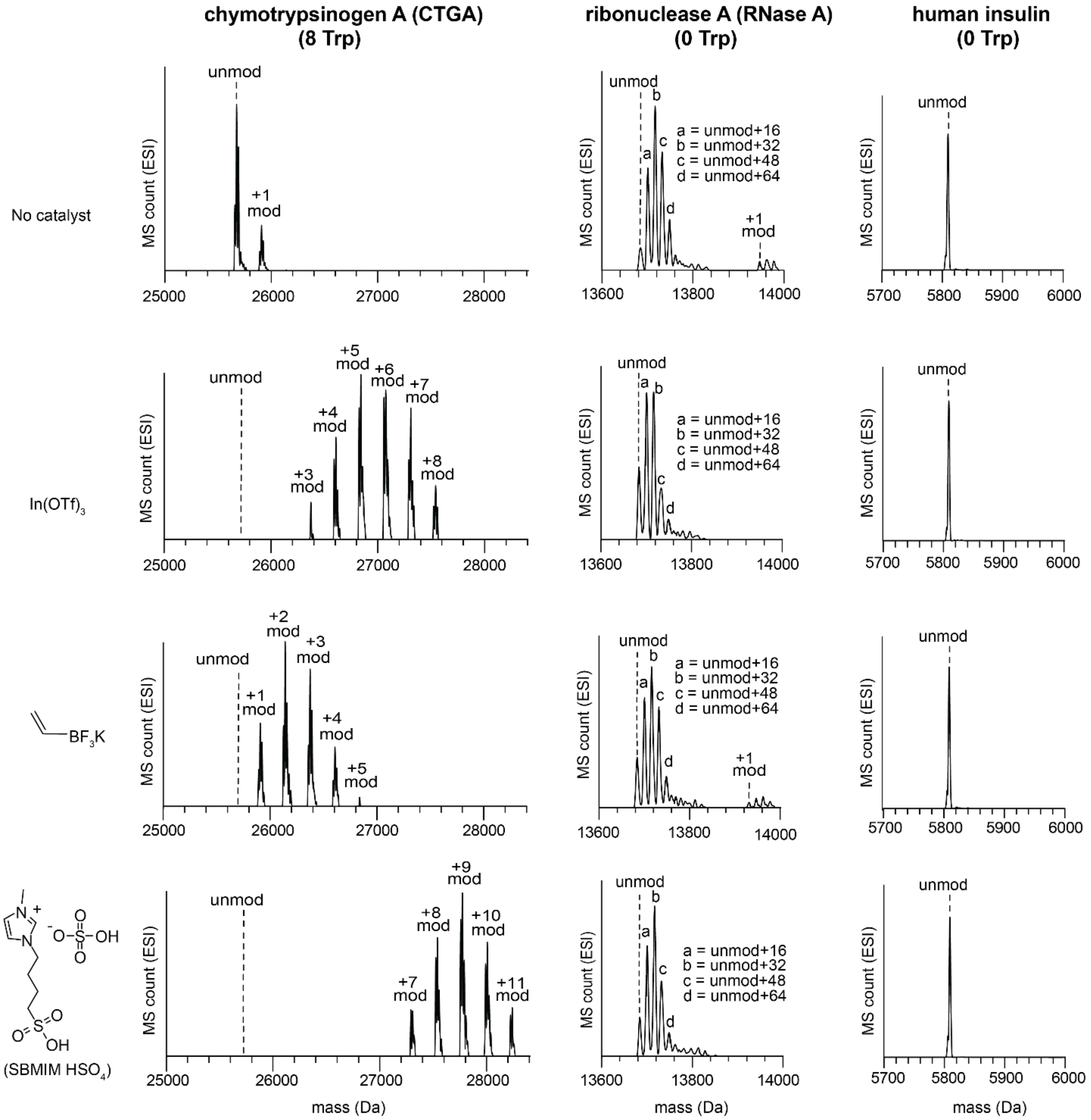

Figure S10. LCMS analysis of modification of peptide and protein by alkyne-tagged thiophene (**1k**). Modification conditions: protein/peptide (CTGA: 25 µM, RNase: 25 µM, and insulin: 10 µM), catalyst (0.5 mM), and alkyne-tagged thiophene reagent (3 mM) in hexafluoroisopropanol (HFIP) at room temperature for 20 minutes. Mod: modified peptide/protein. Unmod: unmodified peptide/protein.

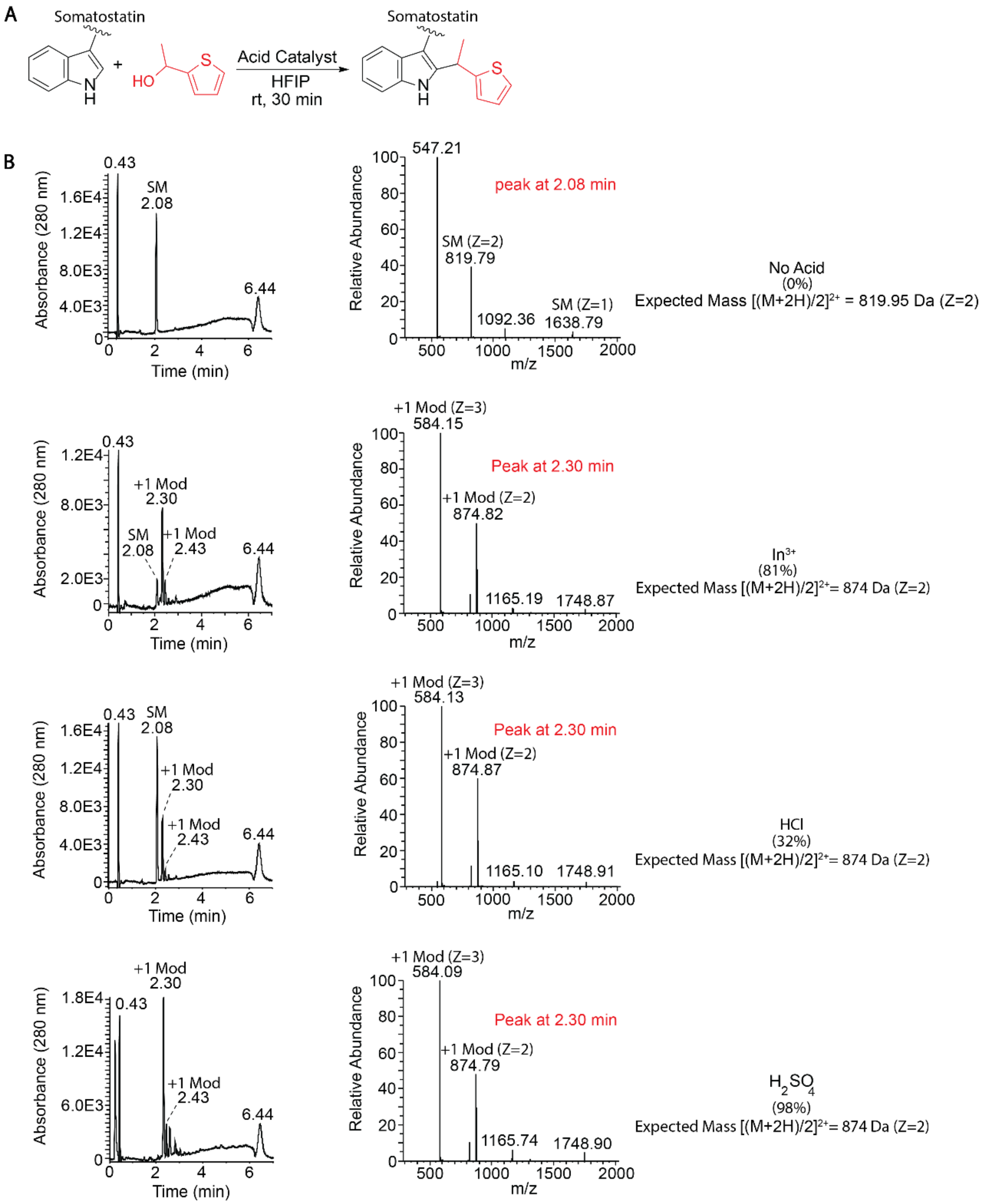

Figure S11. Liquid chromatography-mass spectrometry (LC-MS) analysis of modification of somatostatin with thiophene-ethanol using hydrochloric and sulfuric acid. Reaction conditions: Somatostatin (0.1 mM), catalyst (0.5 mM) and thiophene-ethanol (3 mM) in HFIP at rt for 30 min. (A) General reaction scheme. (B) LC-MS analysis: UV chromatograms are shown on the left, MS spectra of the starting material (SM) or the modification (+1 mod) are shown in the middle, and % conversion (in the parentheses) with the expected mass of a modified molecule is shown on the right.

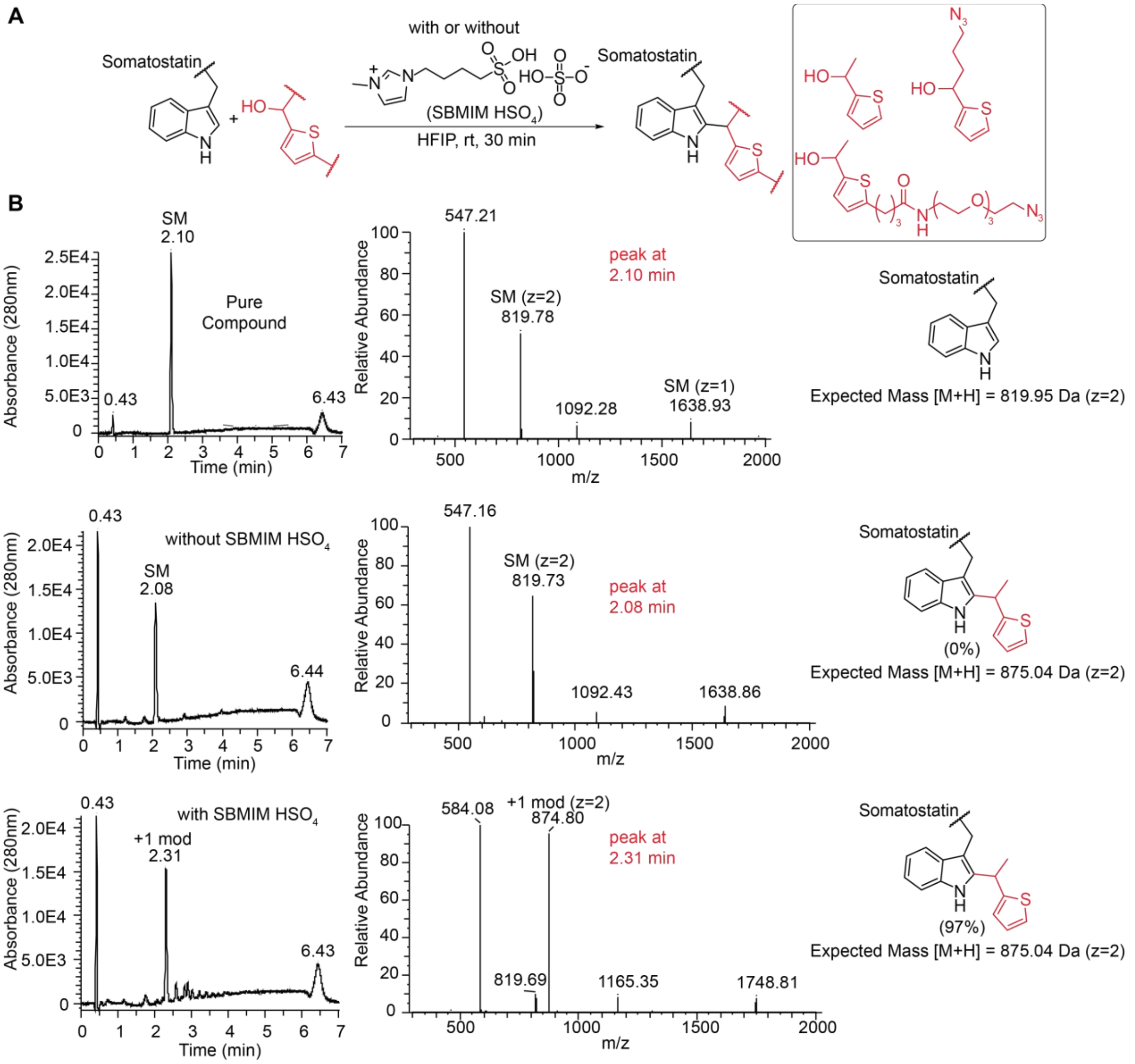
A
Somatostatin
with or without
(SBMIM HSO4)
HFIP, rt, 30 min
Somatostatin
B
SM
2.10
Pure
Compound
0.43
6.43
Absorbance (280nm)
Time (min)
547.21
SM (z=2)
819.78
1092.28
SM (z=1)
1638.93
peak at
2.10 min
Relative Abundance
m/z
Somatostatin
Expected Mass [M+H] = 819.95 Da (z=2)
0.43
without SBMIM HSO4
SM
2.08
6.44
547.16
SM (z=2)
819.73
peak at
2.08 min
1092.43
1638.86
Somatostatin
(0%)
Expected Mass [M+H] = 875.04 Da (z=2)
0.43
with SBMIM HSO4
+1 mod
2.31
6.43
584.08
+1 mod (z=2)
874.80
peak at
2.31 min
819.69
1165.35
1748.81
Somatostatin
(97%)
Expected Mass [M+H] = 875.04 Da (z=2)

B (continued)

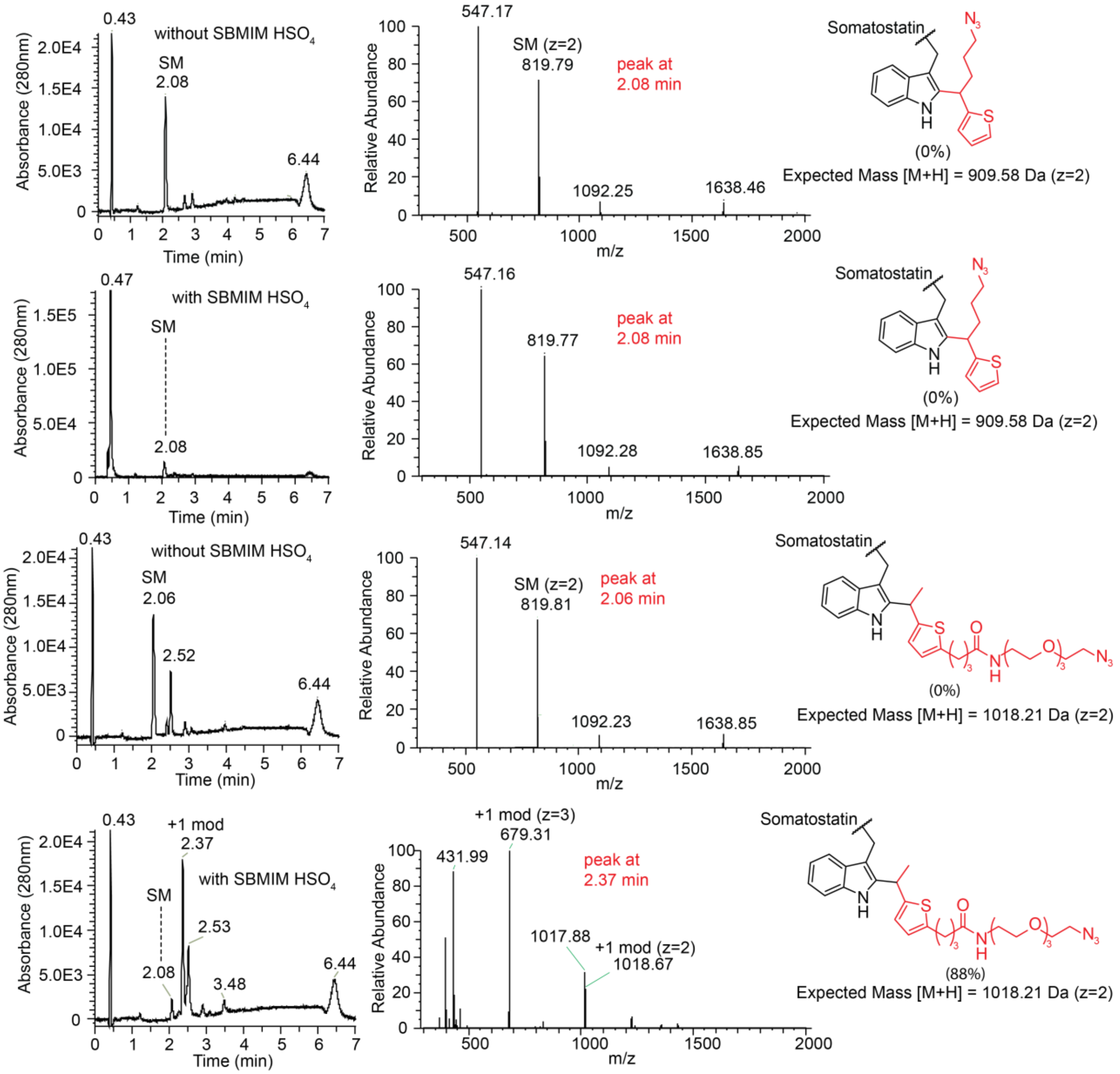

Figure S12. Liquid chromatography-mass spectrometry (LC-MS) analysis of modification of somatostatin with azide containing thiophene reagents. Typical reaction conditions: Somatostatin (0.1 mM), SBMIM HSO_4 (0.5 mM) and thiophene-ethanol (3mM) in HFIP at rt for 30 min. (A) General reaction scheme. (B) LC-MS analysis: UV chromatograms of the pure somatostatin or the reaction mixtures are shown on the left, MS spectra of starting material (SM) or the modification (+1 mod) are shown on the middle, and % conversion with expected mass of modified molecule is shown on the right.

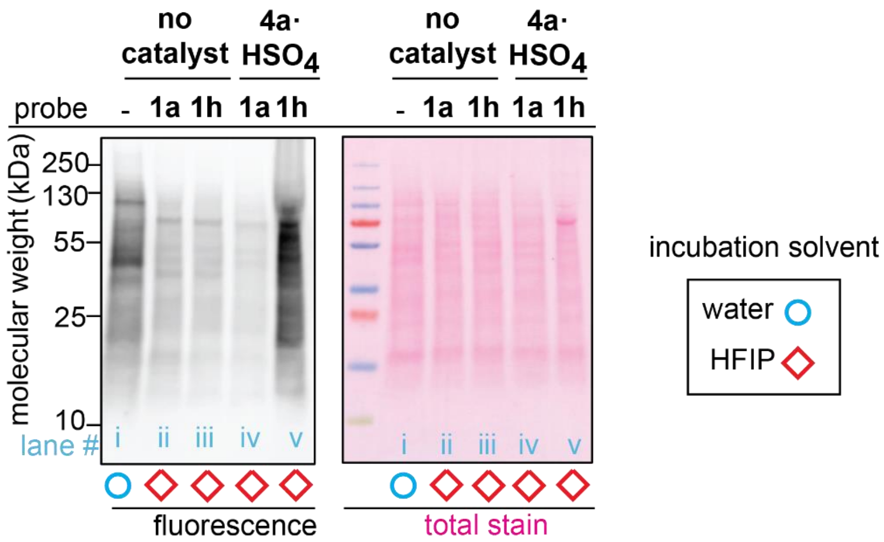

Figure S13. Technical replicate of anti-biotin western blot experiment of the cell lysate shown in Fig.5C.

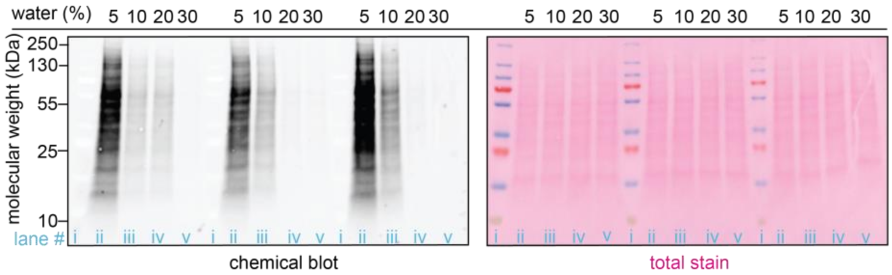

Figure S14. Chemical blot analysis of HEK293T cell lysate labeled with **1k** in HFIP at different concentrations of water in the reaction. Experiments are triplicated and labeled shown in lane (ii), (iii), (iv), and (v) on the left, middle, and right sides of the membrane. Water concentrations on lanes (ii), (iii), (iv), and (v) are 5%, 10%, 20%, and 30%. Reaction conditions: cell lysate (0.1375 mg/mL. 5% water was derived from the cell lysate stock.), **4a·HSO$_4$** (0.5 mM), and thiophene reagent **1k** (3 mM) in HFIP/water at room temperature for 5 min. Chemical-blotting conditions for detection of the alkyne handle on a blot membrane: sodium ascorbate (1.5 mM), THPTA (0.1 mM), 5, (0.015 mM), and $CuSO_4$ (0.075 mM) in a 1:1 DMSO/H_2O solution for 30 min.

General information

Materials and reagents

Unless stated otherwise, all chemicals were purchased from commercial vendors with their catalog I.D. listed as well. All the chemicals synthesized were done so in air unless stated otherwise. Acetyl-tryptophan-amide (#A6501), DBCO-biotin (#760749), and Propargyl-NHS (#764221-50MG) were purchased from Sigma-Aldrich. Acetyl-tryptophan-amide (#4002401) and Somatostatin (#H-1490.0005) were also purchased from Bachem. Somatostatin was also purchased from MedChem Express (#HY-P004/CS-5743). Vinyltrifluoroborate (#P14791G) and N-Propargylmaeimide (#TCP2139- 25MG) were purchased from TCI. N-Propargylmaeimide (#A932884-250mg) and sulfonic acid ionic liquid (#A703493) were purchased from Ambeed. Hexafluoroisopropanol (HFIP) was purchased from Chem-Impex (#00040) and Apollo Scientific (#PC4750). Protein gel/blot molecular weight marker was purchased from Thermo Scientific (#26619). Coumarin azide was purchased from Carbosynth (#FA31762). Thiophene-ethanol,[2] potassium allenyltrifluroborate,[3] and alkyne-tagged thiophene[1] were synthesized according to reported procedures.

Cell culture

HEK293T cells (ATCC, CRL-3216) were cultured in Dulbecco's Modified Eagle Medium (DMEM) with Glutamax, 10% fetal bovine serum, and 1% penicillin-streptomycin. HEK293T cells cultured without poly-D-lysine coating at 100% confluency (15 million) were washed three times with PBS, separated from the buffer by centrifugation (1000 rcf, 3 min, 4 °C), placed in –80 °C for 30 min, and lysed in PBS buffer (960 µL) containing 0.1% SDS, 0.1% triton and EDTA-free protease inhibitor (complete tablets, Roche #04-693-159-001). The cell lysate was transferred to 1.7-mL Eppendorf tube, and a homogenizer was used to assure the complete lysis of cells. The cell lysate was placed for 30 min in ice before centrifugation (15000 rcf, 15min, 4°C), and the supernatant was used as a stock solution for the labeling experiment. The concentration of the lysate stock was determined by Bradford assay.

Instrumentation

NMR

NMR was conducted on Bruker AVANCE NEO 500, 600, and 700.

LCMS

Analysis of the peptides was conducted on a Thermo Vanquis LC System and LTQ-XL linear ion trap MS software. A C18 Reverse-phase column (Hypersil Gold 25003-032130 model, with a particle size of 3µm, and with diameters of 2.1 mm and 30 mm (length)) was used at a wavelength of 280 nm for UV detection at a rate of 0.4 mL/min with a gradient of acetonitrile (10-90% for about 3.5 min and then 90% for 1.5 min) composed of 0.1% formic acid in a 0.1% formic acid aqueous solution. A phenyl reverse-phase column (MAbPac 088648 model, with a particle size of 4 µm, and with dimensions of 2.1 mm and 50 mm (length)) was conducted by positive MS ion detection at a rate of 0.2 mL/min with a gradient of acetonitrile (10-90% for about 3.5 min and then 90% for 1.5 min) composed of 0.1% formic acid in a 0.1% formic acid aqueous solution.
Conversion for modification were calculated by dividing the peak area of the mod by the peak of the mod with the starting material.

FT-IR

IR was conducted in Cary 630 FTIR spectrometer by Agilent Technologies.

Gel and Blott Imaging

Gel and blotting were both employed under Amersham ImageQuant 800 (Cytiva) under 360-nm, 535-nm, and 635-nm light sources with their matching emission bandpass filters at 525 nm (±20 nm), 605 nm (±40 nm), and 705 nm (±40 nm).

Experimental procedures

General procedure for acetyl-tryptophan-amide or tryptophan-containing peptides

HFIP (typically around 97-98 µL) was added to an Eppendorf tube (1.7 mL), and then acetyl-tryptophan-amide or tryptophan-containing compounds, thiophene-ethanol derivatives, and catalyst were added. Between the addition of each substrate/reagent/catalyst, the Eppendorf tube was capped to prevent evaporation of HFIP. The mixture was incubated at rt for 5–60 min,diluted in a 4:1 H_2O/MeOH mixture and centrifuged at 15,000 rpm for 15 min, and submitted to LCMS.

<u>Typical acetyl-tryptophan-amide reaction concentrations</u>

Acetyl-tryptophan-amide (0.3-mM final concentration from 50-mM stock in DMSO), catalyst (0.3-mM final concentration from a 25-mM stock of a 95:5 HFIP/DMSO mixture), and thiophene-ethanol derivatives (1.2-mM final concentration from 75-mM stock solution in in 1,4-dioxane) in HFIP at rt for 20 min.

<u>Typical peptide reaction concentrations:</u>

Tryptophan-containing peptide (0.1-mM final concentration. from 5-mM stock in water), catalyst (0.5-mM final concentration from 100-mM stock in DMSO), and thiophene-ethanol derivative (3-mM final concentration from 75-mM stock solution in 1,4-dioxane) in HFIP rt for 20 min.

Modification of cell lysate

In an Eppendorf tube, HFIP (typically 20 µL), catalyst (0.5-mM final concentration from 12.5-mM stock of a 95:5 HFIP/DMSO mixture), cell lysate (0.14-mg/mL final concentration from 2.75-mg/mL stock solution in a lysis buffer. The final volume of water during the tryptophan labeling is kept below 5% v/v), and thiophene reagent (3-mM final concentration from 75 mM stock in 1,4-dioxane) were mixed, and the mixture was incubated at rt for 30 min. An equal volume (typically 50 µL) of 1X loading buffer (LDS buffer prepared from 4X loading buffer, Thermo BN2003 and water) was added, and HFIP was removed by a gentle flow of nitrogen gas for 3 min, confirmed by the volume decrease upon the evaporation process. The sample volumes were adjusted to the original volume of the aqueous solution (typically 50 µL) by addition of water when necessary. Freshly prepared stock of dithiothreitol or DTT (20-mM final concentration from 1-M stock in water) was added. The samples were then heated at 95 °C for 5 min (afterward, the sample volumes were adjusted to the original volume of the aqueous solution by addition of water when necessary), subjected to SDS-PAGE at 140V for 45 min, and transferred to a PVDF membrane through a semi-dry transfer process. The membrane was subjected to either chemical blot (alkyne detection) or western blot (biotin detection) following the procedures below

The lysine- and cysteine-targeting modification in aqueous solutions were performed in 5-mM N-methylmorpholine buffer (pH 7) by addition of cell lysate (0.14-mg/mL final concentration from 2.75 mg/mL stock solution in a lysis buffer) and NHS ester (0.5-mM final concentration from 16.6-mM stock in DMSO) or maleimide (3-mM final concentration from 100-mM stock in DMSO). To 20 µL of the reaction mixture, 20 µL of 2X loading buffer containing DTT (40 mM) was added. The reaction mixture was heated at at 95 °C for 5 min (afterward, the sample volumes were adjusted to the original volume of the aqueous solution by addition of water when necessary) and subjected to the SDS-PAGE and transfer processes in the same way as the HFIP modification samples.

Chemical blotting for detection of alkyne handles on cell lysate

The blot membrane after the transfer process was rinsed with methanol twice and then water twice. Chemical blotting was performed by addition of 19.7 mL of 1:1 H_2O DMSO mixture, 300 µL of freshly prepared sodium ascorbate (1.5-mM final concentration from 100-mM stock in water), 20 µL of THTPA (0.1-mM final concentration from 100-mM stock in water), 15 µL of coumarin-azide (0.015-mM final concentration from 20-mM stock in DMSO), and 15 µL of $CuSO_4$ (0.075-mM final concentration from 100-mM stock in water) followed by incubation at rt for 0.5–18 h. The reaction solution was discard, and the membrane was rinsed with methanol and washed 1:1 DMSO/methanol mixture for 5 min. The rinsing and washing process was repeated three times total. The membrane was rinsed with methanol

and then a mixture of 7:3 ethanol/water, and the dried membrane was imaged with Amersham ImageQuant 800. Afterward, the membrane was reactivated with methanol and washed with water before Ponceau stain at rt for 5 min.

Anti-biotin western blot

The blot membrane after the transfer process was washed with methanol (>5 min) and a mixture of 7:3 ethanol/water (>5 min), and then rinsed with water. The membrane was stained by Ponceau S at rt for 5 min. After the colorimetric image was obtained, the membrane was washed with 1X TBST buffer three times and blocked with a solution 5% BSA in the TBST buffer for 20 min. Streptavidin-Cy5 conjugate solution (Jackson ImmunoResearch 016-170-084, 1:2,000) was added to the blocking solution, and the membrane was incubated at rt for 40 min in dark (covered by aluminum foil). The membrane was washed with the TBST buffer five times and imaged by Amersham ImageQuant 800.

Preparative synthesis of small molecules

Organic synthesis procedures

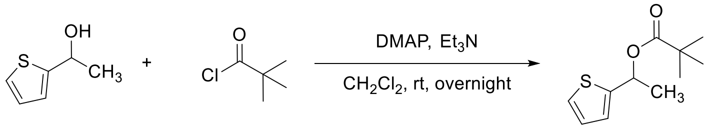

1-(thiophen-2-yl)ethyl pivalate: To a solution of 1-(thiophen-2-yl)ethan-1-ol (128 mg, 1.000 mmol), triethylamine (167 µL, 1.200 mmol) and 4-dimethylaminopyridine (24.4 mg, 0.200 mM) in dichloromethane (2 mL) in a 4 mL vial equipped with a magnetic stir bar, pivaloyl chloride (148 µL, 1.200 mmol) was added at 0 °C. The resulting mixture was stirred overnight at room temperature. After confirming product formation via TLC, the reaction mixture was quenched with saturated sodium bicarbonate (8 mL) and then partitioned between diethyl ether (6.0 mL) and H_2O (6.0 mL). The aqueous layer was washed with diethyl ether (2 × 6.0 mL). The combined organic layers were dried with anhydrous $MgSO_4$, filtered and concentrated under vacuum. The concentrated crude was purified by flash chromatography with hexanes/EtOAc as the eluent to afford a colorless oil (143 mg, 67%). ^{1}H NMR (600 MHz, $CDCl_3$) δ 7.24 (dd, *J* = 5.1, 1.3 Hz, 1H), 7.03–6.99 (m, 1H), 6.98–6.93 (m, 1H), 6.13 (q, *J* = 6.6, 1H), 1.62 (d, *J* = 6.6 Hz, 3H), 1.20 (s, 9H). ^{13}C NMR (151 MHz, $CDCl_3$) δ 177.71, 145.20, 126.63, 125.00, 124.70, 67.69, 38.84, 27.16, 22.25. IR: 1729. HRMS-ESI (m/z) $[M+Na]^+$ calcd. for $C_{11}H_{16}O_2SNa$, 235.07632; found 235.0761.

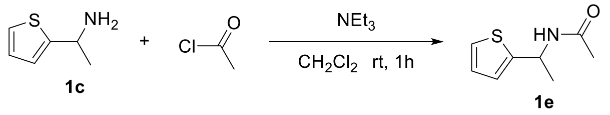

***N*-(1-(thiophen-2-yl)ethyl)acetamide (1e)**: In a 4 mL glass vial, equipped with a magnetic stir bar, mixing a solution of 1-(thiophen-2-yl)ethan-1-amine (25.0 mg, 0.197 mmol) in dichloromethane (75 µL) with triethylamine (82.2 µL, 59.7 mg, 0.590 mmol) resulted in a formation of white suspension. Into the suspension at 0 °C, a solution of acetyl chloride (236 µL, 1M, 0.236 mmol) in dichloromethane was added dropwise via a syringe and stirred for 5 mins. The resulting solution was stirred for 1 h at rt. Afterward, the reaction mixture was diluted with H_2O (2.0 mL) and then partitioned between dichloromethane (2.0 mL) and H_2O (2.0 mL). The aqueous layer was washed with dichloromethane (2 × 2.0 mL). The combined organic layers were dried with anhydrous $MgSO_4$ and filtered. The mixture was then dried under vacuum to afford a crude product which was purified by flash chromatography to afford a colorless oil (4.8 mg, 14%). ^{1}H NMR (500 MHz, $CDCl_3$) δ 7.21 (dd, *J* = 4.9, 1.4 Hz, 1H), 7.01–6.93 (m, 2H), 5.47–5.34 (m, 1H), 1.99 (s, 3H), 1.58 (d, *J* = 6.9 Hz, 4H). ^{13}C NMR (176 MHz, $CDCl_3$) δ 169.02, 147.17, 127.00, 124.47, 124.18, 44.74, 23.59, 22.32. IR: 3362, 3263, 3241, 1653. HRMS-ESI (m/z) $[M-H]^-$ calcd. for $C_9H_{13}NOS$, 168.04886; found 168.04890.

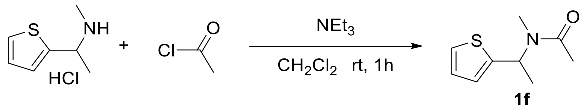

***N*-methyl-*N*-(1-(thiophen-2-yl)ethyl)acetamide (1f)**: In a 4 mL glass vial, equipped with a magnetic stir bar, mixing a solution of N-methyl-1-(2-thienyl)ethanamine hydrochloride (51.4 mg, 0.289 mmol) in dichloromethane (107 µL) with triethylamine (118 µL, 85.7 mg, 0.848 mmol) resulted in a formation of white suspension. Into the suspension at 0 °C, a solution of acetyl chloride (350µL, 1M, 0.350 mmol)

in dichloromethane was added dropwise via a syringe and stirred for 5 mins. The resulting solution was stirred for 1 h at rt. Afterward, the reaction mixture was diluted with H_2O (2.0 mL) and then partitioned between dichloromethane (2.0 mL) and H_2O (2.0 mL). The aqueous layer was washed with dichloromethane (2 × 2.0 mL). The combined organic layers were dried with anhydrous $MgSO_4$ and filtered. The mixture was then dried under vacuum to afford a colorless oil of the product as a mixture of rotamers (37.2 mg, 70%) as a mixture of rotamers, confirmed by ^1H NMR. ^1H NMR (500 MHz, $CDCl_3$, mixture of rotamers) δ 7.24–6.82 (m, 3H), 6.33–5.13 (m, 1H), 2.84–2.43 (m, 3H), 2.34–2.03 (m, 3H), 1.74–1.46 (m, 3H). ^{13}C NMR (176 MHz, $CDCl_3$, mixture of rotamers) δ 170.39, 170.00, 145.29, 145.03, 126.97, 126.60, 125.13, 124.90, 124.81, 124.58, 52.81, 46.93, 29.99, 27.33, 22.32, 21.68, 19.05, 17.48. IR: 1633. HRMS-ESI (m/z) $[M+H]^+$ calcd. for $C_9H_{13}NOS$, 184.0791; found 184.07906.

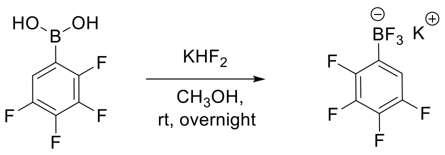

2p

Potassium 2,3,4,5-tetrafluoroborate (2p): To a solution of 2,3,4,5-tetrafluorphenylboroinc acid (23.2 mg, 0.120 mmol) in H_2O (600 µL) in a 1.7 mL Eppendorf tube, a solution of potassium hydrogenfluroride (222 µL, 4 M, 0.888 mmol) in H_2O was added and stirred overnight at rt. The resulting mixture was dried under gentle flow of nitrogen gas. The solid residue was suspended with acetone, filtered, and dried under vacuum. The resulting solid was washed with Et_2O (3 × 1 mL) and dried under high vacuum to afford a white solid (22.1 mg, 72%). Spectral data in CD_3CN is consistent with previous reports.[4,5] ^{1}H NMR (700 MHz, DMSO-d_6) δ 7.00 – 6.93 (m, 1H). ^{13}C NMR (176 MHz, DMSO-d_6) δ 148.86 (d, *J* = 6.5 Hz), 147.51 (d, *J* = 6.6 Hz), 146.18 (m), 144.82 (m), 139.66– 139.55 (m), 138.20–138.05 (m), 136.81–136.64 (m), 113.35–113.18 (m).

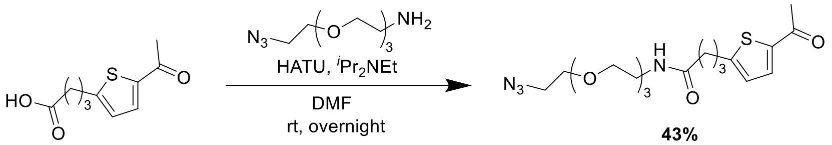

43%

4-(5-acetylthiophen-2-yl)-N-(2-(2-(2-(2-azidoethoxy)ethoxy)ethoxy)ethyl)butanamide: To a mixture of 4-(5-acetylthiophen-2-yl)butanoic acid (60.0 mg, 0.283 mmol) and HATU (73.2 mg, 0.311 mmol) in a 4 mL vial equipped with a magnetic stir bar, dimethylformamide (0.275mL) was added. The reaction mixture was cooled in an ice bath for 2 min, after which Hunig base (43.8 mg, 0.339 mmol) was added and stirred for 5 min. The mixture was cooled in an ice bath again and then 1-amino-11-azido-3,6,9-trioxaundecane (92.5 mg, 0.424 mmol) was added. After the reaction mixture was stirred overnight at rt, all volatiles were removed by a gentle flow of N_2 gas. Afterward, CH_2Cl_2 (7 mL) was added, and the resulting mixture was washed with brine (7 mL). The CH_2Cl_2 layer was further washed with $KHSO_4$ (1 M, 3 × 5 mL), saturated $NaHCO_3$ (3 × 5 mL), and brine (7 mL), dried with Na_2SO_4, and filtered. The resulting solution was dried under vacuum to afford the crude product which was purified by Yamazen Smart Flash W-Prep dual channel chromatography (ethyl acetate as an eluent) to afford a brown-orange liquid (50.0 mg, 43%). ^{1}H NMR (500 MHz, $CDCl_3$): δ 7.53 (d, *J* = 3.8 Hz, 1H), 6.84 (d, *J* = 3.8 Hz, 1H), 6.04 (s, 1H), 3.68-3.61 (m, 10H) 3.55 (t, J = 5.6 Hz, 2H), 3.45 (q, *J* = 5.3 Hz, 2H), 3.39 (t, *J* = 5.1 Hz, 2H), 2.89(t, *J* = 7.5 Hz, 2H), 2.51 (s, 3H), 2.23(t, *J* = 7.4 Hz, 2H), 2.04 (m, 2H).^{13}C NMR (700 MHz, CD_3CN): δ 191.0, 172.6, 155.3, 142.9, 134.0, 126.9, 70.7, 70.5, 70.0, 69.9, 51.0, 39.4, 35.3, 30.0, 27.6, 26.3. IR: 3317, 2102, 1666. HRMS-ESI (m/z) $[M+H]^+$calcd for $C_{18}H_{29}N_4O_5S$, 413.1849; found 413.18532.

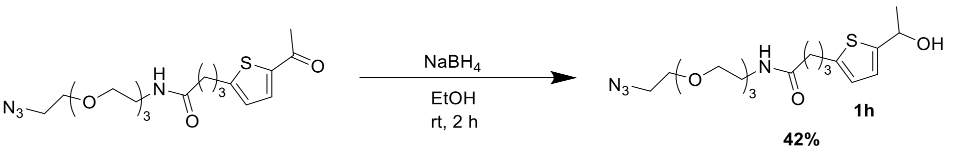

1h

42%

N-(2-(2-(2-(2-azidoethoxy)ethoxy)ethoxy)ethyl)-4-(5-(1-hydroxyethyl)thiophen-2 yl)butanamide (1h): To 4-(5-acetylthiophen-2-yl)-N-(2-(2-(2-(2-azidoethoxy)ethoxy)ethoxy)ethyl)butanamide (23.0 mg, 0.056 mmol) dissolved in ethanol (0.198 mL), $NaBH_4$ (6.33 mg, 0.167 mmol) was added in one portion and stirred for 2 h at rt. Ethanol was removed by a gentle flow of N_2 gas. Afterward, H_2O (7 mL), ethyl acetate (7 mL), and brine (2 mL) were added, and the product was extracted using ethyl acetate (2 × 6 mL). The combined organic layer was back washed with brine (11 mL), dried with

Na_2SO_4, and filtered. The mixture was dried under vacuum to obtain a viscous brown-orange liquid. (10 mg, 42%) ^{1}H NMR (500 MHz, $CDCl_3$): δ 6.78 (d, J = 3.4 Hz, 1H), 6.64 (d, J = 3.4 Hz, 1H), 6.02 (s, 1H), 5.04 (q, J = 6.5 Hz, 1H), 3.68–3.61 (m, 10H), 3.55 (t, *J* = 5.6 Hz, 2H), 3.45 (q, *J* = 5.3 Hz, 2H), 3.38 (t, *J* = 5.1 Hz, 2H), 2.83 (t, J = 7.2 Hz, 2H), 2.22 (t, *J* = 7.4 Hz, 2H), 2.00-1.92 (m, 3H), 1.57 (d, *J* = 6.4 Hz, 3H). ^{13}C NMR (700 MHz, CD_3CN): δ 173.1, 149.6, 144.3, 124.8, 123.5, 71.1, 70.9, 70.5, 70.3, 66.3, 51.5, 39.8, 35.8, 29.9, 28.6, 25.7. IR: 3326, 2102, 1647. HRMS-ESI (m/z) $[M+Na]^+$calcd for $C_{18}H_{30}N_4O_5SNa$, 437.1821; found 437.18291.

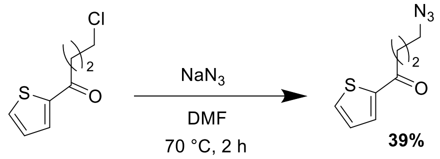

4-azido-1-(thiophen-2-yl)butan-1-one: 4-chloro-1-(thiophen-2-yl)butan-1-one (30 mg, 0.159 mmol) and NaN_3 (21 mg , 0.318 mmol) were suspended in DMF (0.638 mL) and the reaction mixture was stirred at 70 ̊C for 2 h. After completion of the reaction, the reaction mixture was diluted with Et_2O (3 × 10 ml) and washed with water (3 × 10 ml). The organic layer was separated and dried over anhydrous Na_2SO_4. The solvent was removed under a gentle flow of N_2 gas to obtain a colorless oil (12.0 mg, 39%). The small molecule product did not give any meaningful signals in the high-resolution mass spectrometry analysis. ^{1}H NMR (700 MHz, CD_3CN): δ 7.82 (dd, *J* = 3.8, 1.1 Hz, 1H), 7.77 (dd, *J* = 5.0, 1.2 Hz, 1H), 7.19 (dd, *J* = 5.0, 3.8 Hz, 1H), 3.39 (t, *J* = 6.9 Hz, 2H), 3.03 (t, *J* = 7.2 Hz, 2H), 1.97-1.95 (m, 2H); ^{13}C NMR (700 MHz, CD_3CN) δ 193.3, 145.0, 135.0, 133.5, 129.4, 51.5, 36.5, 24.3. IR: 2095, 1662.

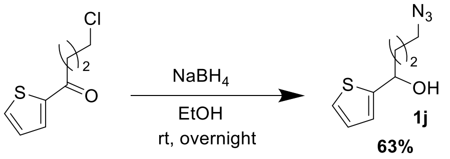

4-azido-1-(thiophen-2-yl)butan-1-ol 1j : $NaBH_4$(4 mg, 0.132 mmol) was added into the solution with 4-azido-1-(thiophen-2-yl)butan-1-one (15 mg, 0.0768 mmol) in ethanol (0.384 mL). The resulting mixture was stirred overnight at rt. Then, the solution was concentrated under a flow of N_2 gas and H_2O (1 mL) was added. Afterwards, the mixture was extracted with dichloromethane (3 × 1 ml). The combined organic layers were washed with brine and then dried with anhydrous Na_2SO_4. The desired alcohol can be obtained after removing the solvent under a gentle flow of N_2 gas (9.5 mg, 63%). Because of the volatile and potentially explosive nature of the product as a relatively small azide compound, the drying process under high vacuum was omitted, and therefore, a small amount of CH_2Cl_2 was observed in ^{1}H NMR spectrum of the product. The small molecule product did not give any meaningful signals in the high-resolution mass spectrometry analysis. ^{1}H NMR (700 MHz, CD_3CN): δ 7.29 (dd, *J* = 4.8, 1.4 Hz, 1H),6.98-6.93 (m, 2H), 4.91-4.86(m, 1H), 3.57 (d, *J* = 4.8 Hz, 1H), 3.32 (t, *J* = 6.9 Hz, 2H), 1.85-1.77 (m, 2H), 1.73-1.66 (m, 1H), 1.62-1.55 (m, 1H);^{13}C NMR (700 MHz, CD_3CN) δ 150.7, 127.5, 125.0, 124.2, 69.7, 51.9, 37.3, 25.9. IR: 3362, 2095.

High-resolution mass spectrometry (HRMS) spectra of synthesized compounds

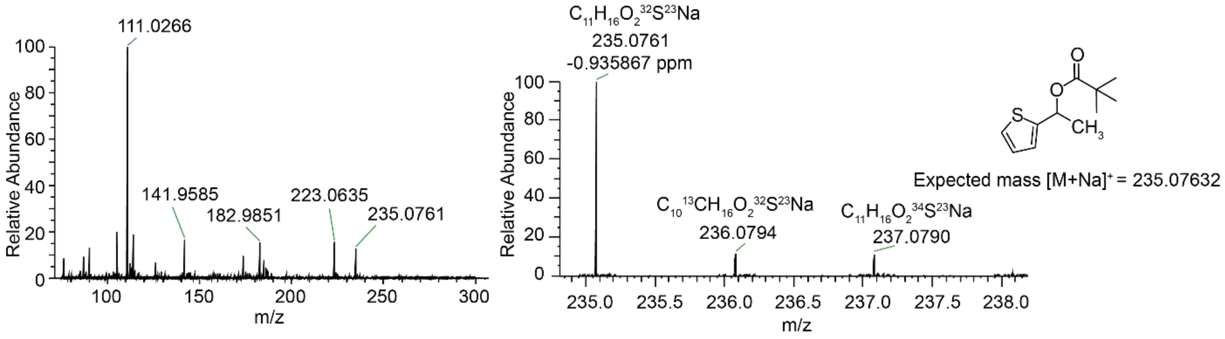

Figure S15. HRMS-ESI spectra of 1-(thiophen-2-yl)ethyl pivalate

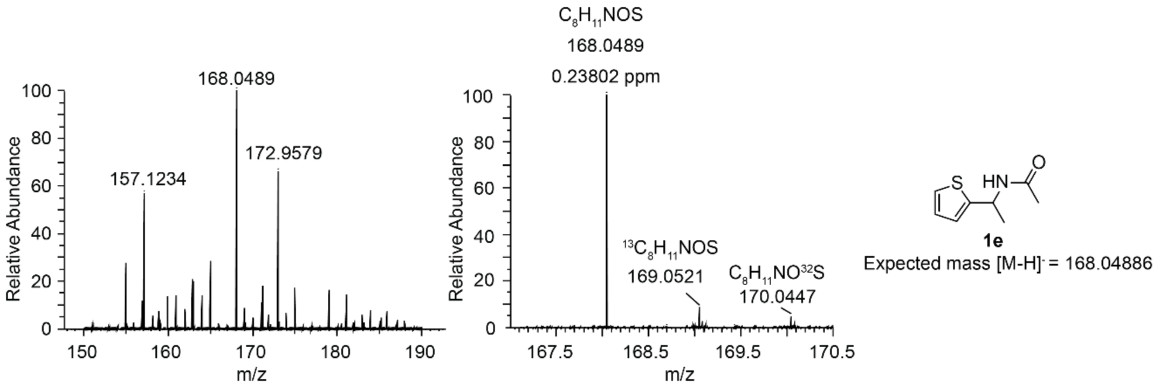

Figure S16. HRAM-ESI spectra of *N*-(1-(thiophen-2-yl)ethyl)acetamide (**1e**).

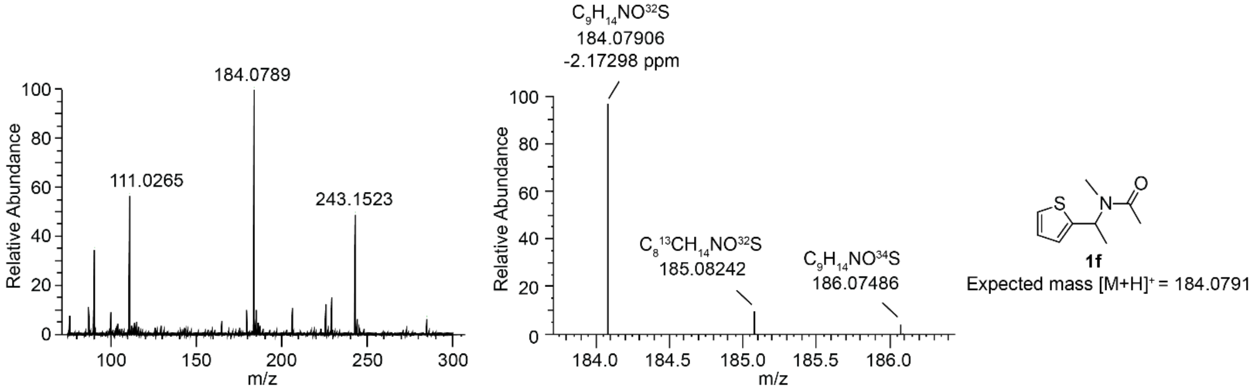

Figure S17. HRAM-ESI spectra of *N*-methyl-*N*-(1-(thiophen-2-yl)ethyl)acetamide (**1f**).

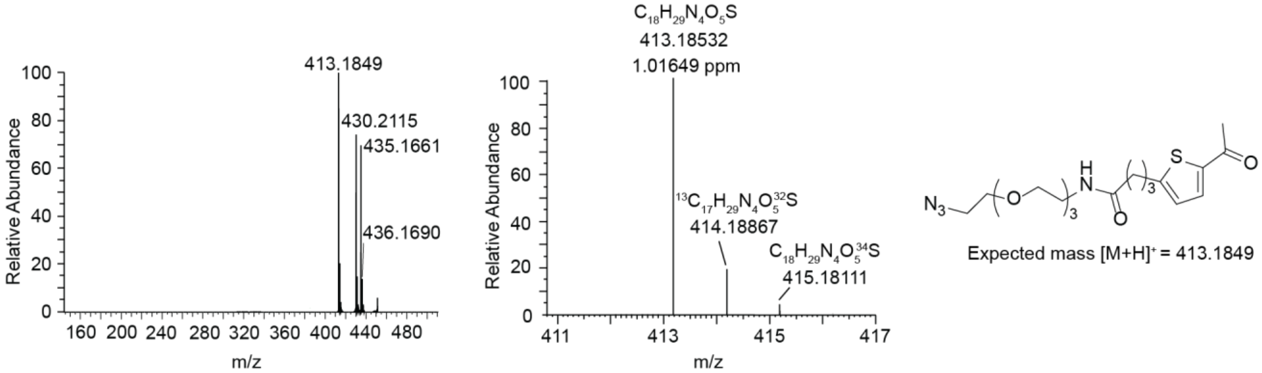

Figure S18. HRMS-ESI spectra of 4-(5-acetylthiophen-2-yl)-N-(2-(2-(2-(2-azidoethoxy)ethoxy)ethoxy)ethyl)butanamide

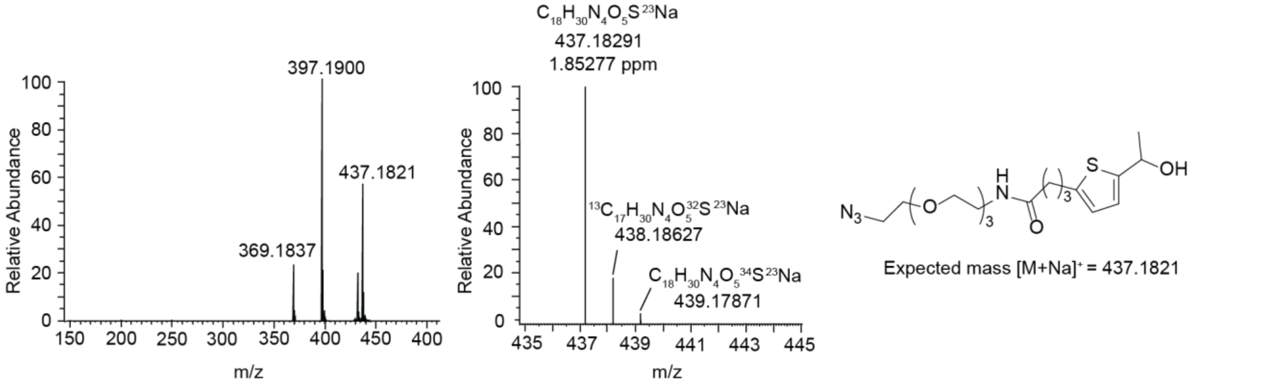

Figure S19. HRMS-ESI spectra of N-(2-(2-(2-(2-azidoethoxy)ethoxy)ethoxy)ethyl)-4-(5-(1-hydroxyethyl)thiophen-2 yl)butanamide.

Infrared (IR) spectra of the synthesized compounds

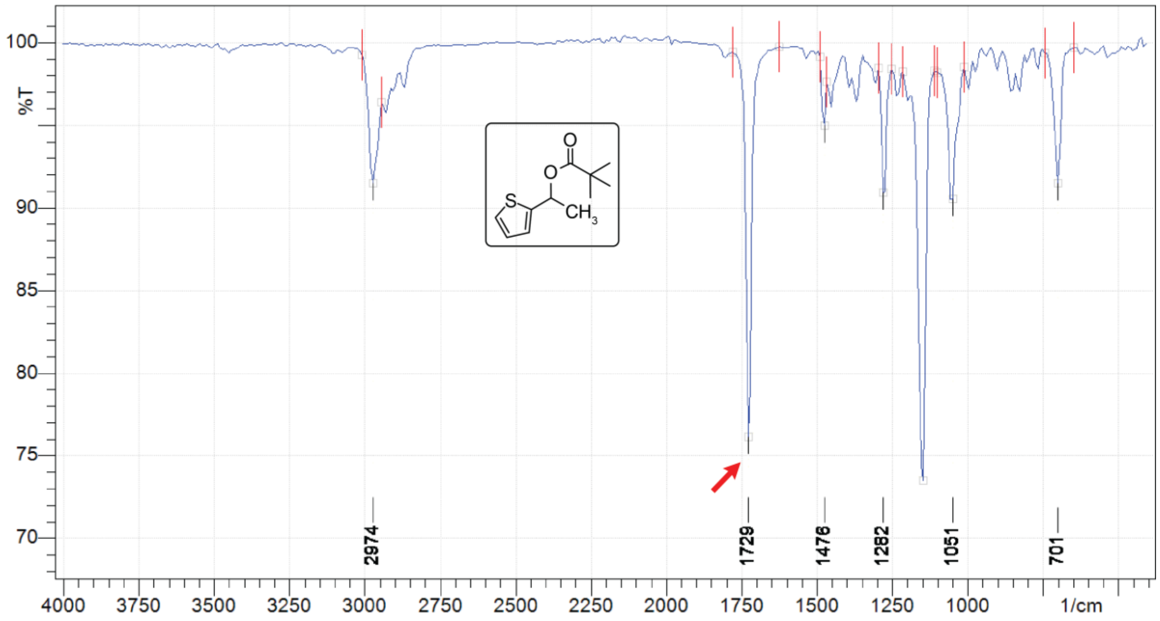

Figure S20. FT-IR spectrum of 1-(thiophen-2-yl)ethyl pivalate Relevant peaks are labeled with red arrows

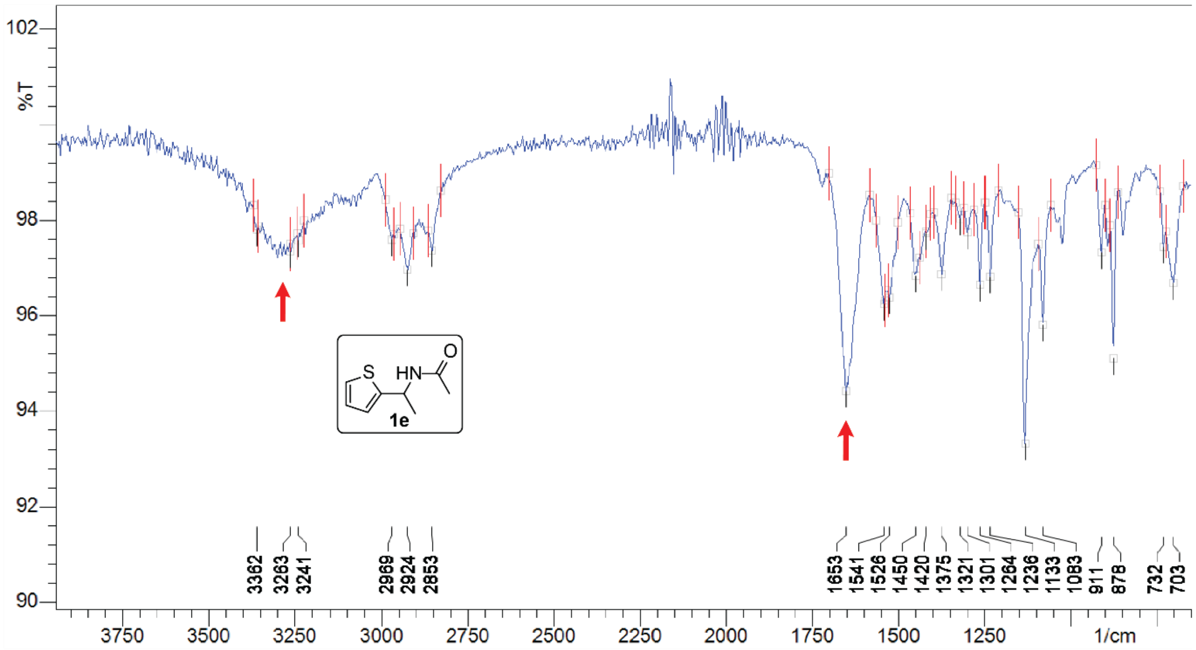

Figure S21. FT-IR spectrum of *N*-(1-(thiophen-2-yl)ethyl)acetamide (**1e**). Relevant peaks are labeled with red arrows.

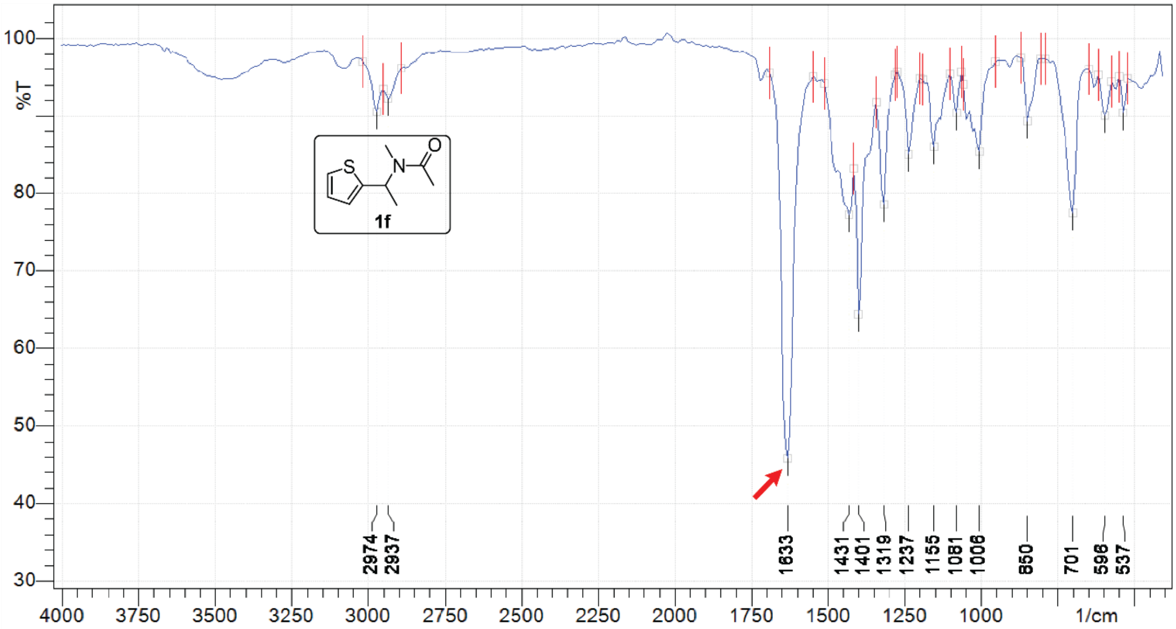

Figure S22. FT-IR spectrum of *N*-methyl-*N*-(1-(thiophen-2-yl)ethyl)acetamide (**1f**). Relevant peaks are labeled with red arrows.

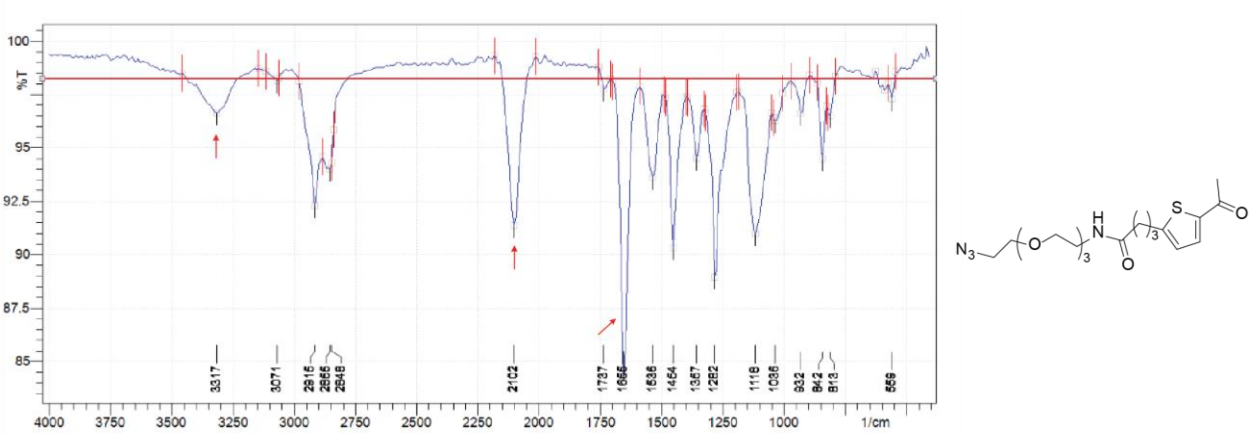

Figure S23. FT-IR spectrum of 4-(5-acetylthiophen-2-yl)-N-(2-(2-(2-(2-azidoethoxy)ethoxy)ethoxy)ethyl)butanamide. Relevant peaks are labeled with red arrows.

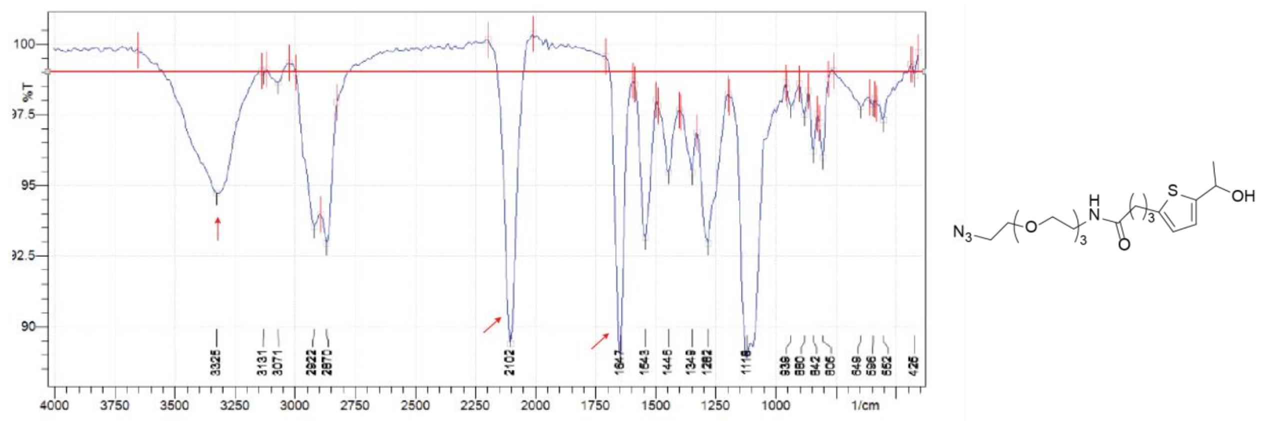

Figure S24. FT-IR spectrum of N-(2-(2-(2-(2-azidoethoxy)ethoxy)ethoxy)ethyl)-4-(5-(1-hydroxyethyl)thiophen-2 yl)butanamide. Relevant peaks are labeled with red arrows.

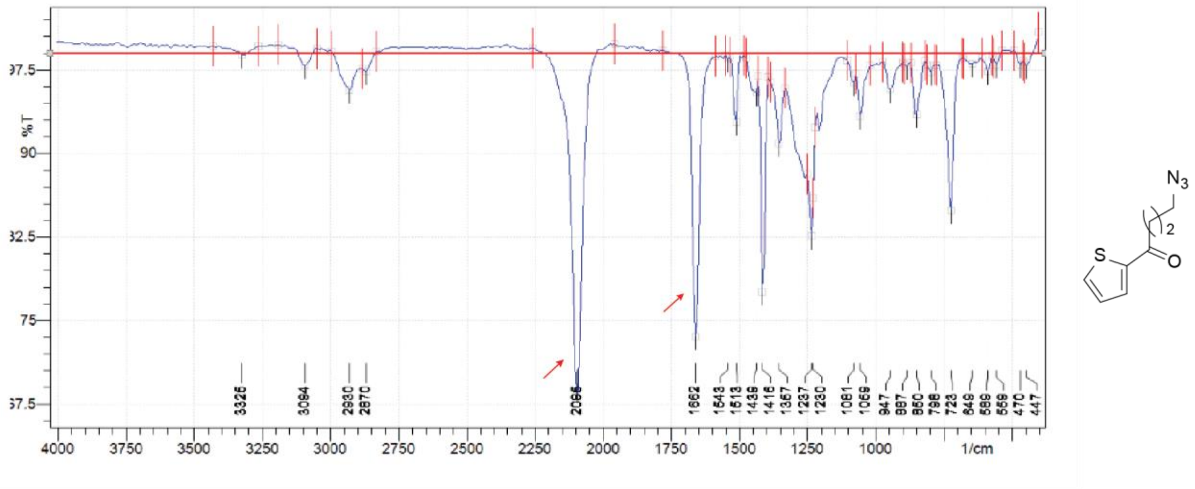

Figure S25. FT-IR spectrum of 4-azido-1-(thiophen-2-yl)butan-1-one. Relevant peaks are labeled with red arrows.

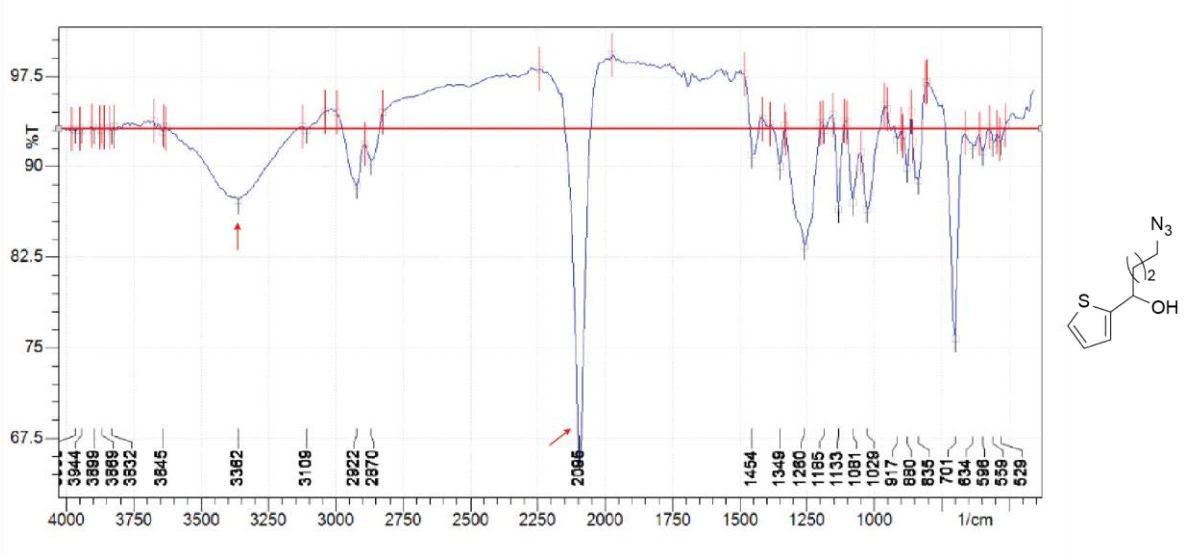

Figure S26. FT-IR spectrum of 4-azido-1-(thiophen-2-yl)butan-1-ol. Relevant peaks are labeled with red arrows.

NMR spectra of synthesized compounds

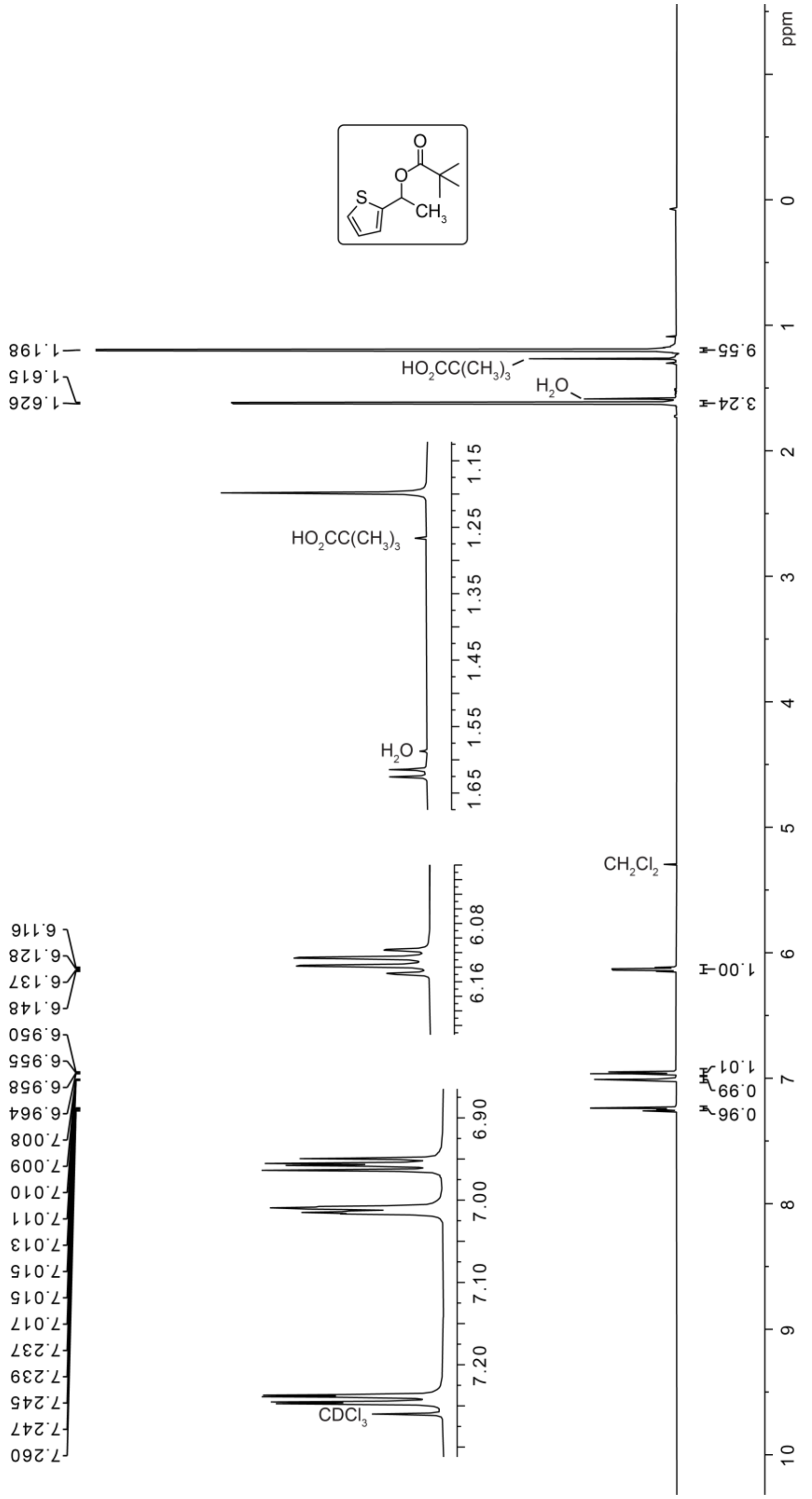

Figure S27. ^{1}H NMR spectrum of 1-(thiophen-2-yl)ethyl pivalate in $CDCl_3$.

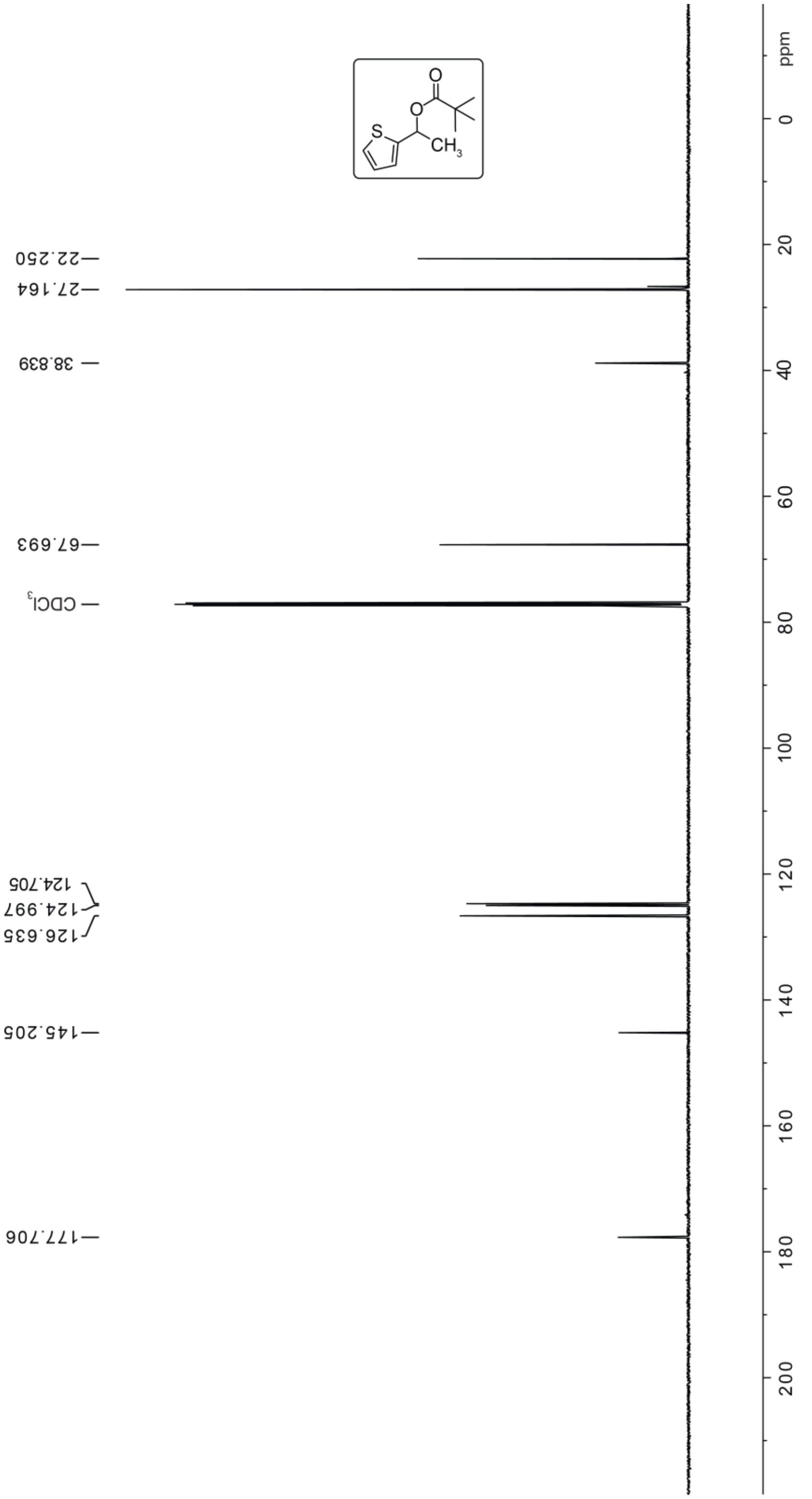

Figure S28. ^{13}C NMR spectrum of 1-(thiophen-2-yl)ethyl pivalate $CDCl_3$.

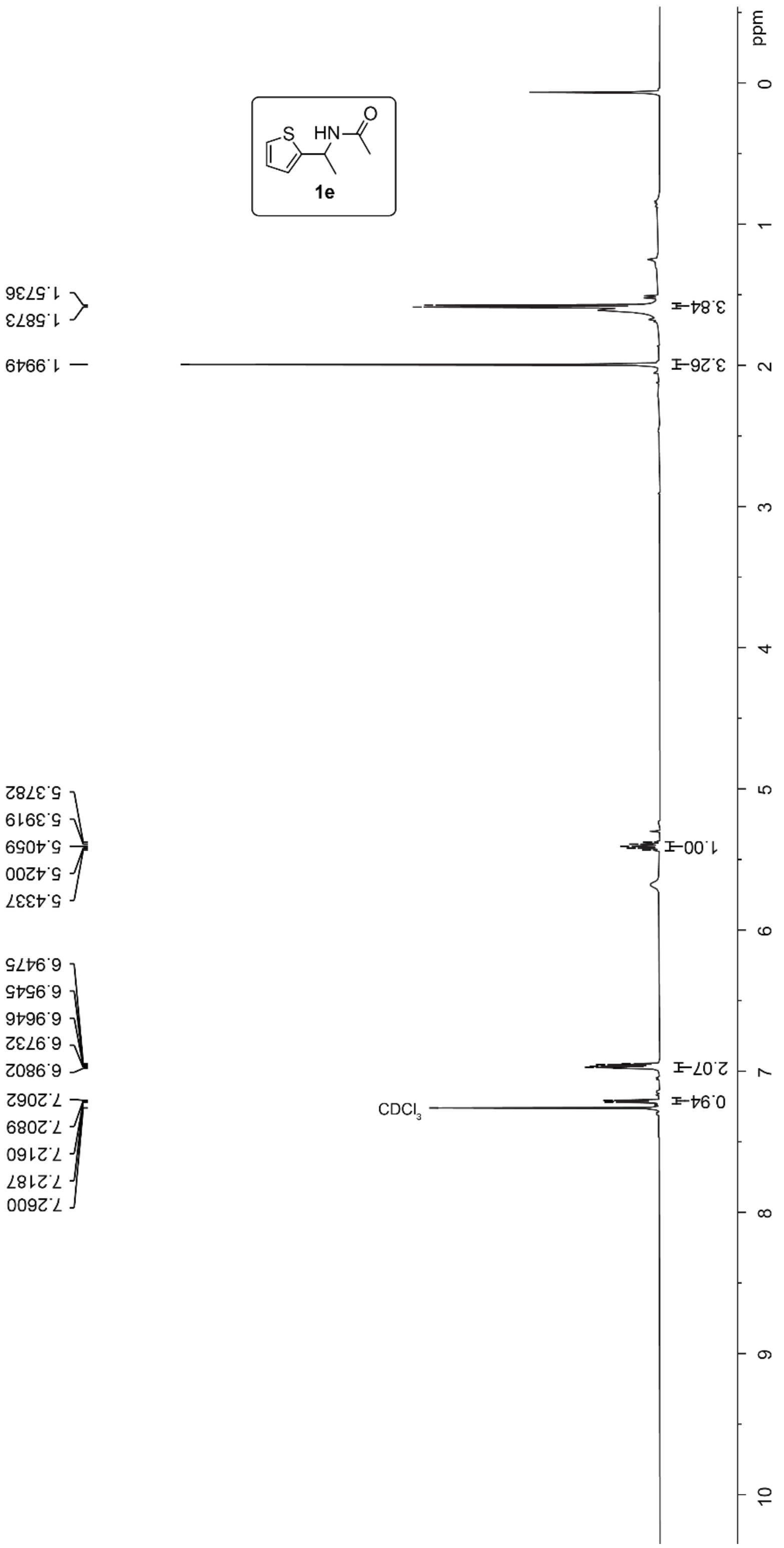

Figure S29. ^{1}H NMR spectrum of *N*-(1-(thiophen-2-yl)ethyl)acetamide (**1e**) in $CDCl_3$.

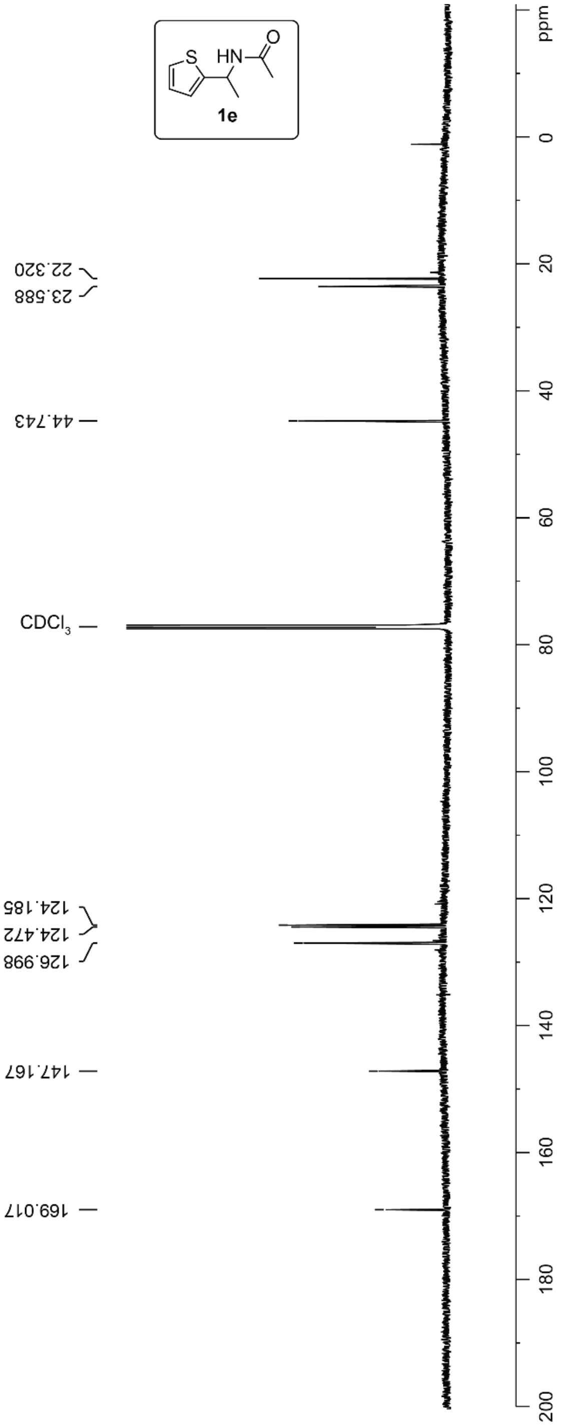

Figure S30. ^{13}C NMR spectrum of *N*-(1-(thiophen-2-yl)ethyl)acetamide (**1e**) $CDCl_3$.

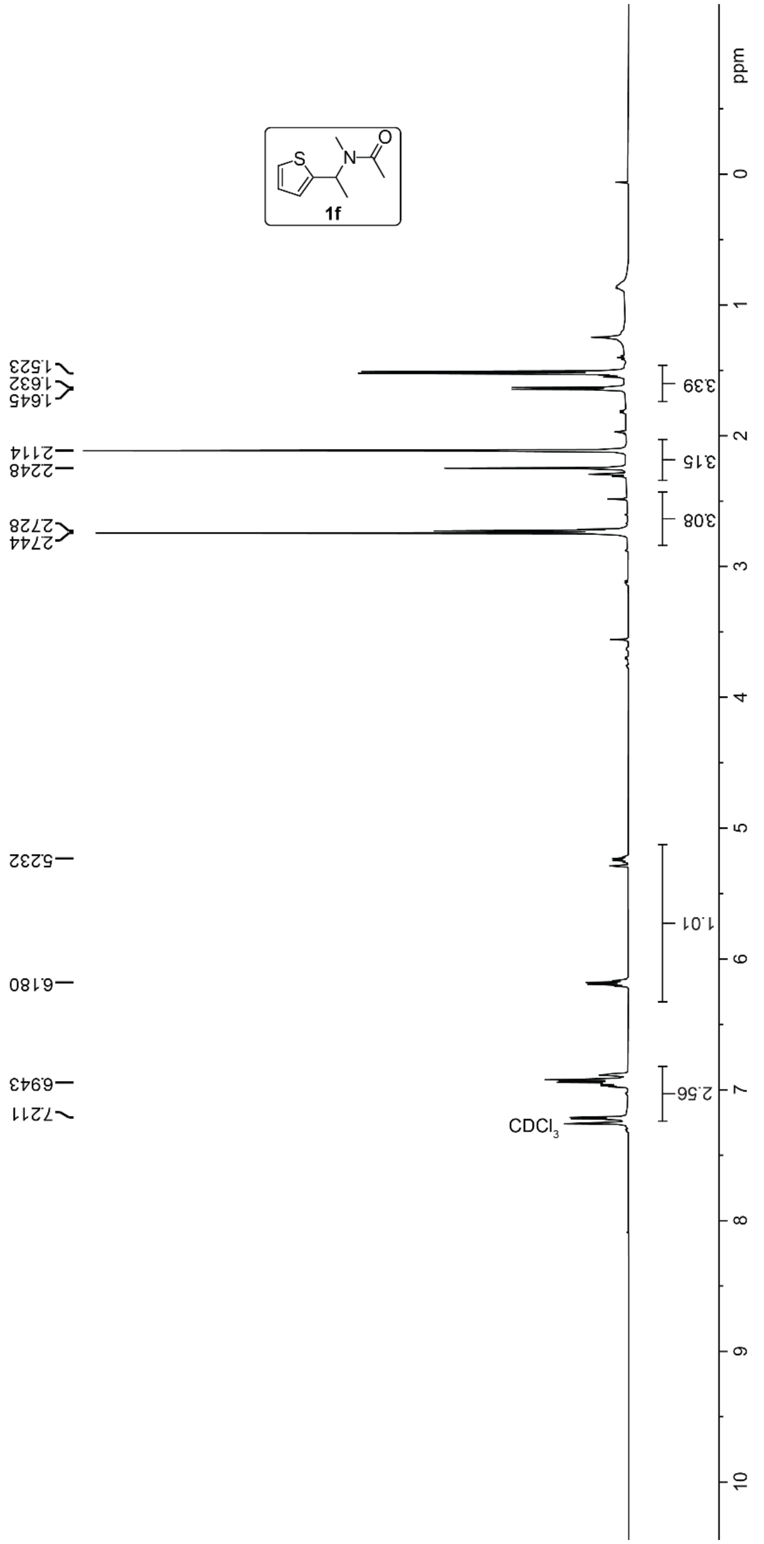

Figure S31. ^{1}H NMR spectrum of *N*-methyl-*N*-(1-(thiophen-2-yl)ethyl)acetamide (**1f**) in $CDCl_3$.

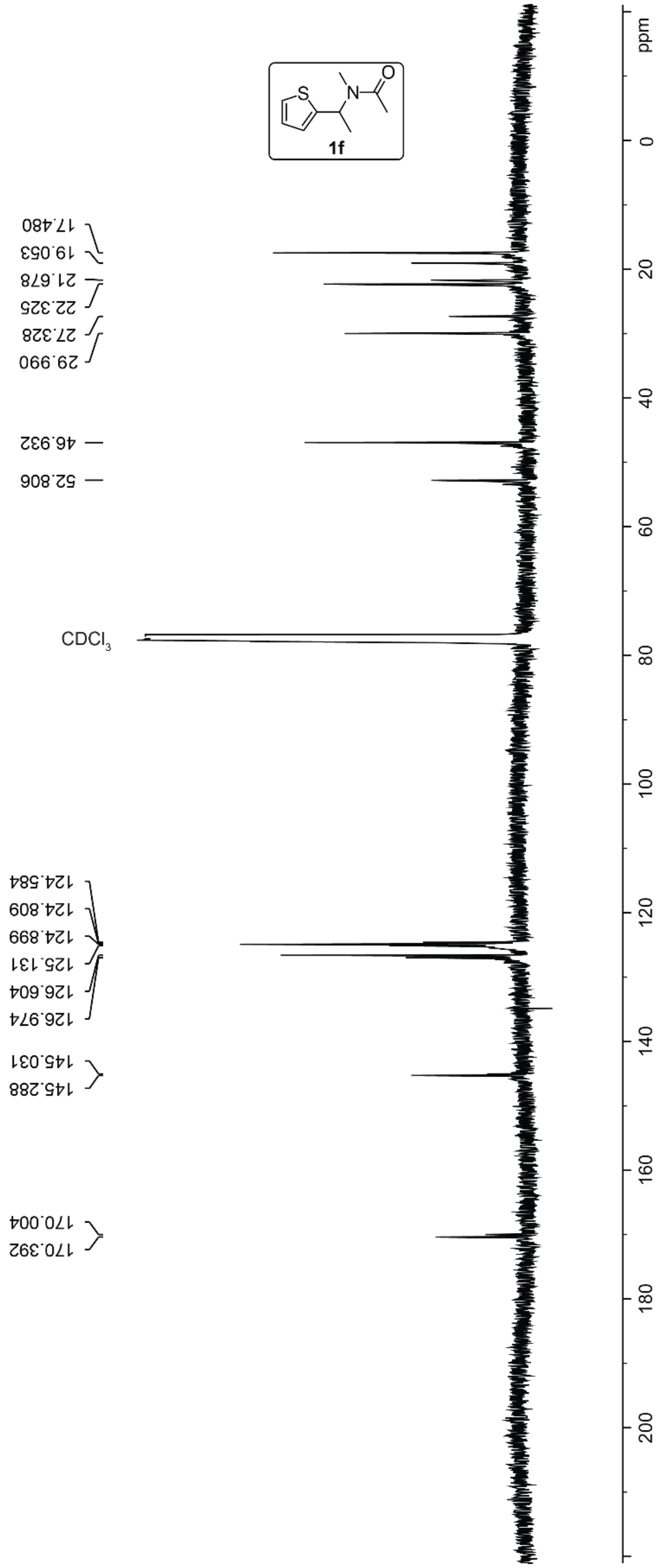

Figure S 32. ^{13}C NMR spectrum of *N*-methyl-*N*-(1-(thiophen-2-yl)ethyl)acetamide (**1f**) in $CDCl_3$.

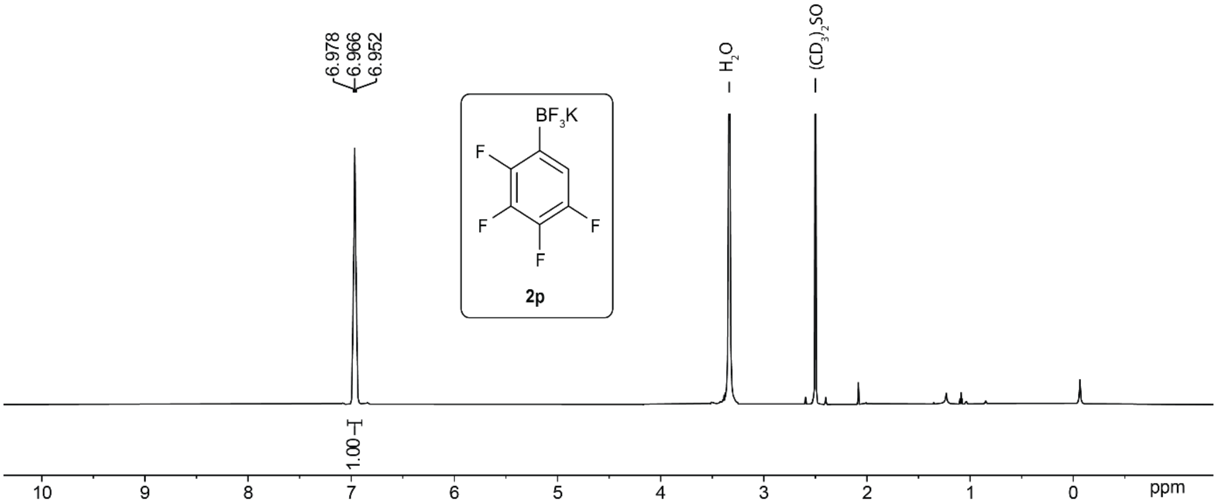

Figure S 33. ^{1}H NMR spectrum of potassium 2,3,4,5-tetrafluorophenyltrifluoroborate (**2p**) in DMSO-d_6.

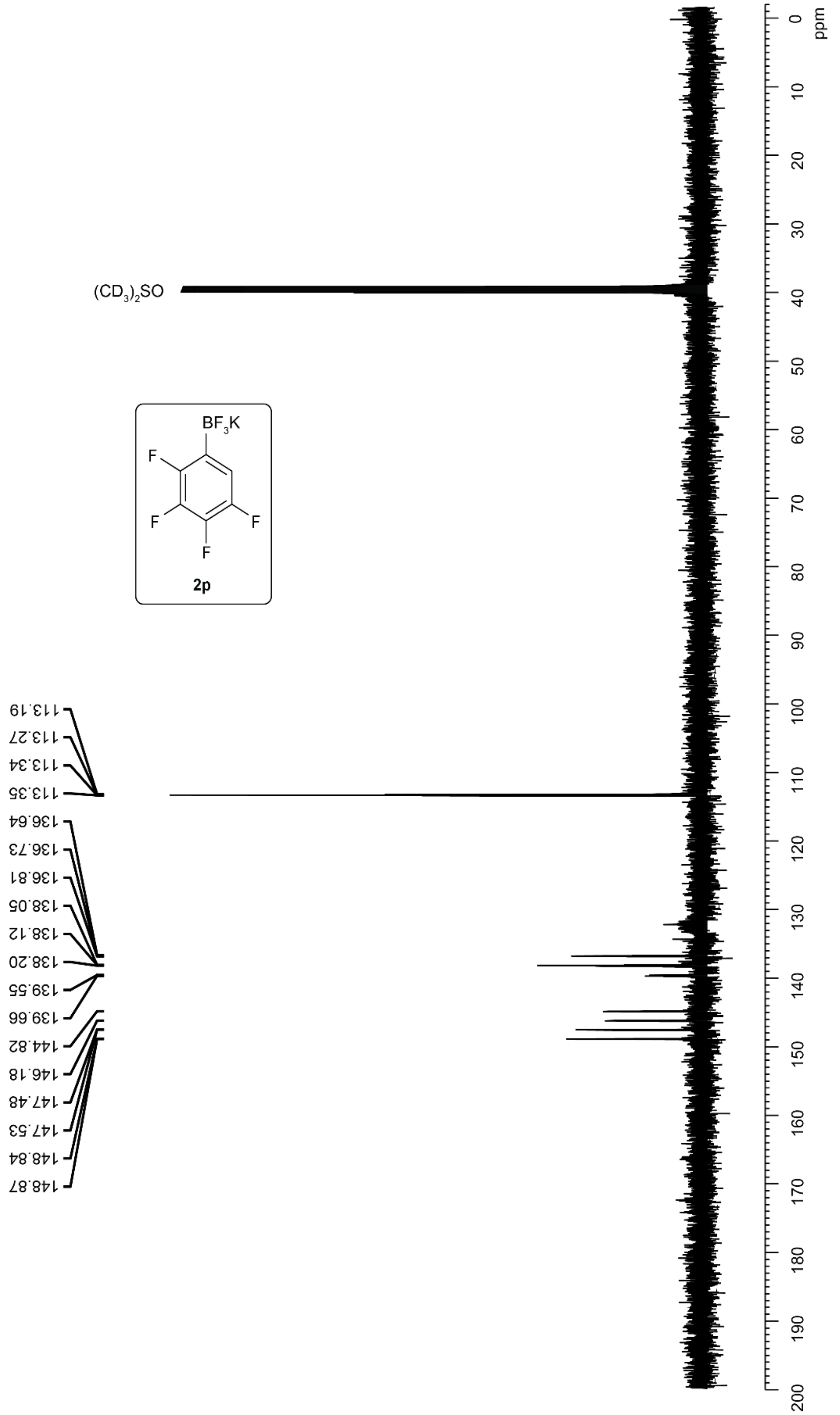

Figure S34. ^{13}C NMR spectrum of potassium 2,3,4,5-tetrafluorophenyltrifluoroborate (**2p**) in DMSO-d_6.

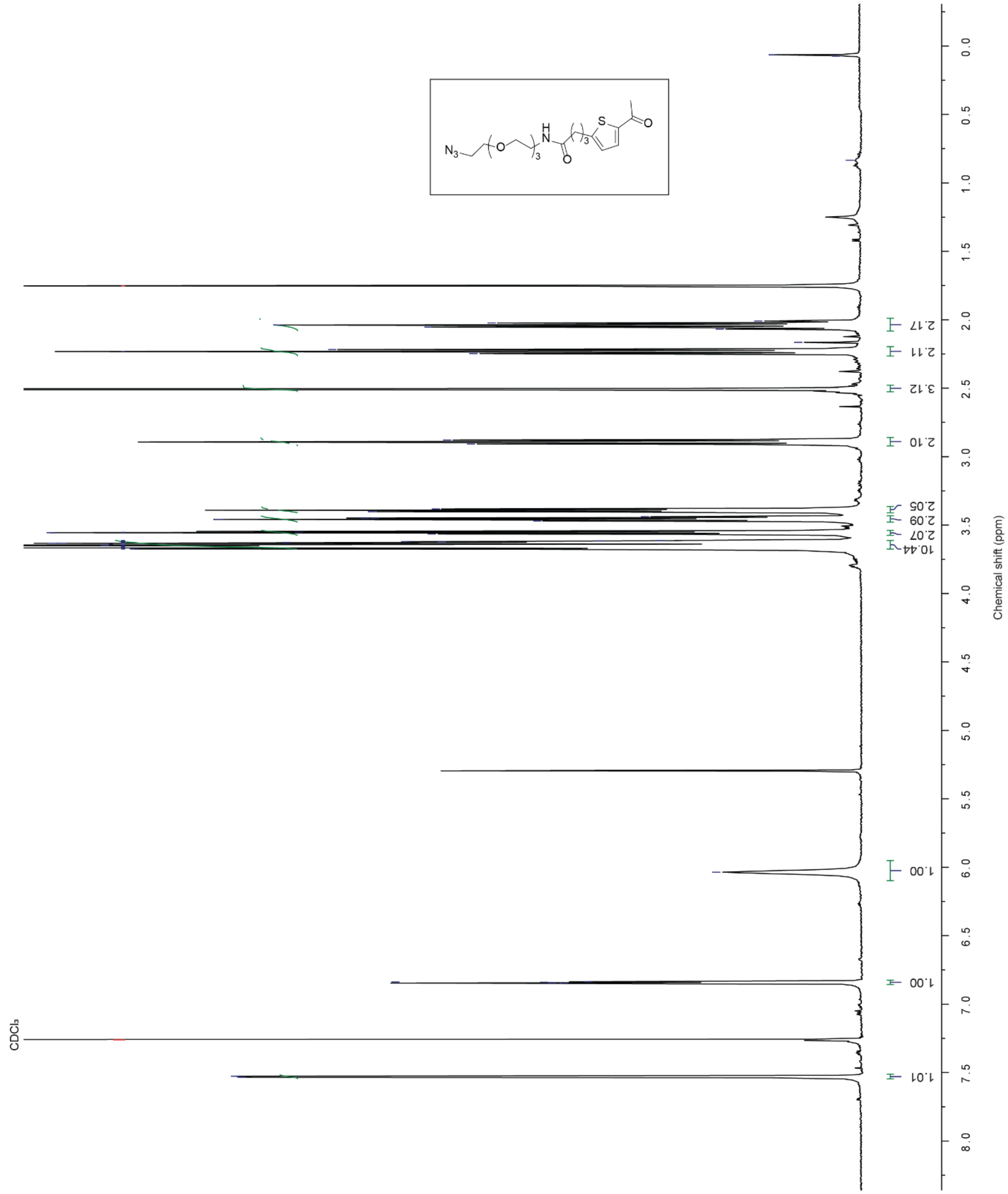

Figure S35. ^{1}H NMR spectrum of 4-(5-acetylthiophen-2-yl)-N-(2-(2-(2-(2-azidoethoxy)ethoxy)ethoxy)ethyl)butanamide in $CDCl_3$.

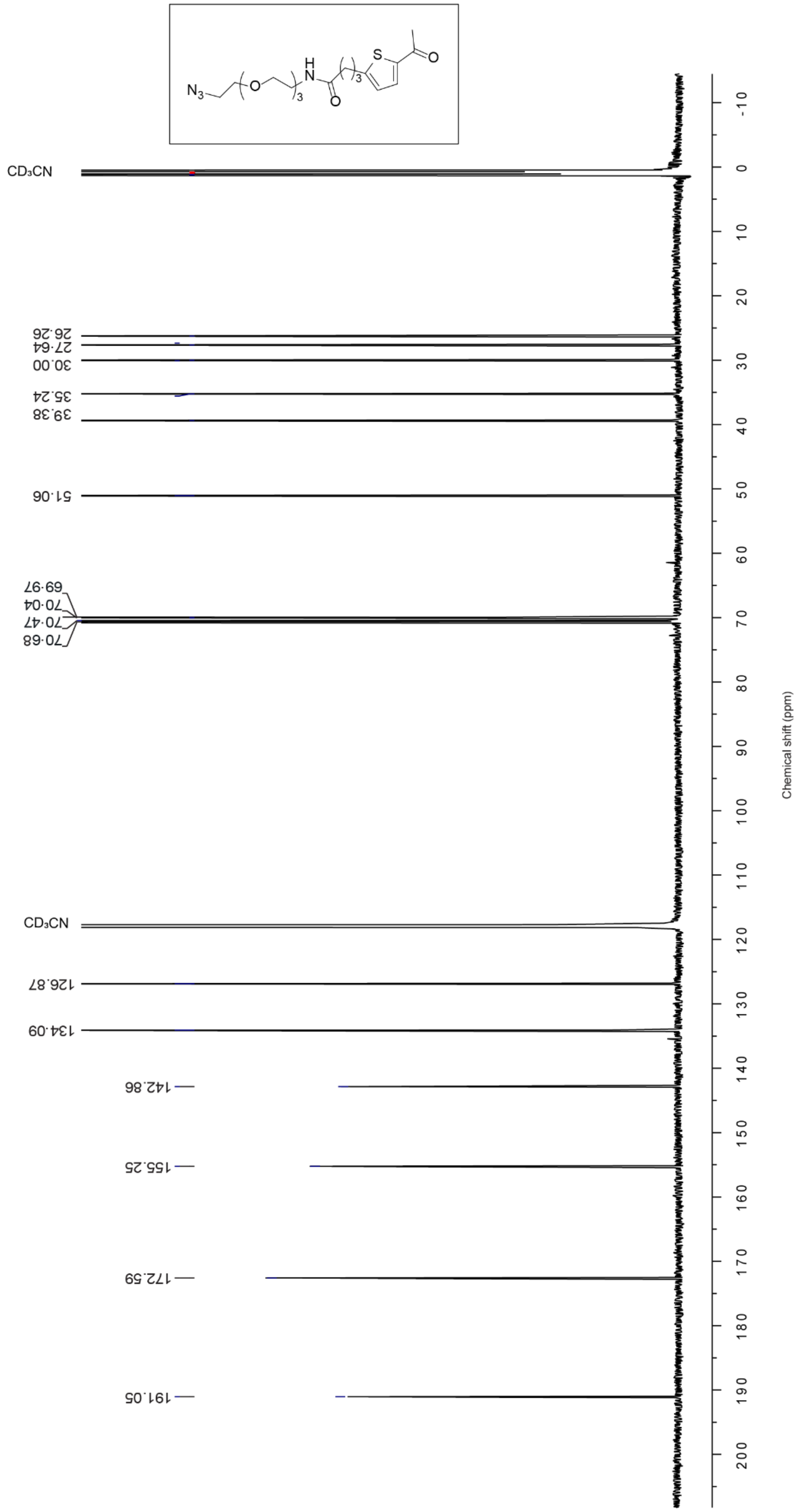

Figure S36. ^{13}C NMR spectrum of.4-(5-acetylthiophen-2-yl)-N-(2-(2-(2-(2 azidoethoxy)ethoxy)ethoxy)ethyl)butanamide in CD_3CN.

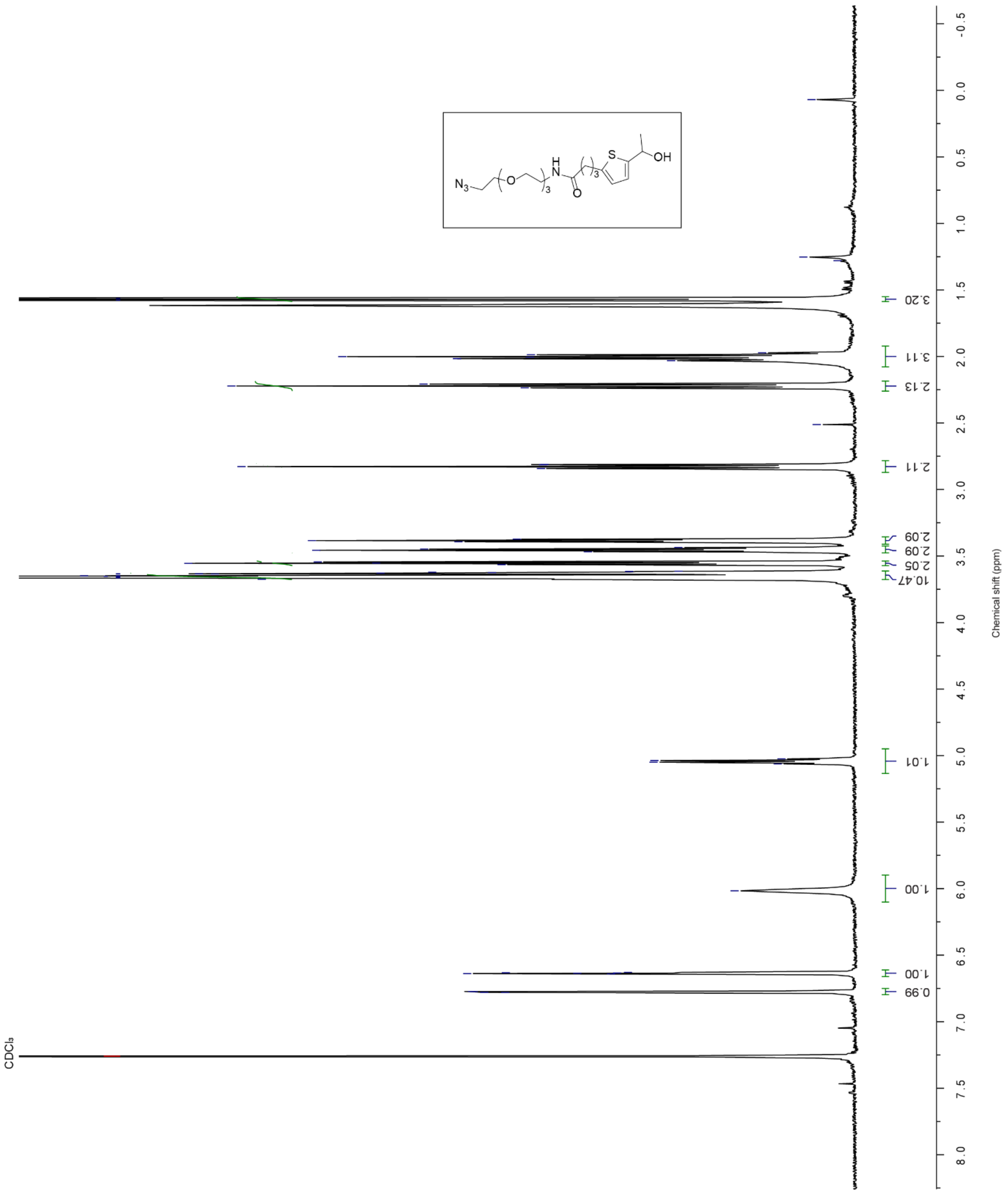

Figure S37. ^{1}H NMR spectrum of N-(2-(2-(2-(2-azidoethoxy)ethoxy)ethoxy)ethyl)-4-(5-(1-hydroxyethyl)thiophen-2 yl)butanamide in $CDCl_3$.

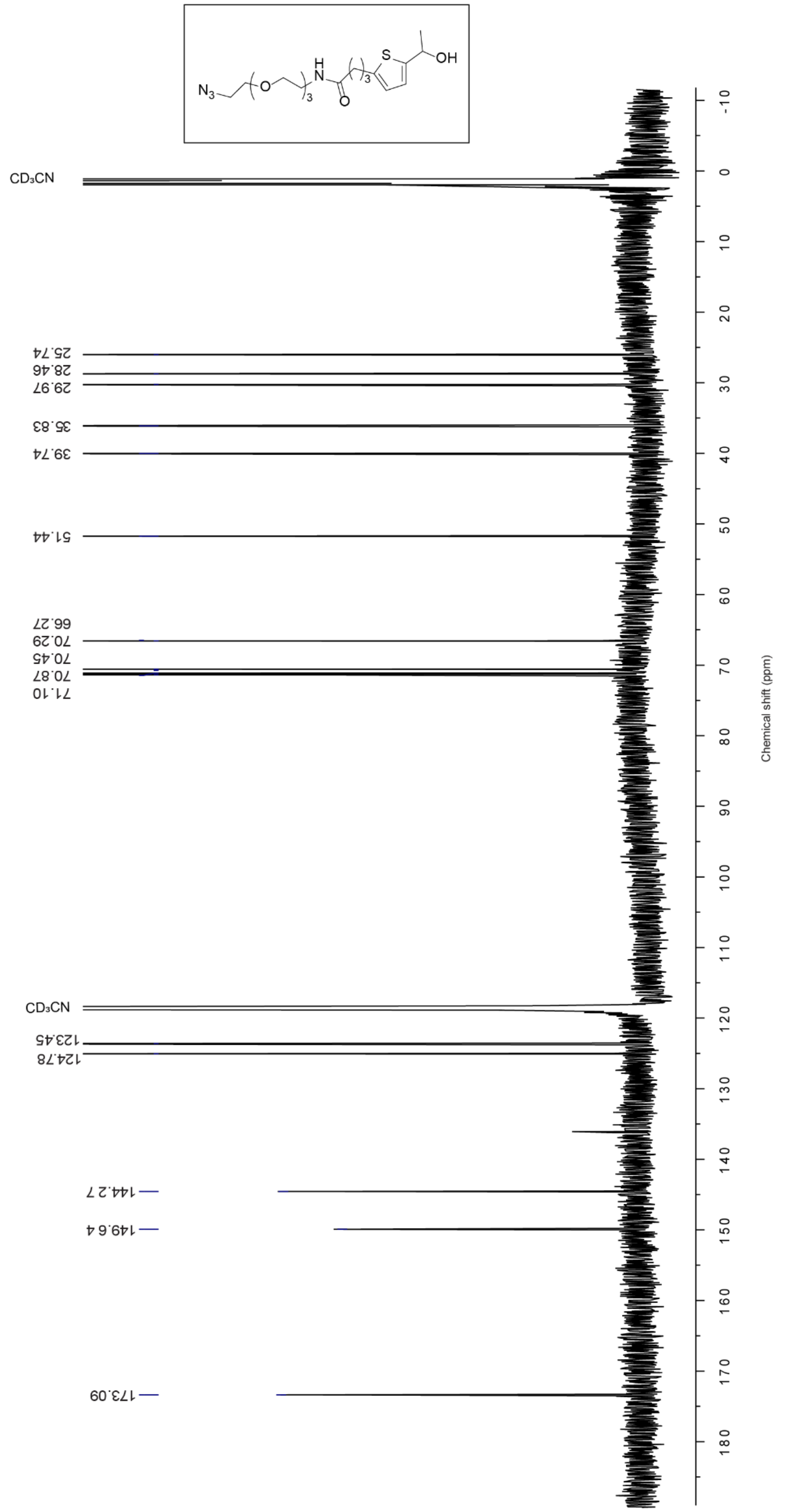

Figure S38. ^{13}C NMR spectrum of. N-(2-(2-(2-(2-azidoethoxy)ethoxy)ethoxy)ethyl)-4-(5-(1-hydroxyethyl)thiophen-2 yl)butanamide in CD_3CN.

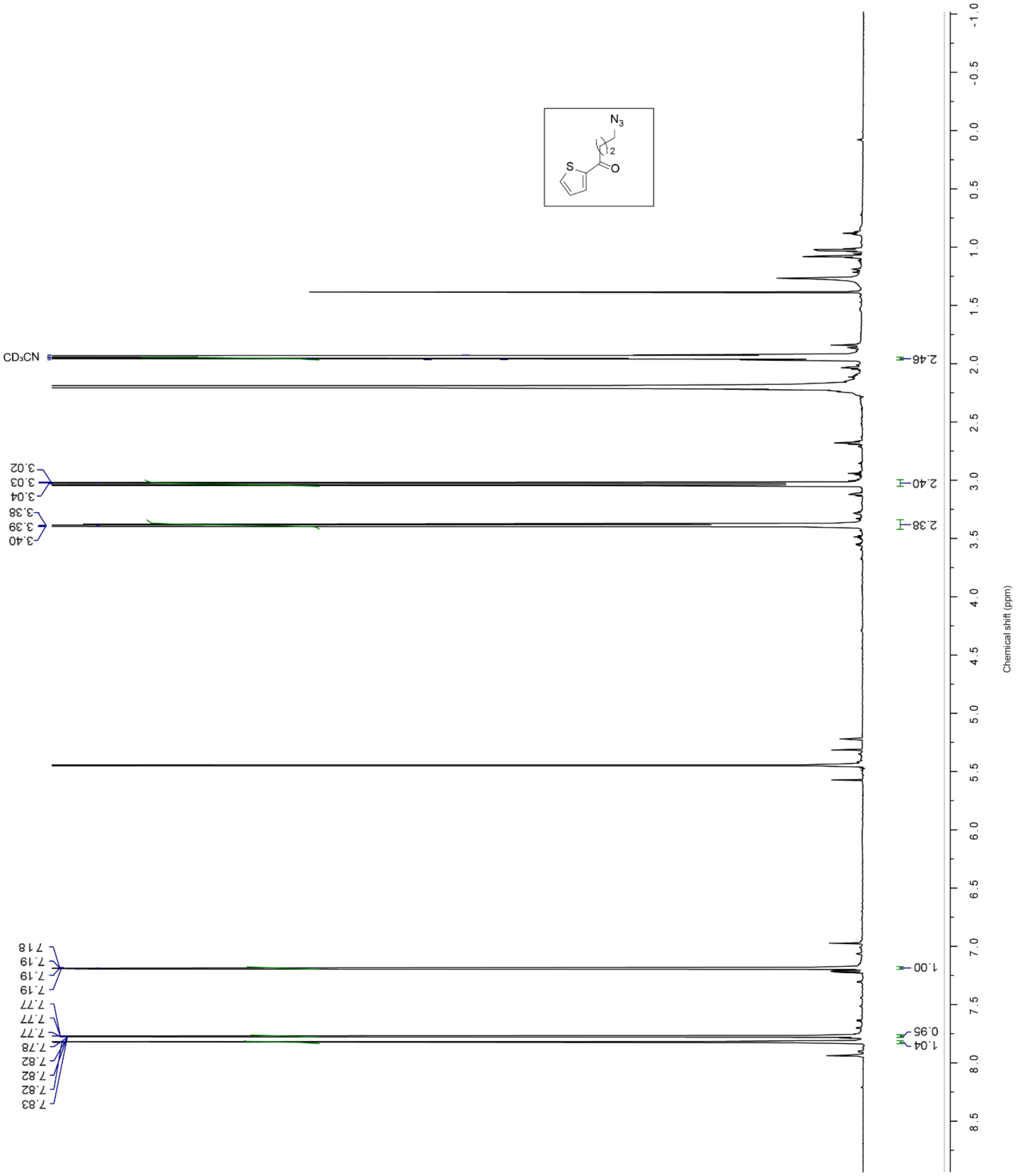

Figure S39. ^{1}H NMR spectrum of 4-azido-1-(thiophen-2-yl)butan-1-one in CD_3CN.

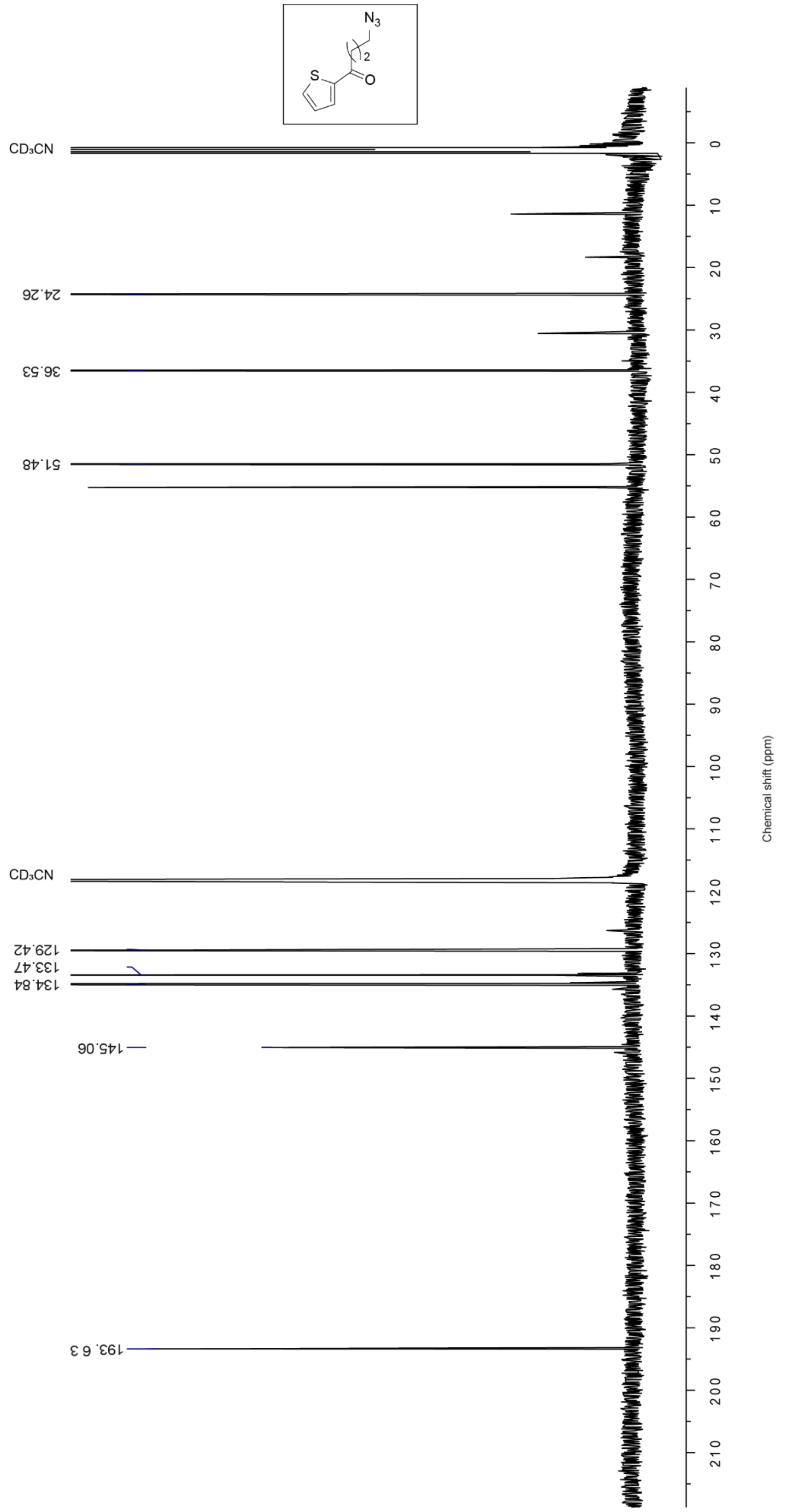

Figure S40. ${}^{13}C$ NMR spectrum of 4-azido-1-(thiophen-2-yl)butan-1-one in CD_3CN.

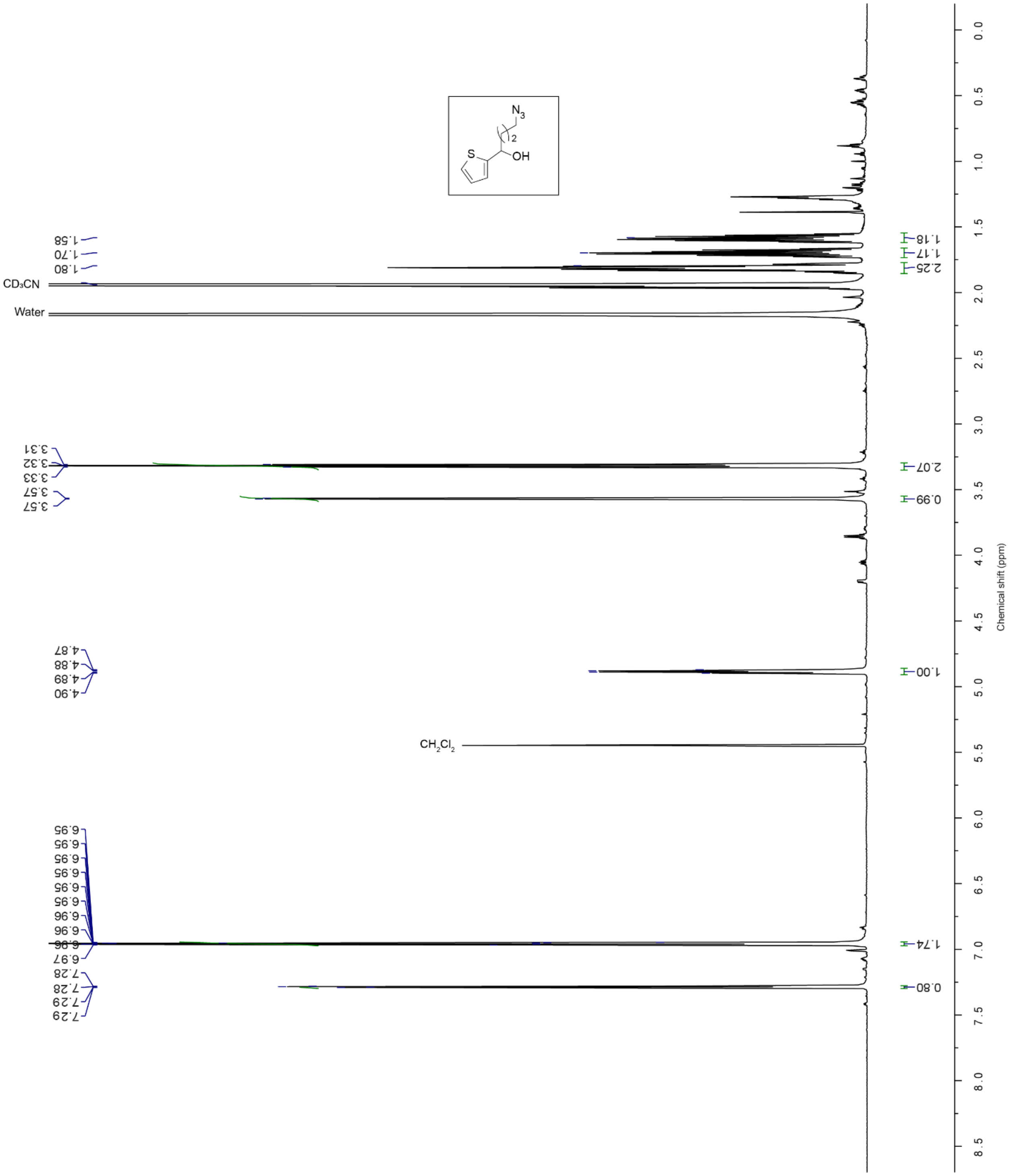

Figure S41. ^{1}H NMR spectrum of 4-azido-1-(thiophen-2-yl)butan-1-ol in CD_3CN.

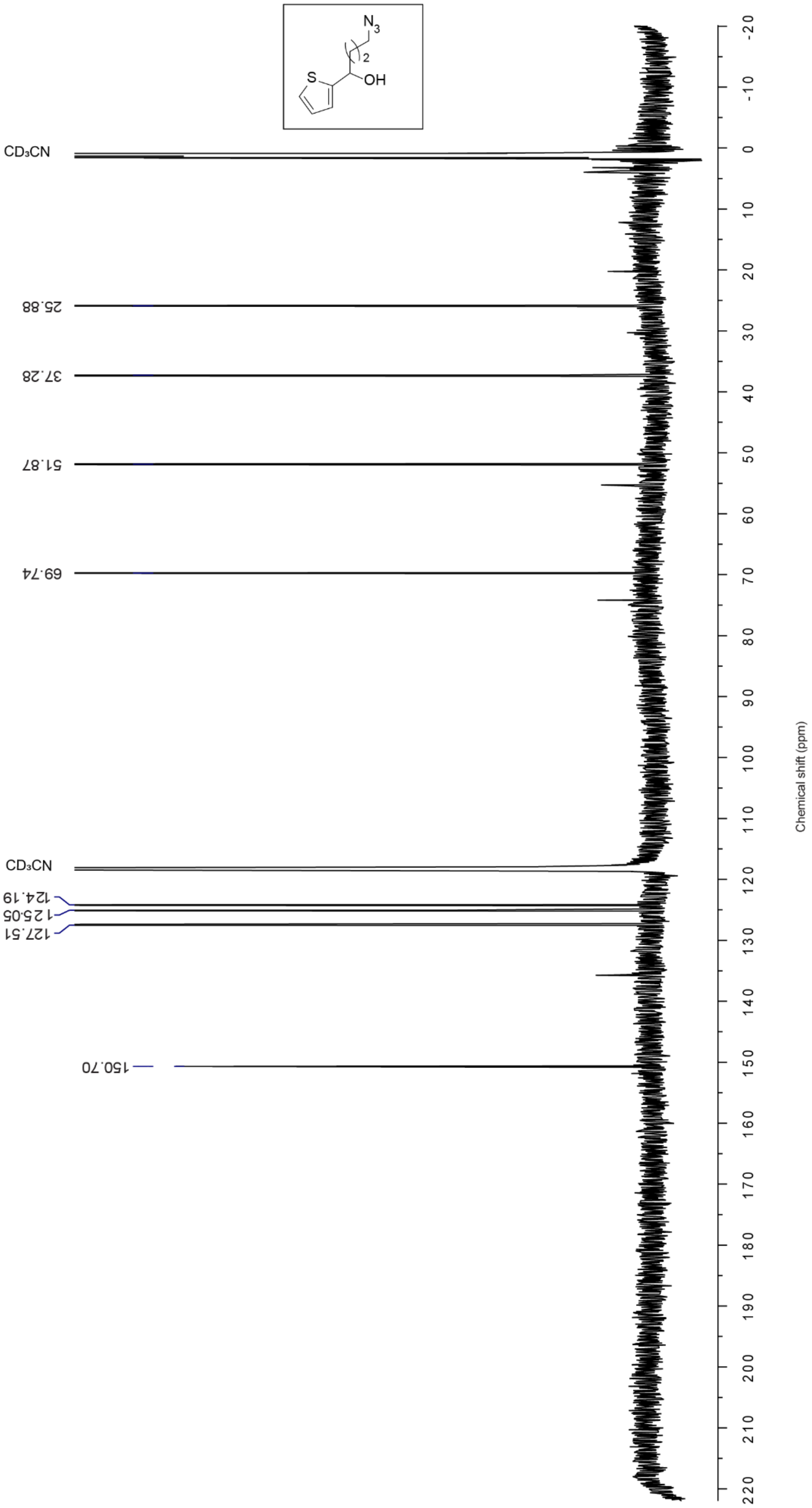

Figure S42. ^{13}C NMR spectrum of 4-azido-1-(thiophen-2-yl)butan-1-ol in CD_3CN.

References for Electronic Supplementary Information

(1) Nuruzzaman, M.; Colella, B. M.; Uzoewulu, C. P.; Meo, A. E.; Gross, E. J.; Ishizawa, S.; Sana, S.; Zhang, H.; Hoff, M. E.; Medlock, B. T. W.; Joyner, E. C.; Sato, S.; Ison, E. A.; Li, Z.; Ohata, J. Hexafluoroisopropanol as a Bioconjugation Medium of Ultrafast, Tryptophan-Selective Catalysis. *J. Am. Chem. Soc.* **2024**, *146* (10), 6773–6783. https://doi.org/10.1021/jacs.3c13447.

(2) Mori, S.; Takubo, M.; Makida, K.; Yanase, T.; Aoyagi, S.; Maegawa, T.; Monguchi, Y.; Sajiki, H. A Simple and Efficient Oxidation of Alcohols with Ruthenium on Carbon. *Chem. Commun.* **2009**, No. 34, 5159–5161. https://doi.org/10.1039/B908451G.

(3) Bagutski, V.; Ros, A.; Aggarwal, V. K. Improved Method for the Conversion of Pinacolboronic Esters into Trifluoroborate Salts: Facile Synthesis of Chiral Secondary and Tertiary Trifluoroborates. *Tetrahedron* **2009**, *65* (48), 9956–9960. https://doi.org/10.1016/j.tet.2009.10.002.

(4) Ramachandran, P. V.; Mitsuhashi, W. Preparation of Potassium Fluoroorganotrifluoroborates under Non-Etching Conditions *via* a Li-K Exchange. *Journal of Fluorine Chemistry* **2016**, *190*, 7–11. https://doi.org/10.1016/j.jfluchem.2016.07.023.

(5) Frohn, H.-J.; Franke, H.; Fritzen, P.; Bardin, V. V. (Fluoroorgano)Fluoroboranes and -Fluoroborates I: Synthesis and Spectroscopic Characterization of Potassium Fluoroaryltrifluoroborates and Fluoroaryldifluoroboranes. *Journal of Organometallic Chemistry* **2000**, *598* (1), 127–135. https://doi.org/10.1016/S0022-328X(99)00690-7.
